# Evaluation of newly synthesized 2-(thiophen-2-yl)-1H-indole derivatives as anticancer agents against HCT-116 cell proliferation via cell cycle arrest and down regulation of miR-25

**DOI:** 10.1038/s41598-024-68815-8

**Published:** 2024-08-29

**Authors:** Nagwa M. Abdelazeem, Shaimaa A. Gouhar, Cinderella A. Fahmy, Zeinab A. Elshahid, Marwa El-Hussieny

**Affiliations:** 1 Organometallic and Organometalloid Chemistry Department, National Research Centre, Dokki, 12622 Cairo Egypt; 2 Medical Biochemistry Department, Medicine and Clinical Studies Research Institute, National Research Centre, Dokki, 12622 Cairo Egypt; 3 Cancer Biology and Genetics Laboratory, Centre of Excellence for Advanced Sciences, National Research Centre, Dokki, 12622 Cairo Egypt; 4 Biochemistry Department, Biotechnology Research Institute, National Research Centre, Dokki, Cairo Egypt; 5 Chemistry of Natural and Microbial Products, National Research Centre, Dokki, 12622 Cairo Egypt

**Keywords:** (Methylene) bis (2-(thiophen-2-yl)-1*H*-indole), (2-(Thiophen-2-yl)-1*H*-indol-3-yl) methyl) aniline, SSA, Molecular docking, HCT-116, C-Myc, miR-25, miR-107, miR-30c, Biochemistry, Cancer

## Abstract

In the present study, we prepared new sixteen different derivatives. The first series were prepared (methylene)bis(2-(thiophen-2-yl)-1*H*-indole) derivatives which have (indole and thiophene rings) by excellent yield from the reaction (2 mmol) 2-(thiophen-2-yl)-1*H*-indole and (1 mmol) from aldehyde. The second series were synthesized (2-(thiophen-2-yl)-1*H*-indol-3-yl) methyl) aniline derivatives at a relatively low yield from multicomponent reaction of three components 2-(thiophen-2-yl)-1*H*-indole, *N*-methylaniline and desired aldehydes. The anticancer effect of the newly synthesized derivatives was determined against different cancers, colon, lung, breast and skin. The counter screening was done against normal Epithelial cells (RPE-1). The effect on cell cycle and mechanisms underlying of the antitumor effect were also studied. All new compounds were initially tested at a single dose of 100 μg/ml against this panel of 5 human tumor cell lines indicated that the compounds under investigation exhibit selective cytotoxicity against HCT-116 cell line and compounds (**4g, 4a, 4c**) showed potent anticancer activity against HCT-116 cell line with the inhibitory concentration IC_50_ values were, 7.1±0.07, 10.5± 0.07 and 11.9± 0.05 μΜ/ml respectively. Also, the active derivatives caused cell cycle arrest at the S and G2/M phase with significant(p < 0.0001) increase in the expression levels of tumor suppressors miR-30C, and miR-107 and a tremendous decrease in oncogenic miR-25, IL-6 and C-Myc levels. It is to conclude that the anticancer activity could be through direct interaction with tumor cell DNA like S-phase-dependent chemotherapy drugs. Which can interact with DNA or block DNA synthesis such as doxorubicin, cisplatin, or 5-fluorouracil and which were highly effective in killing the cancer cells. This data ensures the efficiency of the 3 analogues on inducing cell cycle arrest and preventing cancer cell growth. The altered expressions explained the molecular mechanisms through which the newly synthesized analogues exert their anticancer action.

## Introduction

Colorectal cancer is the third common malignancy and the second most common cause of cancer-related death worldwide^1^. It represents the most prevalent malignant cancer of the gastrointestinal tract affecting both men and women in the same way in both developed and underdeveloped countries^2^. Unfortunately, the rate of colorectal cancer incidence in young people is increasing substantially^3^. The risk of CRC development is determined by genetic predisposition combined with environmental influences that disrupt the mucosal barrier of the gastrointestinal tract leading to aberrant inflammation^4^. Tumor-associated inflammation contributes to tumor growth and progression through multiple mechanisms including increased cell proliferation and anti-apoptotic signaling, promotion of angiogenesis, tumor immune evasion and metastasis^5^. For example, aberrant expression of IL6, C-Myc and down streaming pathways are significantly involved in the progression of CRC, and mainly affecting apoptosis^6,7^. Also, specific microRNAs can act as either tumor suppressors or oncogenes depending on the cellular environment in which they are expressed. MiR-30C, miR-107 and miR-25 are among the microRNAs altered in CRC and their expression patterns are associated with diagnosis, prognosis and therapeutic outcome in CRC^8^. Regardless the cause of carcinogenesis or its pathways, till now, chemotherapeutic medicines and surgery are regarded as the most important colorectal cancer treatment modalities^9^. But one of the biggest challenges of using chemotherapy is the issue of medication toxicity and resistance^10^. Therefore, it is crucial to create new therapeutic medications that are effective like current ones without causing drug-induced toxicity.

**Figure 1:**
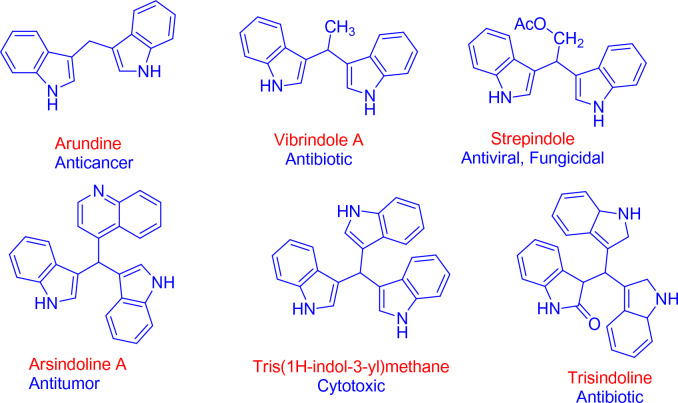
Representative analogues of naturally occurring 3,3′-BIMs with relevant bioactivity.

Of the most promising analogues heterocyclic compounds are crucial to both medical chemistry and organic chemistry as they have a broad biological effect. Sulphur, nitrogen, and oxygen-containing heterocyclic compounds have a promising pharmacological profiles and constantly attract the interest of medicinal chemists and researchers^11,12^. Indole derivatives have become an important group of heterocycles with nitrogen are well-known to have a wide range of biological and pharmacological effects^13^. For example, bis(indolyl)methane’s (BIMs) and its functionalized class of natural products were originally obtained from cruciferous plants and other terrestrial and marine species. Arundine, vibrindole A, streptindole, arsindoline A, tris(1*H*-indol-3-yl) methane, and trisindoline are important members of this class of natural compounds. This bioactive intermediate has a distinctive structure that includes two indole units that are responsible for a variety of vital biological activities, including anti-inflammatory, anticancer, antibiotic, insecticidal, antimicrobial, antifungal, antioxidant properties^14^, antibacterial, anticarcinogenic, genotoxic, and DNA-damaging properties^15^ (Fig. 1).

So numerous methods have been developed for their synthesis due to their diverse biological characteristics, using a variety of catalytic systems, such as amberlyst-15^16^, iodine^17^, boric acid^18^, fluoroboric acid^19^, sulfamic acid^20^, NbCl5^21^, silica sulfuric acid^22^, cellulose sulfuric acid^23^, zeolite^24^, and ceric ammonium nitrate^25^. Thiophene is another heterocyclic compound; it is a five-membered ring that contains a heteroatom of sulfur. Since it may be found in many pharmacologically effective drugs, it has become recognized as a key scaffold. Thiophene possesses a wide range of biological properties, including anticancer, antibacterial, anti-inflammatory, antidepressant analgesic, and anticonvulsant. As a result, it has acquired the name “wonder heterocycle”^26^.

In this article we prepared new series of (methylene)bis(2-(thiophen-2-yl)-1*H*-indole) which have (indole and thiophene) and (2-(thiophen-2-yl)-1*H*-indol-3-yl) methyl) aniline from the reaction of three components 2-(thiophen-2-yl)-1*H*-indole, *N*-methylaniline and desired aldehydes by using SSA as a catalyst. (SSA) has been employed as an effective heterogeneous catalyst. (SSA) is inexpensive, simple to make, recycles catalyst, and is simple to handle^27^. In this study, we aimed to determine the anticancer effect of the new synthesized compounds on CRC cells in vitro, with a particular emphasis on whether they exert the antitumor effects through regulating IL-6, C-Myc and selected miRNA expression patterns.

## Result and discussion

### Chemistry

By using one pot reaction we synthesized 3,3′-(phenyl methylene) bis(2-(thiophen-2-yl)-1*H*-indole) and *N*-methyl-4-(phenyl(2-(thiophen-2-yl)-1*H*-indol-3-yl) methyl) aniline derivatives. These compounds were prepared by reaction 2-(thiophen-2-yl)-1*H*-indole (2 mmol) (**1**), *N*-methylaniline (1 mmol) (**2**) and desired aldehydes (1 mmol) (**3a–k**).

**Figure 2:**
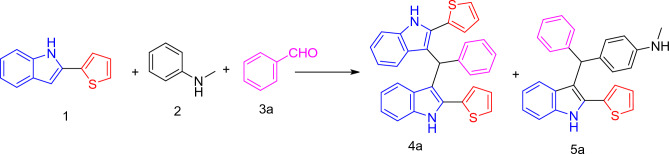
Preparation of 3,3′-(phenyl methylene) bis(2-(thiophen-2-yl)-1*H*-indole) (**4a**) and *N*-methyl-4-(phenyl(2-(thiophen-2-yl)-1*H*-indol-3-yl) methyl) aniline (**5a**).

We optimized the reaction conditions in various solvents, temperatures, and catalysts (Table 1) using benzaldehyde (**3a**) as the model substrate. (Fig. 2) When we used PPA/SiO_2_ (0.11) or HClO_4_/SiO_2_ (0.11) as catalyst in water under reflux for 10 min the reaction completion detected by *TLC* the yield of compound **4a** and **5a** approximately 60 and 15%, respectively. On the other hand, when the reaction is occurred by using SSA (0.11) as a catalyst without solvent the reaction completed after 30 min and the yield is relatively low. The reaction occurred by using SSA (0.11) in different solvents under reflux. The best yield is formed by using water as solvent and the reaction completed in the least time. When we decrease the concentration of SSA and fixed the solvent and the temperature the yield decreases. So, the best result was obtained by using SSA (0.11 mmol) under reflux in water the reaction completed after 5 min.

**Table 1:** Optimization of reaction conditions for the synthesis of 3,3′-(phenyl methylene) bis(2-(thiophen-2-yl)-1*H*-indole) (**4a**) and *N*-methyl-4-(phenyl(2-(thiophen-2-yl)-1*H*-indol-3-yl) methyl) aniline (**5a**) with different catalysts and solvents.

Entry	Catalyst (mol)	Solvent/°C	Time (min)	Yield 4a (%)	Yield 5a (%)
1	PPA/SiO_2_ (0.11)	H_2_O/reflux	10	60	15
2	HClO_4_/SiO_2_ (0.11)	H_2_O/reflux	10	60	15
3	**SSA (0.11)**	**H**_**2**_**O/reflux**	**5**	**77**	**20**
4	SSA (0.11)	–	30	29	25
5	SSA (0.11)	MeOH/reflux	15	40	14
6	SSA (0.11)	EtOH/reflux	5	64	15
7	SSA (0.11)	CHCl_3_/reflux	5	31	9
8	SSA (0.05)	H_2_O/reflux	5	66	20
9	SSA (0.025)	H_2_O/reflux	5	69	19

Significant values are in [bold].

**Figure 3:**
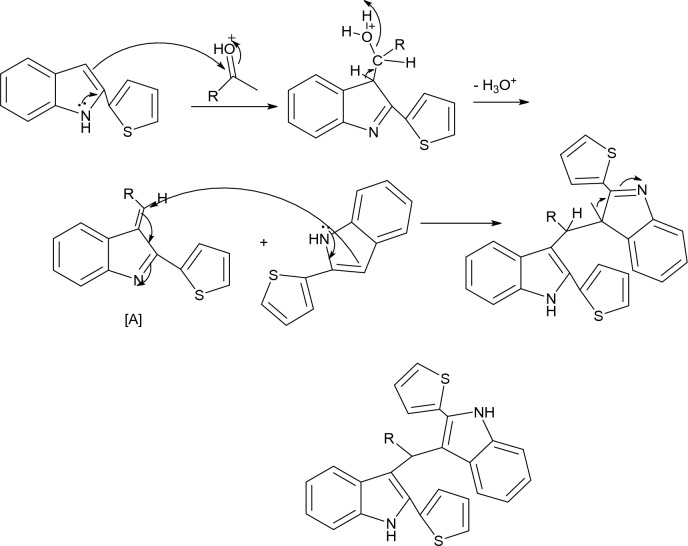
Plausible mechanism for the formation of (methylene)bis(2-(thiophen-2-yl)-1*H*-indole) catalyzed by SSA.

The major product (methylene)bis(2-(thiophen-2-yl)-1*H*-indole) by yield 77% is formed from the reaction (2 mmol) from 2-(thiophen-2-yl)-1*H*-indole (**1**) and (1 mmol) from aldehyde (**3**). Structure of synthesized compound was confirmed with ^1^HNMR and ^13^CNMR which appear that in ^1^HNMR single signals at 6.23 (1H, CH) and 11.43 (2H, 2NH) and in ^13^CNMR presence of signals at 41.23 (C-H). A plausible mechanism for the synthesis of (methylene)bis(2-(thiophen-2-yl)-1*H*-indole) catalyzed by SSA is explained (Fig. 3). The electrophilicity of carbonyl carbon has risen in the presence of the catalyst, and it quickly interacts with 2-(thiophen-2-yl)-1*H*-indole to produce the intermediate [**A**] through dehydration. The final product is produced in a good yield by reacting intermediate [A] with the second mole of 2-(thiophen-2-yl)-1*H*-indole^28^.

On the other hand, the minor product *N*-methyl-4-(phenyl(2-(thiophen-2-yl)-1*H*-indol-3-yl) methyl) aniline (**5a)** is formed from Multicomponent reaction between 2-(thiophen-2-yl)-1*H*-indole (**1**) (1 mmol), *N*-methylaniline (**2**) (1 mmol) and aldehyde (**3**) (1 mmol) by yield 20%. The most important data which are confirmed **5a** are two single signals at 2.80, 6.15 for (CH_3_ and CH), respectively. δ at 7.27 (s, 1H, NH) and 8.20 (s, 1H, NH). ^13^C NMR δ/ppm = 30.54 (CH_3_), 44.58 (CH), 110.79–147.72 (C aromatics).

**Figure 4:**
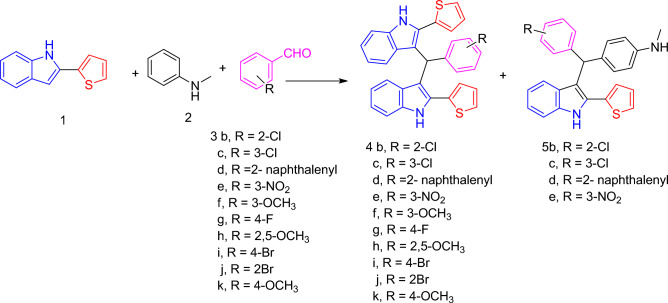
Synthesis of (methylene)bis(2-(thiophen-2-yl)-1*H*-indole) derivatives **4b–k**, and (2-(thiophen-2-yl)-1*H*-indol-3-yl)methyl)aniline derivatives **5b–e**.

When the reaction taken place by using desired aldehydes we separated (methylene)bis(2-(thiophen-2-yl)-1*H*-indole) derivatives by excellent yield **4b–k**, and (2-(thiophen-2-yl)-1*H*-indol-3-yl)methyl)aniline derivatives **5b–e** were isolated by a small percentage in cases of using 2chloro-, 3-chloro-, 2- naphthalenyl- and 3-nitro benzaldehyde (**3b–e**) (Fig. 4). The melting point and yield of synthesized compounds are listed in (Table 2). The structures of the new products were established based on elemental and spectroscopic (IR, ^1^H, ^13^C NMR) analyses (cf. Experimental).

**Table 2:** The reaction time, m.p. and yield for compounds **(4a–k)** and **(5a–5e)**.

Compd. No.	R	Time (min)	Yield (%)	MP (°c)
**4a**	H	5	77	250–252
**5a**			20	240–242
**4b**	2Cl	40	72	298–300
**5b**			13	178–180
**4c**	3Cl	40	65	188–190
**5c**			10	129–130
**4d**	2-naphthalenyl	10	83	198–200
**5d**			15	110–112
**4e**	3-NO_2_	15	80	129–130
**5e**			18	216–218
**4f**	3-OCH_3_	5	88	208–210
**4g**	4-F	15	68	128–130
**4h**	2,5-OCH_3_	10	85	136–138
**4i**	4-Br	30	67	198–200
**4j**	2-Br	15	77	148–150
**4k**	4-OCH_3_	30	80	108–110

### Biological studies

#### Evaluation of in vitro cytotoxic activity of analogues

**Figure 5:**
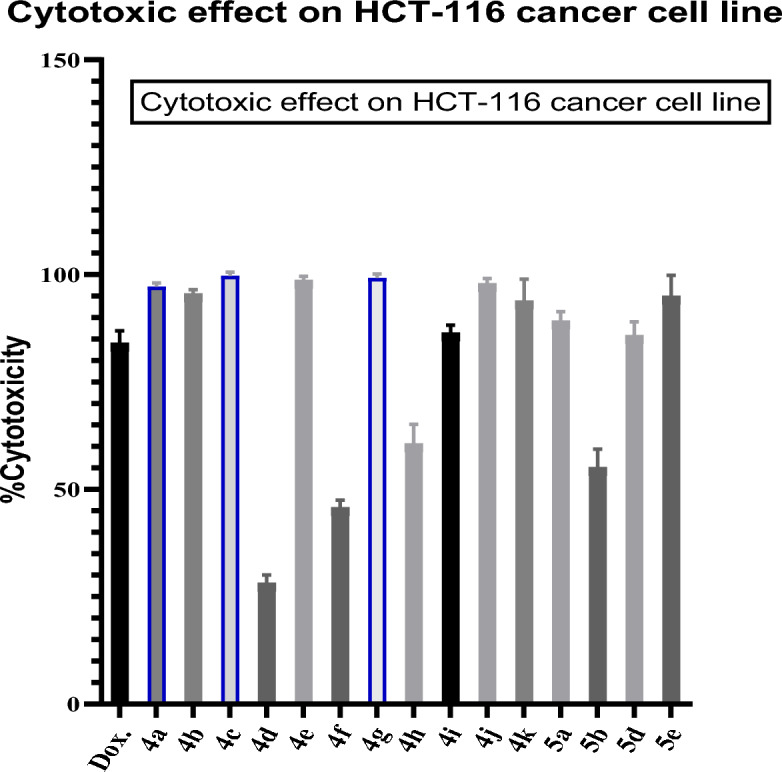
Average % cytotoxicity of different compounds on HCT-116 colon cancer cell line. Cell viability was determined after 48h and evaluated by MTT assay. Doxorubicin (DOX.) was used as positive control. Values are expressed as mean ± SD, n = 3 at a concentration of 100 µg/ml.

The screening of newly synthesized derivatives on different cancer monolayer cell lines is illustrated in Table 3. The initial screening of newly synthesized derivatives were performed at a single dose concentration of 100 ppm against a panel of 5 different cancer cell lines namely, human breast cancer (MCF-7), human colon cancer (HCT-116 and HT-29), human skin cancer (A375) and Human non-small cell Lung cancer (A549). Test compounds were administered to the cell lines and after 48h the percent cytotoxicity of treated cells was determined relative to untreated cells, Doxorubicin was used as positive control. Results of the initial single dose (100 µg/ml) testing for all 15 compounds against this panel of 5 human tumor cell lines indicated that the compounds under investigation exhibited selective cytotoxicity against HCT-116 and HT-29 cell line as compared to the reference doxorubicin, data are illustrated in (Fig. 5). Surprisingly, the compounds showed no activity against other cell lines (data not shown). HCT-116 cell line was the most sensitive to our compounds. Results showed that compounds (**4g**, **4a**, **4c**) showed potent anticancer activity against HCT-116 cells by recording the least The inhibitory concentration (IC_50_) values  (7.1± 0.07, 10.5±0.07 and 11.9±0.05) μΜ/ml compared to the positive control doxorubicin (98.7%) (Table 3, Fig. 5).

**Table 3:** Dose–response data of active compounds tested against HCT-116 cancer cell lines and the normal cell line RPE-1 after 48 h.

	Cp	HCT-116		RPE-1
		IC_50_ (µM/ml)	SI	IC_50_ (µM/ml)
	**Doxorubicin**	13.1 ± 0.03	0.65	8.55 ± 0.05
1	**4a**	10.5 ± 0.07	9.72	101.65 ± 2.7
2	**4b**	31.4 ± 0.05	2.78	87.43 ± 0.06
3	**4c**	11.9 ± 0.05	2.41	28.61 ± 0.02
4	**4d**	–	–	–
5	**4e**	62 ± 0.07	1.48	91.79 ± 2.6
6	**4f**	–	–	–
7	**4g**	7.1 ± 0.07	12.99	91.85 ± 1.3
8	**4h**	139.2 ± 0.05	1.19	165.92 ± 0.03
9	**4i**	38.9 ± 0.05	2.28	88.90 ± 8.9
10	**4j**	45.2 ± 0.05	1.59	72.11 ± 0.04
11	**4k**	42.2 ± 0.06	4.13	174.40 ± 1.3
12	**5a**	142.4 ± 0.03	0.68	97.78 ± 0.2
13	**5b**	114.01 ± 0.02	1.07	121.75 ± 0.5
14	**5d**	157.3 ± 0.04	0.70	110.87 ± 0.4
15	**5e**	131.2 ± 0.03	0.870	114.21 ± 3.4

IC50, Inhibitory concentration 50 in μg/ml; SI, selectivity index; NA, not active.

Doxorubicin was used as positive control drug.

#### Selectivity Index (SI)

Compounds that showed potent cytotoxic (≥ 60%) effect were subjected to dose response study to calculate the inhibitory concentration 50(IC_50_). **4g, 4a, 4c** compounds appeared also to be both the most potent and most selective against HCT-116 cells with an SI > 2.74 (SI: 13, 9.7 and 2.4, respectively) (Table 3). This finding encouraged us to further study the cellular mechanisms underlying the potent cytotoxic effect observed against colorectal cancer cells. The observation that these compounds possess high selectivity against cancer cells and low toxicity to normal cells highlights their potential as potent and safe anticancer drugs for further studies.

#### Molecular docking study

**Figure 6:**
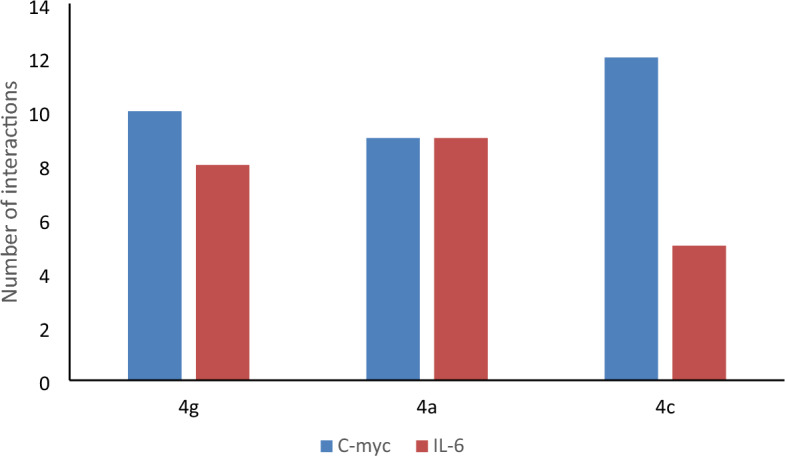
Number of interactions of the ligands with c-Myc and IL-6 proteins.

**Figure 7:**
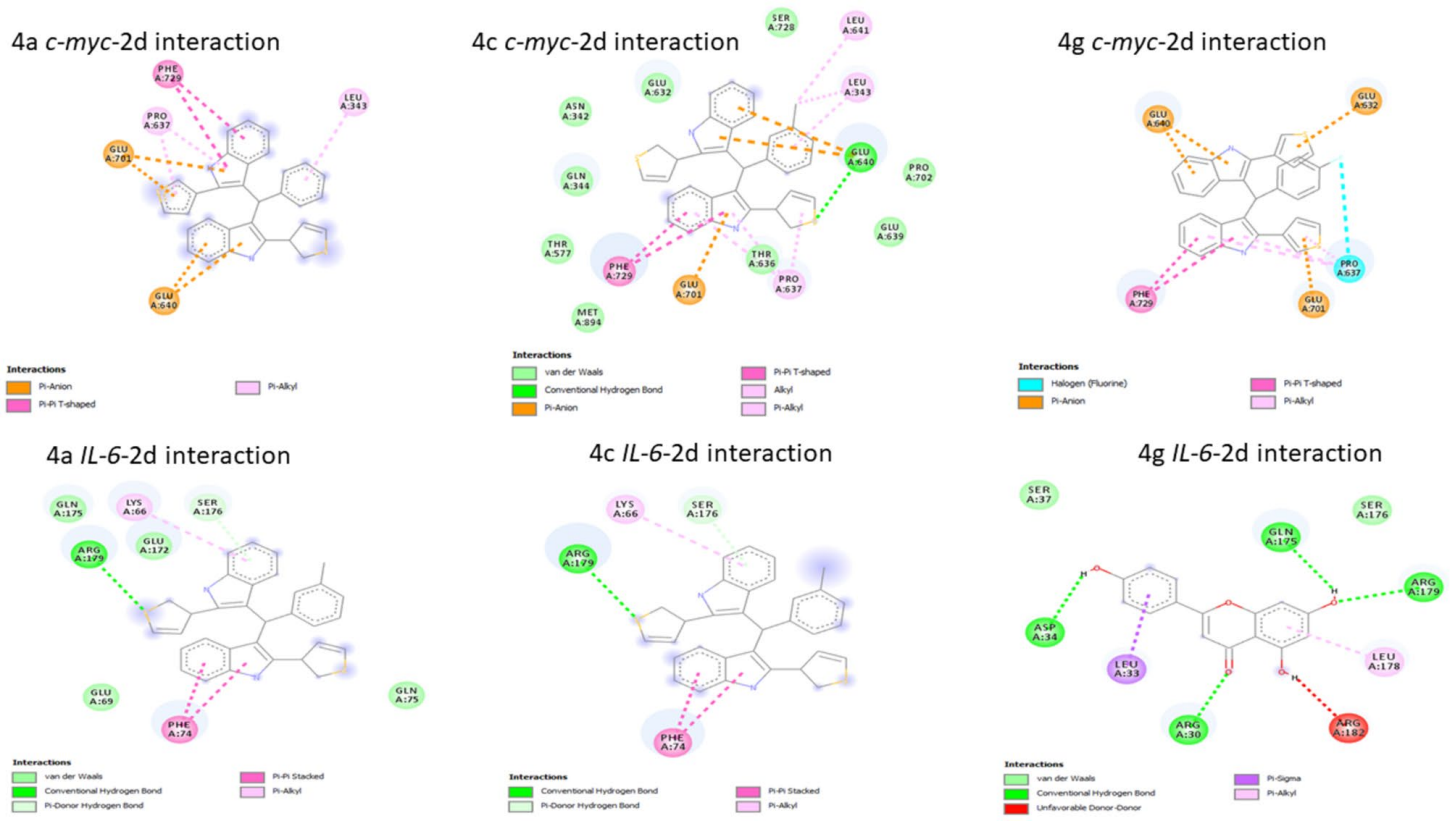
Two dimensional interactions of the best docked poses of the selected compounds with c-Myc and IL-6 proteins.

**Figure 8:**
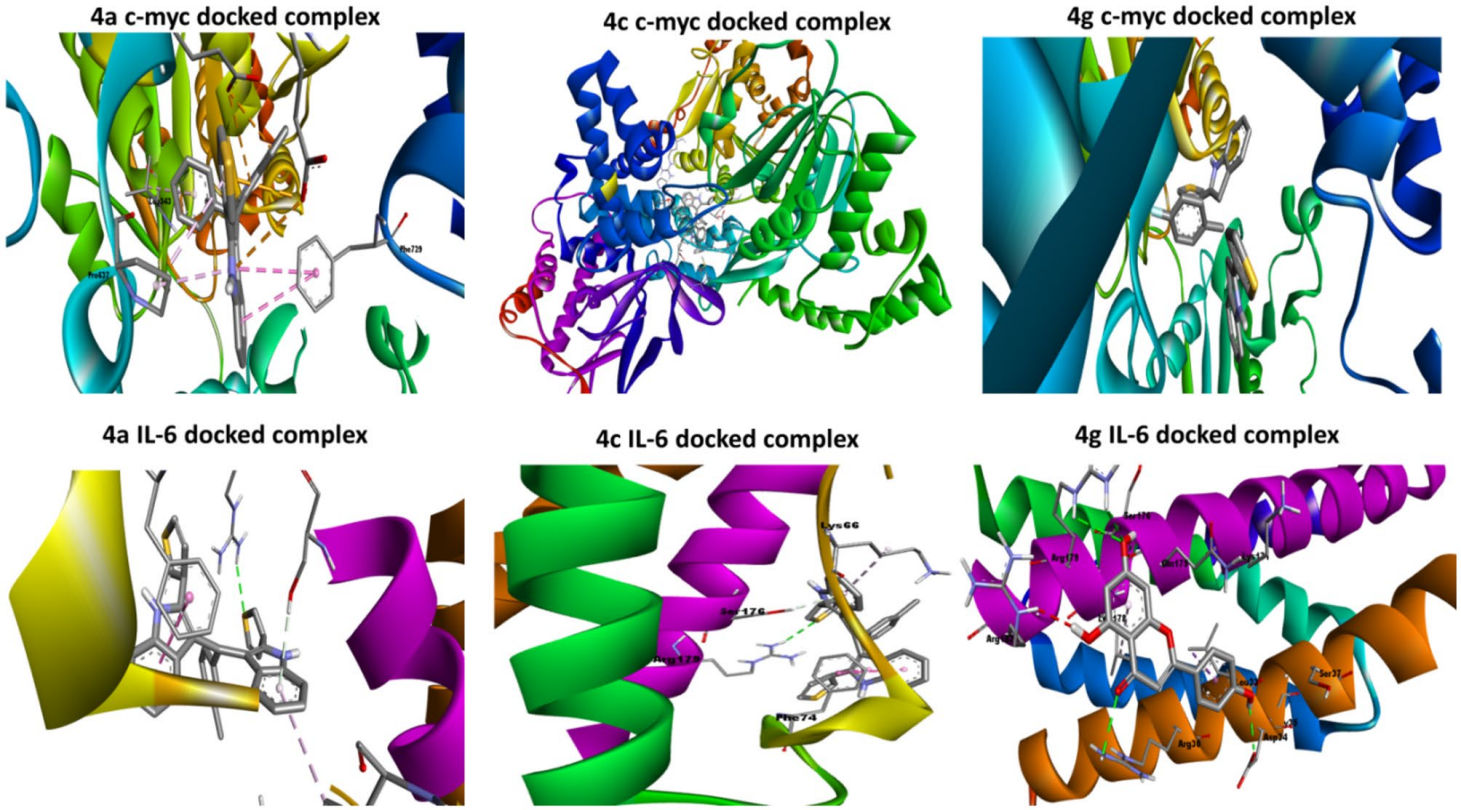
Three dimensional model of the best docked complexes inside the binding site of C-Myc and IL-6 proteins.

To determine the possible mechanisms of the anticancer activity of the best 3 synthesized compounds (**4g**, **4a**, **4c**) a molecular-docking study was done. We investigated their binding affinity against c-Myc and IL-6. These proteins are vital targets to generate anticancer agents. They were selected due to their vital role in the carcinogenesis process, consequently, targeting these proteins will be a good strategy for inventing efficient anticancer agents. The binding free energy of ligands and selected target proteins, number and type of interactions are summarized in Table 4, Fig. 6 while Figs. 7, 8 showed the two and three dimensional interactions between the ligands and the selected proteins. It was observed that all docked ligands had RMSD values < 2, which ensures proper docking protocol.

**Table 4:** Docking score and the best interactions for the ligand compounds with target proteins.

Compound	Target protein	Binding affinity (Kcal/mol)	RMSD	No. of interactions	Types of interactions	Amino acids forming interactions
4g	c-Myc	− 10.5	1.678	10	Halogen (Fluorine), Pi-Anion, Pi-Pi T shaped, Pi-Alkyl	Glu632, Pro637, Glu640, Glu701, Phe729
4a	c-Myc	− 9.1	1.532	9	Pi-Anion, Pi-Pi T shaped, Pi-Alkyl	Leu343, Pro637, Glu640, Glu701, Phe729
4c	c-Myc	− 9	0.503	12	Hydrogen bond, Pi-Anion, Pi-Pi T shaped, Pi-Alkyl, Alkyl, Van Der Waals	Asn342, Leu 343, Gln344, Thr577, Thr636, Pro637, Leu641, Glu639, Glu640, Glu701, Pro702, Ser728, Phe729, Met894,
4g	IL-6	− 8.3	0.964	8	Hydrogen bond, Pi-Alkyl, Van Der Waals, Pi-Sigma	Arg30, Leu33, Asp34, Ser37, Gln175, Ser176, Leu178, Arg179
4a	IL-6	− 7.5	1.851	9	Hydrogen bond, Pi-Donor Hydrogen bond, Pi-Alkyl, Van Der Waals, Pi-Alkyl, Pi-Pi Stacked	Lys66, Glu69, Phe74, Gln75, Glu172, Gln175, Ser176, Arg179
4c	IL-6	− 6.7	1.304	5	Hydrogen bond, Pi-Donor Hydrogen bond, Pi-Alkyl, Pi-Pi Stacked	Lys66, Phe74, Ser176, Arg179

All three ligands showed high binding affinity with both proteins which ensures their potency as inhibitors for c-Myc and IL-6 proteins. However, **4g** had the best binding free energy (− 10.5 and − 8.3 kcal/mol) with both proteins c-Myc and IL-6 respectively. Many types of interactions were observed such as Hydrogen bond, Pi-Anion, Pi-Pi T shaped, Pi-Alkyl, Alkyl, Van Der Waals which ensures the high binding affinity between synthesized compounds and target proteins. This diverse interaction profile contributes to the ligands’ high binding affinity and strengthens their binding to the protein surfaces. Pro637, Glu701, Glu640, and Phe729 of c-Myc protein were involved in many interactions with all three ligands while Arg179, Ser176 were the most interacting in case of IL-6 protein. Docking results ensured the potency of **4g**, **4a**, **4c** as anticancer agents especially in colorectal cancer and showed the possible mechanism through which these agents may exert their anticancer effect.

#### Cell cycle analysis and quantitative RT-PCR

**Figure 9:**
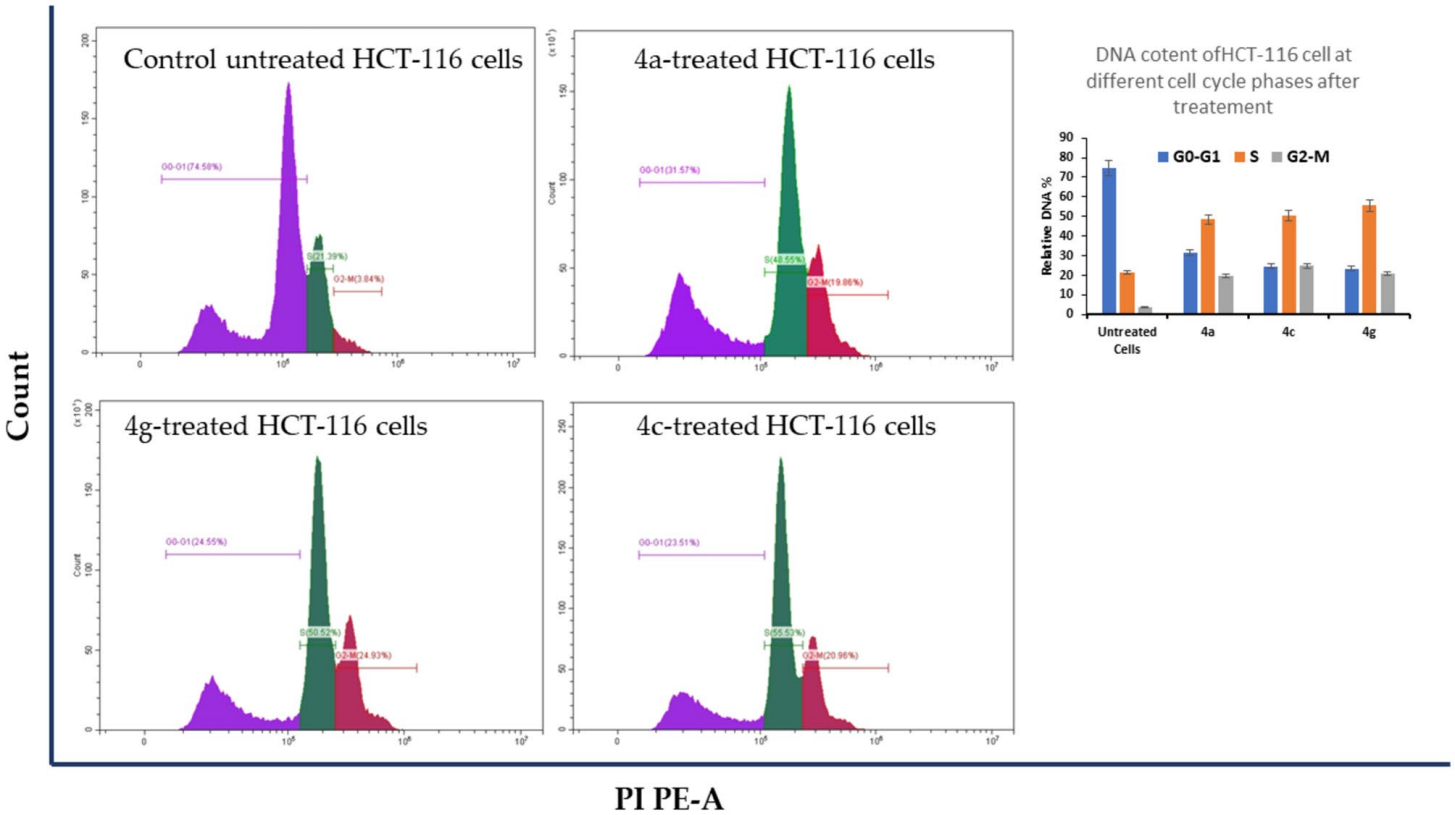
Histograms of cell cycle phase distribution by flow cytometry using PI staining and Relative DNA content for HCT-116 cells. (**A**) untreated HCT-116 cells; (**B**) HCT-116 cells treated with compound **4a**; (**C**) HCT-116 cells treated with compound **4c**; (**D**) HCT-116 cells treated with compound **4g**. The HCT-116 was plated at density of 1 × 10^6^ cells/well in 6-well plate. The cells were treated with IC_50_ value for each compound and incubated at 37 ℃ for 48h.

Numerous studies have been reported that some anti-cancer agents stimulated cell cycle arrest and thereby inducing apoptotic cell death. In particular, the uncontrolled cell cycle is a hallmark of tumor cells, and it is contributed to the progression and development of cancer^29–32^. Our results showed that treatment of HCT116 cells with **4g, 4c** and **4a** showed a significant increase in the percentage of cells in the S and G2/M phases compared to control (Fig. 9). These results indicated that our analogues induced cell cycle arrest at the S and G2/M phase. This would suggest that the compounds interfere with processes necessary for DNA replication or mitosis, effectively halting cell division. This is a potential anti-cancer mechanism, as uncontrolled cell division is a hallmark of cancer so, their anticancer activity could be through direct interaction with tumor cell DNA like S-phase-dependent chemotherapy drugs, which can interact with DNA or block DNA synthesis such as doxorubicin, cisplatin, or 5-fluorouracil and which were highly effective in killing the cancer cells^33–35^. Doxorubicin is known to induce cell cycle arrest in HCT116 cells, primarily at the G2/M checkpoint^36^. Also in other cell lines as MDA-MB-231 cells when treated with Dox resulted in a significantly higher percentage of cells in the G2M phase^37^. This arrest prevents cells from entering mitosis, thereby halting cell division and promoting cell death. Our results showed that treatment of HCT116 cells with **4g**, **4c**, and **4a** caused a significant increase in the percentage of cells in the S and G2/M phases compared to the control (Fig. 9). These findings suggest that our analogues, like doxorubicin, might induce cell cycle arrest, potentially at the S and G2/M phases. This data highlights the efficiency of the 3 analogues on inducing cell cycle arrest and preventing cancer cell growth.

**Figure 10:**
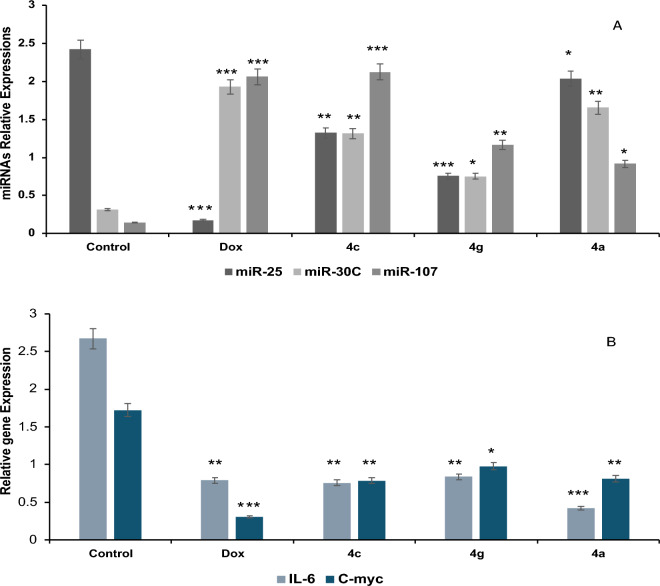
The effect of **4g**, **4c** and **4a** treatment on HCT116 cells by qRT-PCR. A) Relative expression of miR-25, miR-30C, and miR-107 in control and treated HCT116 cells. B) Relative expression of c-Myc and IL-6 in control and treated HCT116 cells. * means that p value < 0.05, ** means that p value < 0.01, *** means that p value < 0.001.

To further investigate the molecular mechanism through which our drugs exert their anticancer effect, a panel of miRNAs and genes which play a crucial role in colorectal cancer cell proliferation, tumorigenesis, and metastasis were selected: 3 miRNAs (miR-25, miR-30C, and miR-107) and two genes (IL-6 and C-Myc). It was observed a significant upregulation of miR-25, IL-6 and C-Myc (p < 0.001) while miR-30C, and miR-107 were downregulated (p < 0.001) in control HCT116 cells. The upregulation of miR-25, IL-6, and C-Myc in control HCT116 cells aligns with their oncogenic roles. Increased miR-25 promotes cell proliferation and inhibits apoptosis, while elevated IL-6 and C-Myc levels drive tumor growth and metastasis. Downregulation of tumor suppressors miR-30C and miR-107 further exacerbates the cancerous phenotype. The treatment of HCT116 cells with the 3 drugs caused a dramatic reversal by totally altering the expression pattern (p < 0.0001) as shown in Fig. 10. The treated cells showed a significant increase in the expression levels of tumor suppressors miR-30C, and miR-107 and a tremendous decrease in oncogenic miR-25, IL-6 and C-Myc levels. These results suggest that our drugs may act through multi-pronged mechanisms, simultaneously repressing oncogenes and activating tumor suppressors. This multifaceted approach could potentially overcome drug resistance and lead to more effective cancer treatment.

Our data are in accordance to many studies as the tumor suppressors miR-30C, and miR-107 are significantly downregulated in CRC both in cell lines and cancer tissues compared with matched normal adjacent tissue and this downregulation is related to shorter overall survival^38^. MiR-30C, and miR-107 are highly important for suppression of tumor metastasis, inhibition of cancer cell growth, migration and invasion^39–42^. In contrast, oncogenic miR-25 expression is significantly elevated in colorectal cancer and associated with tumor invasion, lymph node metastasis, distant metastasis and TNM. In addition, an increased level of miR-25 expression is associated with a poor overall survival of patients^43,44^. Regarding selected genes, the c-Myc gene is a nuclear transcription factor that mainly regulates cell growth, cell cycle, metabolism, and survival^45,46^ which is frequently overexpressed in approximately 70% of human cancer and related to metastatic progression of colorectal cancer^47–49^. Several experimental and clinical studies have linked the pleiotropic cytokine interleukin-6 (IL-6) to the pathogenesis of sporadic and inflammation-associated colorectal cancer (CRC). Increased IL-6 expression has been related to advanced stage of disease and decreased survival in CRC patients^50,51^. IL-6 level was significantly higher in patients with colorectal cancer than in normal controls. High levels of serum IL-6 were correlated with larger tumor size, elevated serum CRP levels, and liver metastases. IL-6 levels also increased in a stage-related manner^52–54^.

Based on the previous results, **4g**, **4c**, and **4a** exhibit potent and multi-pronged antitumor activity against HCT116 colorectal cancer cells. These promising agents directly interact with cellular DNA, causing cell cycle arrest at S and G2/M phases, effectively halting tumor cell proliferation. Furthermore, they upregulate tumor suppressor miRNAs miR-30C and miR-107, which inhibit cancer cell growth and metastasis. Concurrently, they downregulate oncogenic miR-25, c-Myc, and IL-6, known to promote tumorigenesis and shorten patient survival. This multifaceted approach, targeting both DNA and key regulatory molecules, suggests **4g**, **4c**, and **4a** hold significant potential for colorectal cancer treatment. Further investigations, including in vivo studies and target gene identification, are crucial to validate these findings and pave the way for future therapeutic applications.

## Materials and methods

### Chemistry

Melting points were determined with an electro thermal digital melting point apparatus (Electro-Thermal Engineering Ltd., Essex, United Kingdom). The IR spectra were recorded in KBr disks on a Pye Unicam SP 3300 and Shimadzu FT IR 8101 PC Infrared Spectrophotometers (Pye Unicam Ltd. Cambridge, England and Shimadzu, Tokyo, Japan, respectively). 1H and 13C NMR spectra were obtained from a Jeol ECA 500 MHz NMR Spectrometer (Tokyo, Japan) using deuterated dimethyl sulfoxide (d6-DMSO) as a solvent and (TMS) as an internal reference at 500, 125 MHz, respectively spectra were obtained from a JEOL ECA 500 MHz NMR Spectrometer at 200 MHz Mass spectra (EI-MS) were obtained with ISQ (Single Quadrupole MS, Thermo Scientific). Elemental analyses (C, H, N) results were recorded with Elementar Vario EL Germany. The recorded yields are of pure isolated materials obtained by column chromatography silica gel 60 (Merck) and thin layer chromatography (TLC) which was performed on Merck Kiesel gel F254 precoated plates (Merck, Darmstadt, Germany).

### General procedure for the optimization conditions

A mixture of 2-(thiophen-2-yl)-1*H*-indole (2 mmol) (**1**), *N*-methylaniline (1 mmol) (**2**) and benzaldehyde (1 mmol) (**3**) in water, methanol, ethanol, chloroform (5 ml) and without solvent and PPA/SiO_2_(0.11 mol %), HClO_4_/SiO_2_ (0.11 mol %) and SSA (0.11 mol%, 0.05 mol%, 0.025 mol%) catalysts was refluxed for an appropriate time as shown in Table 1. The mixture was stirred and heated under reflux for the appropriate time (Table 1). Until completion of the reaction (*TLC*), then purified the crude product by column chromatography using Petroleum ether/ethyl acetate as eluent.

**3,3′-(phenyl methylene)bis(2-(thiophen-2-yl)-1*H*-indole) (4a):** was separated by column chromatography using petroleum ether 60–80 °C/ethyl acetate (7:3, v/v) as an eluent, green crystals, yield 77%; m.p. 250–252 °C; IR: ʋ cm: 3389 (NH), 3052 (CH-_aliph_); ^1^H NMR (500 MHz, DMSO): δ/ppm = 6.23 (s, 1H, CH), 6.57 (m, 3H, Ar–H), 6.98 (m, 3H, Ar–H), 7.12 (d, 3H, J = 10.00 Hz, Ar–H), 7.25 (m, 2H, Ar–H), 7.35 (m, 4H, Ar–H), 7.46 (m, 4H, Ar–H), 11.43 (s, 2H, 2NH). ^13^C NMR (125 MHz, DMSO):41.23 (C–H), 111.84–145.12 (C-aromatics). Anal. Calcd for C_31_H_22_N_2_S_2_ (486.65): C, 76.51; H, 4.56; N, 5.76; S, 13.18 Found: C, 76.50; H 4.54; N, 5.75; S, 13.00%.

***N*-methyl-4-(phenyl(2-(thiophen-2-yl)-1*H*-indol-3-yl)methyl)aniline (5a):** was isolate by column chromatography using petroleum ether 60–80 °C/ethyl acetate (5:5, v/v) as an eluent, brown crystals, yield 20%; m.p. 240–242 °C; ^1^H NMR (500 MHz, CDCL_3_): δ/ppm = 2.80 (s, 3H, CH_3_), 6.15 (s, 1H, CH), 6.53 (t, 3H, J = 10.00 Hz, Ar–H), 6.86–6.93 (m, 5H, Ar–H), 7.15–7.19 (m, 2H, Ar–H), 7.27 (s, 1H, NH), 7.32–7.37 (m, 6H, Ar–H), 8.20 (s, 1H, NH).^13^C NMR (125 MHz, CDCL_3_): δ/ppm = 30.54 (CH_3_), 44.58 (CH), 110.79–147.72 (C aromatics). MS (EI, 70 eV): m/z 394 [M^+^], Anal. Calcd for C_26_H_22_N_2_S (394.53): C, 79.15; H, 5.62; N, 7.10; S, 8.13; Found: C, 79.14; H 5.60; N, 7.09; S, 8.03%.

### General procedure for synthesis of 3,3′-(aryl methylene) bis(2-(thiophen-2-yl)-1*H*-indole) derivatives (4b-k) and 4-((3-arylphenyl) (2-(thiophen-2-yl)-1*H*-indol-3-yl) methyl)-*N*-methylaniline derivatives (5b-e)

To the mixture of 2-(thiophen-2-yl)-1*H*-indole (1 mmol) (**1**), *N*-methylaniline (1 mmol) (**2**), and the desired aldehyde (1 mmol) (**3b–k**) in water, SSA (42.6 mg, 0.11 mol %) was added. The mixture was stirred and heated under reflux for the appropriate time (Table 1). Until completion of the reaction (*TLC*), then purified the crude product by column chromatography using Petroleum ether/ethyl acetate as eluent.

**3,3'-((2-chlorophenyl)methylene)bis(2-(thiophen-2-yl)-1*H*-indole) (4b):** was prepared by using eluent (5: 5), pale yellow crystals, yield 72%; m.p. 298–300 °C; IR: ʋ cm: 3394 (NH), 2927 (CH-_aliph_); ^1^H NMR (500 MHz, CDCL_3_): δ/ppm = 6.41 (s, 1H, CH), 6.83–6.92 (m, 3H, Ar–H), 7.10–7.20 (m, 10H, Ar–H), 7.25 (d, 1H, J = 10.00 Hz, Ar–H), 7.33–7.40 (m, 4H, Ar–H), 8.16 (s, 2H, 2NH). ^13^C NMR (125 MHz, CDCL_3_): δ/ppm = 39.33 (CH), 110.76- 142.43 (C aromatics). MS (EI, 70 eV): m/z 521[M^+^]; Anal. Calcd for C_31_H_21_ClN_2_S_2_ (521.09): C, 71.45; H, 4.06; N, 5.38; S, 12.31; Found: C, 71.44; H 4.10; N, 5.37; S, 12.28%.

**3,3′-((3-chlorophenyl)methylene)bis(2-(thiophen-2-yl)-1*H*-indole) (4c):** was isolated by using eluent (7:3), pale yellow crystals, yield 65%; m.p. 188–190 °C; IR: ʋ cm: 3395 (NH), 2922 (CH-_aliph_); ^1^H NMR (500 MHz, CDCL_3_): δ/ppm = 6.20 (s, 1H, CH), 6.83 (m, 2H, Ar–H), 6.89 (m, 4H, Ar–H), 7.18–7.27 (m, 8H, Ar–H), 7.35 (d, 3H, J = 5.00 Hz, Ar–H), 7.37 (m, 1H, Ar–H), 8.17 (s, 2H, 2NH). ^13^C NMR: 40.57 (CH), 110.83–146.64 (C aromatics). MS (EI, 70 eV): m/z 520 [M^-^]; Anal. Calcd for C_31_H_21_ClN_2_S_2_ (521.09): C, 71.45; H, 4.06; N, 5.38; S, 12.31 Found: C, 71.44; H 4.03; N, 5.37; S, 12.29%.

**3,3′-(naphthalen-2-ylmethylene)bis(2-(thiophen-2-yl)-1*H*-indole) (4d):** was prepared by using eluent (7:3), pale yellow crystals, yield 83%; m.p. 198–200 °C; IR: ʋ cm: 3395 (NH), 2849 (CH-_aliph_); ^1^H NMR (500 MHz, CDCL_3_): δ/ppm = 6.04 (s, 1H, CH), 6.56 (d, 2H, J = 10.00 Hz, Ar–H), 6.82 (t, 1H, J = 10.00 Hz, Ar–H), 6.99 (t, 1H, J = 10.00 Hz, Ar–H), 7.06–7.12 (m, 5H, Ar–H), 7.33–7.39 (m, 6H, Ar–H), 7.59 (s, 1H, Ar–H), 7.69–7.77 (m, 5H, Ar–H), 8.30 (s, 2H, 2NH). ^13^C NMR (125 MHz, CDCL_3_): δ/ppm = 41.04 (CH), 110.74–142.03 (C aromatics). MS (EI, 70 eV): m/z 536 [M^+^]; Anal. Calcd for C_35_H_24_N_2_S_2_ (536.71): C, 78.32; H, 4.51; N, 5.22; S, 11.95 Found: C, 78.31; H 4.51; N, 5.20; S, 11.92%.

**3,3′-((3-nitrophenyl)methylene)bis(2-(thiophen-2-yl)-1*H*-indole) (4e):** was prepared by using eluent (5: 5),pale yellow crystals, yield 80%; m.p. 129–130 °C; IR: ʋ cm: 3362 (NH), 2920 (CH-_aliph_); ^1^H NMR (500 MHz, CDCL_3_): δ/ppm = 6.35 (s, 1H, CH), 6.84–6.89 (m, 9H, Ar–H), 7.13 (m, 2H, Ar–H), 7.23 (s, 1H, Ar–H), 7.36 (m, 3H, Ar–H), 8.10 (m, 2H, Ar–H), 8.26 (s, 2H, 2NH). ^13^C NMR (125 MHz, CDCL_3_): δ/ppm = 40.63 (CH), 111.04, − 136.22 (C aromatics), 148.60 (C aromatic-NO_2_). MS (EI, 70 eV): m/z 531 [M^+^]; Anal. Calcd for C_31_H_21_N_3_O_2_S_2_ (531.65): C, 70.03; H, 3.98; N, 7.90; S, 12.06; Found: C, 70.01; H 3.97; N, 7.80; S, 12.03%.

**3,3′-((3-methoxyphenyl)methylene)bis(2-(thiophen-2-yl)-1*H*-indole) (4f):** was isolated by using eluent (6:4), green crystals, yield 88%; m.p. 208–210 °C; IR: ʋ cm: 3398 (NH), 2953 (CH-_aliph_); ^1^H NMR (500 MHz, DMSO): δ/ppm = 3.58 (s, 3H, OCH_3_), 6.24 (s, 1H, CH), 6.61–6.63 (m, 4H, Ar–H), 6.73 (d, 1H, J = 10.00 Hz, Ar–H), 6.83 (d, 1H, J = 10.00 Hz, Ar–H), 6.90–7.09 (m, 5H, Ar–H), 7.16–7.20 (m, 4H, Ar–H), 7.36 (d, 2H, J = 10.00 Hz, Ar–H), 7.46 (d, 1H, J = 10.00 Hz, Ar–H), 11.41 (s, 2H, 2NH). ^13^C NMR (125 MHz, DMSO): 47.29 (CH), 55.41 (OCH_3_), 111.83–146.74 (C aromatics), 159.89 (C aromatics-OCH_3_). MS (EI, 70 eV): m/z 517 [M^+^]; Anal. Calcd for C_32_H_24_N_2_OS_2_ (516.68): C, 74.39; H, 4.68; N, 5.42; S, 12.41; Found: C, 74.37; H 4.58; N, 5.40; S, 12.3%.

**3,3′-((4-fluorophenyl)methylene)bis(2-(thiophen-2-yl)-1*H*-indole) (4g):** was isolated by using eluent (8:2), green crystals, yield 68%; m.p. 128–130 °C; IR: ʋ cm: 3396 (NH), 2925 (CH-_aliph_); ^1^H NMR (500 MHz, DMSO): δ/ppm = 6.25 (s, 1H, CH), 6.55 (m, 2H, Ar–H), 6.60 (m, 2H, Ar–H), 6.98 (d, 3H, J = 5.00 Hz, Ar–H), 7.08 (m, 6H, Ar–H), 7.33 (t, 3H, J = 5.00 Hz, Ar–H), 7.46 (d, 2H, J = 5.00 Hz, Ar–H), 11.44 (s, 2H, 2NH). ^13^C NMR (125 MHz, DMSO): 47.22 (CH), 111.85—136.84 (C aromatics), 141.13 (C aromatic-F). MS (EI, 70 eV): m/z 503 [M^-^]; Anal. Calcd for C_31_H_21_FN_2_S_2_ (504.64): C, 73.78; H, 4.19; N, 5.55; S, 12.71; Found: C, 73.77; H 4.16; N, 5.50; S, 12.69%.

**3,3′-((2,5-dimethoxyphenyl)methylene)bis(2-(thiophen-2-yl)-1*H*-indole) (4h):** was isolated by using eluent (6:4), green crystals, yield 85%; m.p. 136–138 °C; IR: ʋ cm: 3400 (NH), 2928 (CH-_aliph_); ^1^H NMR (500 MHz, DMSO): δ/ppm = 3.71(s, 3H, OCH_3_), 3.82(s, 3H, OCH_3_) , 6.28 (s, 1H, CH), 6.44 (d, 1H, J = 5.00 Hz, Ar–H), 6.58 (d, 1H, J = 10.00 Hz, Ar–H), 6.70–6.75 (m, 2H, Ar–H), 6.81 (d, 1H, J = 5.00 Hz, Ar–H), 6.90 (d, 1H, J = 10.00 Hz, Ar–H), 7.00 (t, 2H, J = 7.50 Hz, Ar–H), 7.14 (d, 1H, J = 5.00 Hz, Ar–H), 7.17 (t, 2H, J = 5.00 Hz, Ar–H), 7.25 (d, 1H, J = 5.00 Hz, Ar–H), 7.32–7.36 (m, 3H, Ar–H), 7.57 (d, 2H, J = 5.00 Hz, Ar–H), 11.33 (s, 2H, 2NH). ^13^C NMR (125 MHz, DMSO): 47.43 (CH), 56.56 (OCH_3_), 56.89 (OCH_3_), 110.93–147.73 (C aromatics), 153.41 (C aromatic-OCH_3_), 153.70 (C aromatic-OCH_3_). MS (EI, 70 eV): m/z 546 [M^+^]; Anal. Calcd for C_33_H_26_N_2_O_2_S_2_ (546.70): C, 72.50; H, 4.79; N, 5.12; S, 11.73; Found: C, 72.47; H 4.77; N, 5.10; S, 11.70%.

**3,3′-((4-bromophenyl)methylene)bis(2-(thiophen-2-yl)-1*H*-indole) (4i):** was isolated by using eluent (5:5), brown crystals, yield 67%; m.p. 198–200 °C; IR: ʋ cm: 3396 (NH), 2922 (CH-_aliph_); ^1^H NMR (500 MHz, DMSO): δ/ppm = 6.19 (s, 1H, CH), 6.32 (m, 3H, Ar–H), 6.51 (d, 2H, J = 5.00 Hz, Ar–H), 6.65 (d, 2H, J = 10.00 Hz, Ar–H), 6.71 (t, 2H, J = 5.00 Hz, Ar–H), 6.95 (d, 3H, J = 5.00 Hz, Ar–H), 7.35–7.40 (m, 2H, Ar–H), 7.69 (d, 2H, J = 5.00 Hz, Ar–H), 7.83 (d, 2H, J = 5.00 Hz, Ar–H), 10.19 (s, 2H, 2NH). ^13^C NMR (125 MHz, DMSO): δ/ppm = 40.24 (CH), 110.55–143.45 (C aromatics). MS (EI, 70 eV): m/z 565 [M^+^]; Anal. Calcd for C_31_H_21_BrN_2_S_2_ (565.55): C, 65.84; H, 3.74; N, 4.95; S11.34; Found: C, 65.83; H 3.72; N, 4.94; S, 11.31%.

**3,3′-((2-bromophenyl)methylene)bis(2-(thiophen-2-yl)-1*H*-indole) (4j):** was prepared by using eluent (6:4), green crystals, yield 77%; m.p. 148–150 °C; IR: ʋ cm: 3397 (NH), 2922 (CH-_aliph_); ^1^H NMR (500 MHz, CDCL_3_): δ/ppm = 6.17 (s, 1H, CH), 6.54 (d, 1H, J = 5.00 Hz, Ar–H), 6.82 (t, 4H, J = 5.00 Hz, Ar–H), 7.02 (d, 1H, J = 10.00 Hz, Ar–H), 7.15 (t, 3H, J = 10.00 Hz, Ar–H), 7.22 (d, 2H, J = 5.00 Hz, Ar–H), 7.25 (m, 1H, Ar–H), 7.33–7.37 (m, 6H, Ar–H), 8.17 (s, 2H, 2NH). ^13^C NMR (125 MHz, CDCL_3_): δ/ppm = 40.28 (CH), 110.80–143.47 (C aromatics). MS (EI, 70 eV): m/z 563 [M^-2^]; Anal. Calcd for C_31_H_21_BrN_2_S_2_ (565.55): C, 65.84; H, 3.74; N, 4.95; S, 11.34; Found: C, 65.82; H 3.73; N, 4.93; S, 11.31%.

**3,3'-((4-methoxyphenyl)methylene)bis(2-(thiophen-2-yl)-1*H*-indole) (4k):** was prepared by using eluent (7:3), green crystals, yield 80%; m.p. 108–110 °C; IR: ʋ cm: 3393 (NH), 2948 (CH-_aliph_); ^1^H NMR (500 MHz, CDCL_3_): δ/ppm = 3.79 (s, 3H, OCH_3_), 6.17 (s, 1H, CH), 6.79–6.81 (m, 4H, Ar–H), 6.86–6.88 (m, 5H, Ar–H), 7.12 (t, 3H, J = 10.00 Hz, Ar–H), 7.18–7.21 (m, 4H, Ar–H), 7.32–7.36 (m, 2H, Ar–H), 8.14 (s, 2H, 2NH). ^13^C NMR (125 MHz, CDCL_3_): δ/ppm = 39.95 (CH), 55.29 (OCH_3_), 110.67–136.18 (C aqromatics), 158.06 (C aromatic-OCH_3_). MS (EI, 70 eV): m/z 515 [M^-^], Anal. Calcd for C_32_H_24_N_2_OS_2_ (516.68): C, 74.39; H, 4.68; N, 5.42; S, 12.41 Found: C, 74.38; H 4.37; N, 5.41; S, 12.40%.

**4-((2-chlorophenyl)(2-(thiophen-2-yl)-1*H*-indol-3-yl)methyl)-*N*-methylanilin (5b):** was isolated by using an eluent (4:6)), pale yellow crystals, yield 13%; m.p. 178–180 °C; ^1^H NMR (500 MHz, CDCL_3_): δ/ppm = 2.77 (s, 3H, CH_3_), 6.35 (s, 1H, CH), 6.13 (t, 3H, J = 10.00 Hz, Ar–H), 6.84–6.91 (m, 5H, Ar–H), 7.14–7.18 (m, 2H, Ar–H), 7.26 (s, 1H, NH), 7.30–7.37 (m, 5H, Ar–H), 8.23 (s, 1H, NH).^13^C NMR (125 MHz, CDCL_3_): δ/ppm = 30.94 (CH_3_), 44.98 (CH), 110.79- 147.72 (C aromatics). Anal. Calcd for C_26_H_21_ClN_2_S (428.98): C, 72.80; H, 4.93; N, 6.53; S, 7.47; Found: C, 72.78; H 4.91; N, 6.52; S, 7.45%.

**4-((3-chlorophenyl)(2-(thiophen-2-yl)-1*H*-indol-3-yl)methyl)-*N*-methylaniline (5c):** was isolated by using an eluent (4:6), pale yellow crystals, yield 10%; m.p. 129–130 °C; ^1^H NMR (500 MHz, DMSO): δ/ppm = 2.59 (s, 3H, CH_3_), 6.43 (s, 1H, CH), 6.75–7.31 (m, 15H, Ar–H), 7.61 (s, 1H, NH), 11.41 (s, 1H, NH). ^13^C NMR (125 MHz, DMSO): δ/ppm = 40.63 (CH), 111.04–145.60 (C aromatics). Anal. Calcd for C_26_H_21_ClN_2_S (428.98): C, 72.80; H, 4.93; N, 6.53; S, 7.47; Found: C, 72.79; H 4.91; N, 6.52, S; 7.44%.

***N*-methyl-4-(naphthalen-2-yl(2-(thiophen-2-yl)-1*H*-indol-3-yl)methyl)aniline (5d):** was isolated by using an eluent (4:6), pale yellow crystals, yield 15%; m.p. 110–112 °C; ^1^H NMR (500 MHz, CDCL_3_): δ/ppm = 2.82 (s, 3H, CH_3_), 6.04 (s, 1H, CH), 6.56 (d, 2H, J = 10.00 Hz, Ar–H), 6.82 (m, 1H, Ar–H), 7.06–7.11 (m, 7H, Ar–H), 7.33–7.39 (m, 7H, Ar–H), 7.69 (m, 1H, Ar–H), 7.59 (s, 1H, NH). ^13^C NMR (125 MHz, CDCL_3_) δ/ppm = 30.98 (CH_3_), 47.39 (CH), 110.73—148.01 (C-aromatics). Anal. Calcd for C_30_H_24_N_2_S (444.59): C, 81.05; H, 5.44; N, 6.30; S, 7.21; Found: C, 81.02; H 5.43; N, 6.29; S, 7.18%.

***N*-methyl-4-((3-nitrophenyl)(2-(thiophen-2-yl)-1*H*-indol-3-yl)methyl)aniline (5e):** was isolated by using an eluent (5:5)), pale yellow crystals, yield 18%; m.p. 216–218 °C; ^1^H NMR (500 MHz, DMSO): δ/ppm = 2.60 (s, 3H, CH_3_), 5.97 (s, 1H, CH), 6.46 (d, 2H, J = 5.00 Hz, Ar–H), 6.75 (m, 2H, Ar–H), 6.88 (d, 2H, J = 5.00 Hz, Ar–H), 7.02 (m, 2H, Ar–H), 7.16 (d, 1H, J = 5.00 Hz, Ar–H), 7.22 (m, 1H, Ar–H), 7.35 (d, 2H, J = 10.00 Hz, Ar–H), 7.53 (m, 1H, Ar–H), 7.60 (s, 1H, NH), 7.85–8.03 (m, 2H, Ar–H), 11.47 (s, 1H, NH).^13^C NMR (125 MHz, DMSO): δ/ppm = 30.29 (CH_3_), 44.16 (CH), 111.96–148.25 (C aromatics), 149.04 (C aromatic-NO_2_). MS (EI, 70 eV): m/z 439 [M^+^]; Anal. Calcd for C_26_H_21_N_3_O_2_S (439.53): C, 71.05; H, 4.82; N, 9.56; S, 7.30; Found: C, 71.04; H 4.81; N, 9.55; S, 7.27%.

### Biology

#### Cell culture

The human (HCT-116, HT-29, A549, MCF-7 and A375) cancer cell lines and RPE-1, normal cell line were maintained in DMEM-F12 medium. The media was supplemented with 10% fetal bovine serum at 37 °C in 5% CO_2_ and 95% humidity. Cells were sub-cultured using trypsin versene 0.15%. The anticancer activity was conducted at the Bioassay-Cell Culture Laboratory, National Research Center, Cairo, Egypt, for in vitro primary antitumor screening on Human Colorectal carcinoma (HCT-116, HT-29) and Normal Human Retinal Epithelial (BJ-1) cell line. All cell lines were kindly provided by Professor Stig Linder, Oncology and Pathology department, Karolinska Institute, Stockholm, Sweden, originally obtained from ATCC.

#### Cytotoxicity on cancer monolayers

The cytotoxic assay on cancer cell lines was done according to the method of^55,56^ with slight modifications. All the following procedures were performed in a sterile area using a laminar flow cabinet bio-safety class II level (Baker, SG403INT, and Sanford, ME, USA). Briefly, after 24 h of seeding 10,000 cells per well for HCT-116, HT-29, A549, MCF-7 and A375 cancer cell lines (in 96 well plates), the medium was changed to fresh medium and cells were treated with 100 μg/ml final concentration of the different compounds in triplicates for 48 h. 100 μM doxorubicin was used as positive control and 0.5% DMSO was used as negative control. Cell viability was determined using the MTT (3-(4, 5-dimethylthiazol-2-yl)-2,5-diphenyltetrazolium bromide) assay as described previously^57^.

The percent cytotoxicity was calculated according to the following equation:

$\% cytotoxicity\, = \,[1 - \left( {AV_{x} /AV_{NC} } \right)]\, \times \,100$ where AV: average, X: absorbance of sample well, NC: absorbance of negative control measured at 595 nm with reference at 690 nm.

#### Determination of IC_50_ values

Compounds that showed highly active cytotoxicity on different tested cell lines were selected for dose response study at different concentrations. The final tested concentrations were 100, 50, 25, 12.5, 6.25 μg/ml and up to 0.78 μg/ml, in triplicates. The IC_50_ values were calculated using the concentration–response curve fit to the non-linear regression model using GraphPad Prism® v6.0 software (GraphPad Software Inc., San Diego, CA, USA).

#### Selectivity Index (SI)

The selectivity index (SI) indicates the cytotoxic selectivity (i.e., safety) of different compounds against HCT-116 cancer cells versus normal cells (RPE-1). SI = IC_**50**_ of compound in the normal cell line**/**IC_**50**_ of the same compound in the cancer cell line. The higher the SI value, the higher the safety of the compound. Compounds possessing SI value > 2 are considered^58,59^.

#### Molecular docking study

The structures of all synthesized compounds (ligands) were displayed by the ChemDraw JS sample online page. The ligands were prepared for docking by optimization, and energy minimization using the PyRx software^60^. The three-dimensional crystal structures of the target proteins were downloaded from Protein Data Bank (PDB): HUMAN INTERLEUKIN-6 (PDB ID: 1ALU), c-Myc (PDB ID: 5VHE). The target proteins were prepared for docking by removing water and ions, adding hydrogen, and the allocation of charge. The preparation process was achieved by UCSF Chimera version 1.11.2^61^. The active sites were distinguished with proper grid boxes round the bound crystallized ligands. The docking study was achieved by PyRx software^60^, where the less energy isomers were generated and the best ligand-receptor complexes were assessed according to binding affinity. The Discovery Studio Visualizer program was used for visualization of docked complexes^62^.

#### Cell cycle analysis by flow cytometry

Cell cycle analysis was carried out to detect the probable changes in the cell cycle phases in control and treated cells. 1 × 10^6^ of HCT116 cells suspended in 0.5 ml 1 × DPBS and were aspirated several times with Pasteur pipet and fixed with 70% ethanol on ice for 2 h and centrifuged for 5 min at 300 × g. Cell pellets were re-suspended in 5 ml 1 × DPBS for 30 s and centrifuged at 300 × g for 5 min then re-suspended in 1 ml of PI staining solution and kept in the dark at room temperature for 30 min. Finally, cells were then transferred to the CytoFLEX Flow Cytometer (Beckman Coulter Life Sciences, USA) to measure the cell fluorescence (BECKMAN COULTER Inc., Cairo, Egypt and Cat. No. 4238055-CB) for cell cycle analysis. The percentage of cells in G0/G1, S, and G2/M phases of the cell cycle was calculated using CytExpert Software^63,64^.

#### RNA extraction and quantitative real time PCR

After treatment with **4g**, **4a**, and **4c** for 48h, HCT116 cells were harvested and total RNAs including small sequence RNAs (miRNAs) were extracted from cell pellets using miRNeasy Mini Kit (Qiagen, Hilden, Germany) according to the manufacturer’s protocol. RNAs were quantified at A260/A280 nm using a NanoDrop1000 (Thermo Fisher Scientific GmbH, Dreieich, Germany). Extracted RNAs were reverse-transcribed, using miScript everse transcription Kit (Qiagen) for miRNAs and RevertAid Reverse Transcriptase kit (Thermo Scientific) for genes. The differential expression levels of the selected miRNAs and genes were analyzed by quantitative real-time PCR (qRT-PCR) using miScript SYBR green PCR kit (Qiagen) on a real-time PCR machine (Agilent, Mx3000P, CA, USA). RT-PCR was performed in triplicates using the following cycling conditions: 95 °C for 15 min, followed by 40 cycles of 94 °C for 15 s, 55 °C for 30 s, and 70 °C for 30 s. All samples were normalized to the internal control RNU6 for miRNAs and GAPDH for genes and relative expression levels were calculated with the 2−ΔΔCt method and represented as fold change.

### Ethics approval

The experimental design related to human normal cells was carried out following the guidelines approved by the Institutional human normal cells Ethical Committee of National Research Centre, Dokki, Giza, Egypt (No:089122023,26-2-2023).

## Conclusions

The newly synthesized derivatives were screened against a panel of cancer cell lines, namely human colon cancer (HCT-116 and HT-29) human breast cancer (MCF-7), human non-small cell lung cancer (A549) and human skin cancer (A375). Compounds (**4g**, **4a**, **4c**) were the most potent active analogs with potent selective cytotoxicity against the colon cancer (HCT-116) cells. Notably, they showed no activity against other tested cancer lines, suggesting potential for targeted therapy with reduced side effects. These compounds have multiple mechanisms of action including direct DNA interaction, cell cycle arrest at the S and G2/M phase, and modulation of key miRNAs and protein expression. Targeting multiple pathways simultaneously strengthens the case for these compounds as promising anticancer agents. The upregulation of tumor suppressors miR-30C, and miR-107 and the downregulation of oncogenic factors like miR-25, IL-6 and C-Myc aligns with established targets for anticancer therapy, suggesting potential clinical relevance. The study provides compelling evidence for the potential of **4g**, **4c**, and **4a** as anticancer agents. Further research focusing on detailed mechanistic investigations, in vivo validation, and addressing potential limitations is crucial to translate these promising findings into clinical applications.

### Supplementary Information

Supplementary Information.

## Data Availability

Data is provided within the supplementary information files.
