# Supplementary material for: Evaluation of newly synthesized 2-(thiophen-2-yl)-1H-indole derivatives as anticancer agents against HCT-116 cell proliferation via cell cycle arrest and down regulation of miR-25
Source: Sci Rep. 2024 Aug 29;14:20045. doi: 10.1038/s41598-024-68815-8 (PMC11362284; doi:10.1038/s41598-024-68815-8)
Supplement: Supplementary file 1 — Supplementary Information. [file 41598_2024_68815_MOESM1_ESM.docx]

**Supporting Information file**

**Synthesis, molecular docking, and anticancer effect of newly synthesized 2-(thiophen-2-yl)-1*H*-indole derivatives: suppression of HCT-116 Cell proliferation, cell cycle arrest and downregulation of miR-25, C-Myc and IL-6**

Nagwa M. Abdelazeem^1^, Shaimaa A. Gouhar^2^, Cinderella A. Fahmy^3,4^, Zeinab A. Elshahid^5*^, Marwa El-Hussieny^1*^

^1^Organometallic and Organometalloid Chemistry Department, National Research Centre, Dokki 12622, Cairo, Egypt.

^2^Medical Biochemistry Department, Medicine and Clinical Studies Research Institute, National Research Centre, Dokki 12622, Cairo, Egypt. E-mail: [ssadek007@gmail.com](mailto:ssadek007@gmail.com) ORCID ID: 0000-0003-1879-186X.

^3^Cancer Biology and Genetics Laboratory, Centre of Excellence for Advanced Sciences, National Research Centre, Dokki 12622, Cairo, Egypt. ORCID ID: 0009-0008-1713-9136

^4^Biochemistry Department, Biotechnology Research Institute, National Research Centre, Dokki, Cairo, Egypt. ORCID ID: 0009-0008-1713-9136

^5^Chemistry of Natural and Microbial Products, National Research Center. Dokki 12622, Cairo, Egypt. E-mail: [dr.z.a.elshahid@gmail.com](mailto:dr.z.a.elshahid@gmail.com) ORCID ID: 0000-0001-8237-9568.

***S1: Experimental***

**Chemistry**

Melting points were determined with an electro thermal digital melting point apparatus (Electro-Thermal Engineering Ltd., Essex, United Kingdom). The IR spectra were recorded in KBr disks on a Pye Unicam SP 3300 and Shimadzu FT IR 8101 PC Infrared Spectrophotometers (Pye Unicam Ltd. Cambridge, England and Shimadzu, Tokyo, Japan, respectively). 1H and 13C NMR spectra were obtained from a Jeol ECA 500 MHz NMR Spectrometer (Tokyo, Japan) using deuterated dimethylsulphoxide (d6-DMSO) as a solvent and (TMS) as an internal reference at 500, 125 MHz, respectively spectra were obtained from a Jeol ECA 500 MHz NMR Spectrometer at 200 MHz. Mass spectra (EI-MS) were obtained with ISQ (Single Quadrupole MS, Thermo Scientific). Elemental analyses (C, H, N) results were recorded with Elementar Vario EL Germany, phosphorus was measured by spectrophotometric methods. The recorded yields are of pure isolated materials obtained by column chromatography silica gel 60 (Merck) and thin layer chromatography (TLC) which was performed on Merck Kiesel gel F254 precoated plates (Merck, Darmstadt, Germany).

***S2. Copies of IR, ^1^H NMR and ^13^C NMR spectra of final compounds***

IR and ^1^H NMR for compound 4a


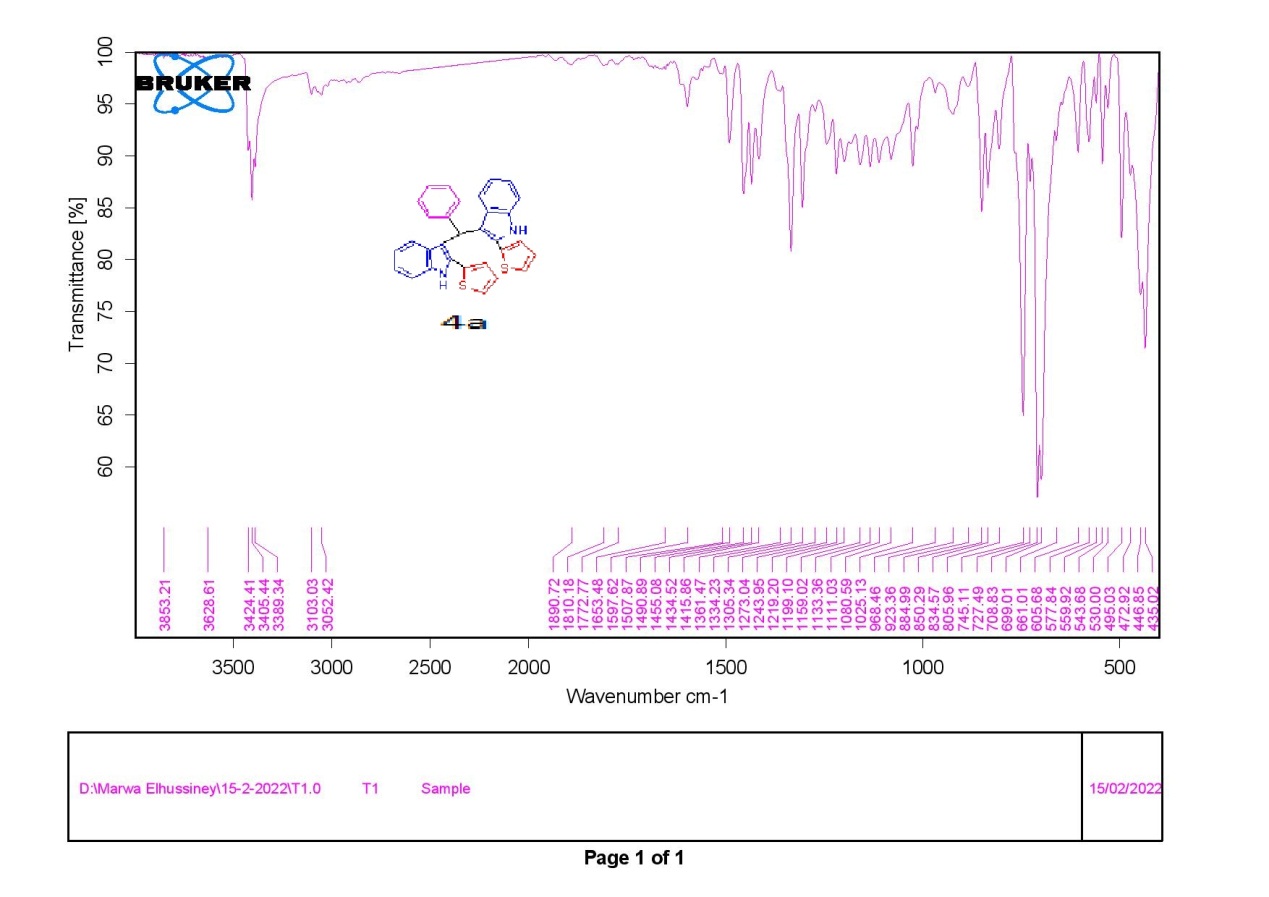


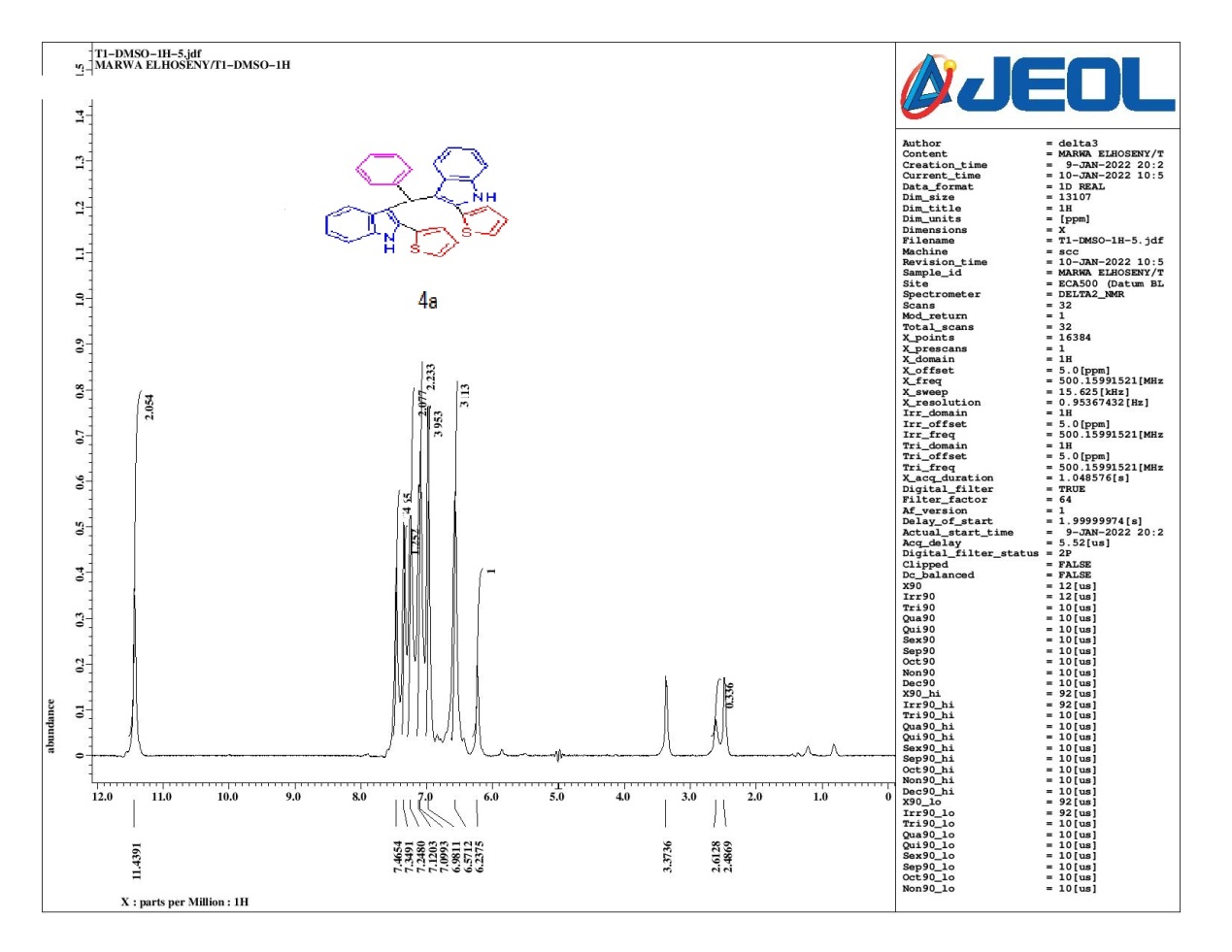


IR , ^1^H NMR and^13^C NMR for compound 4b


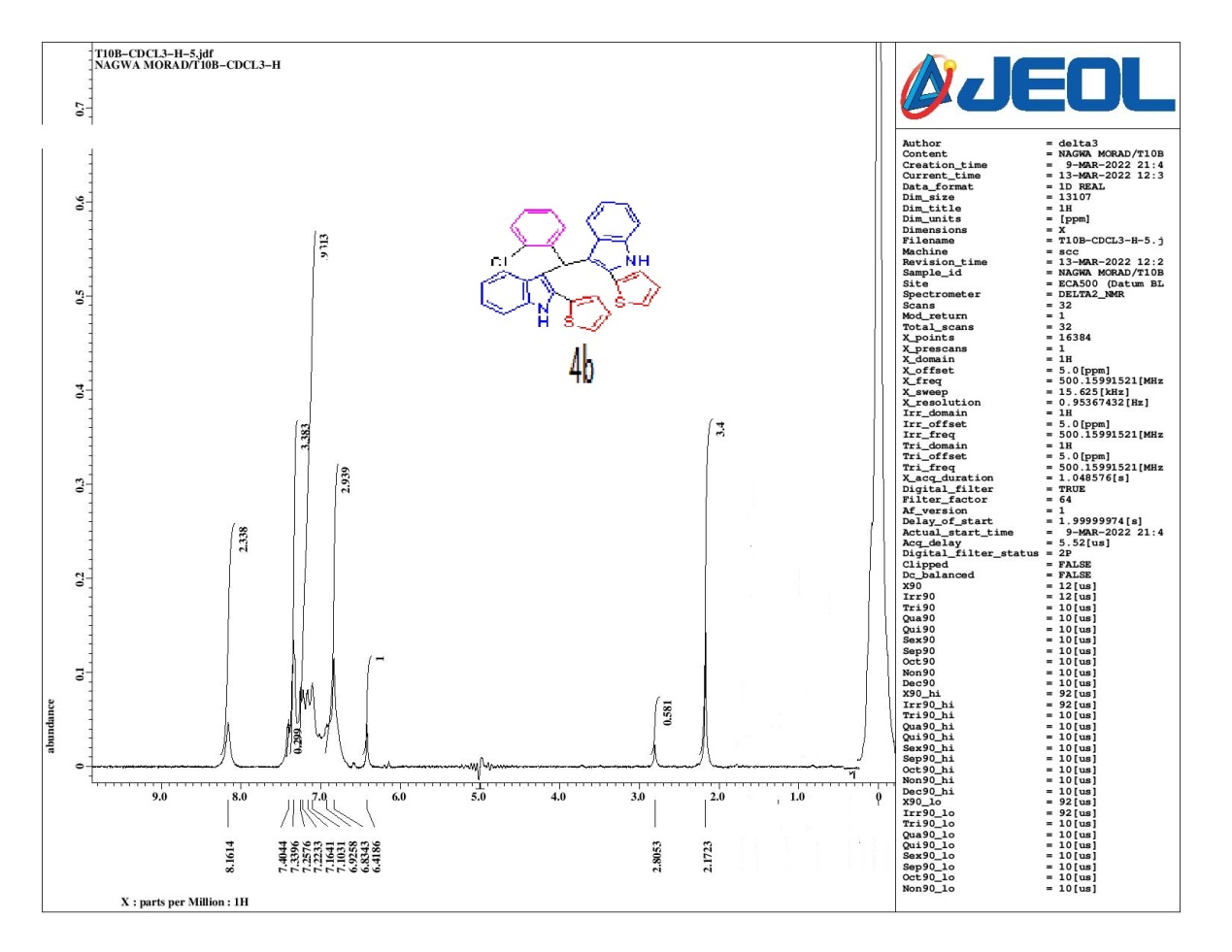


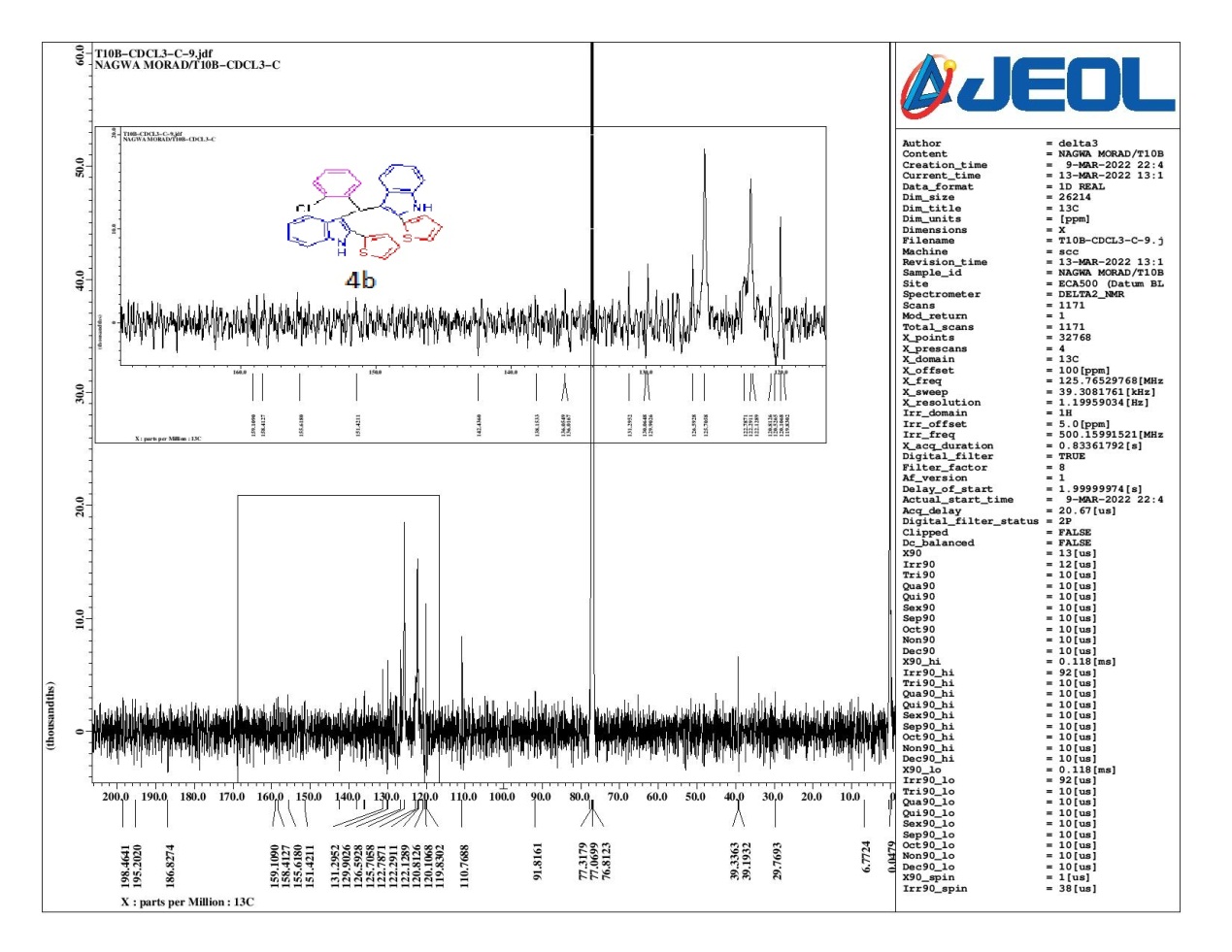


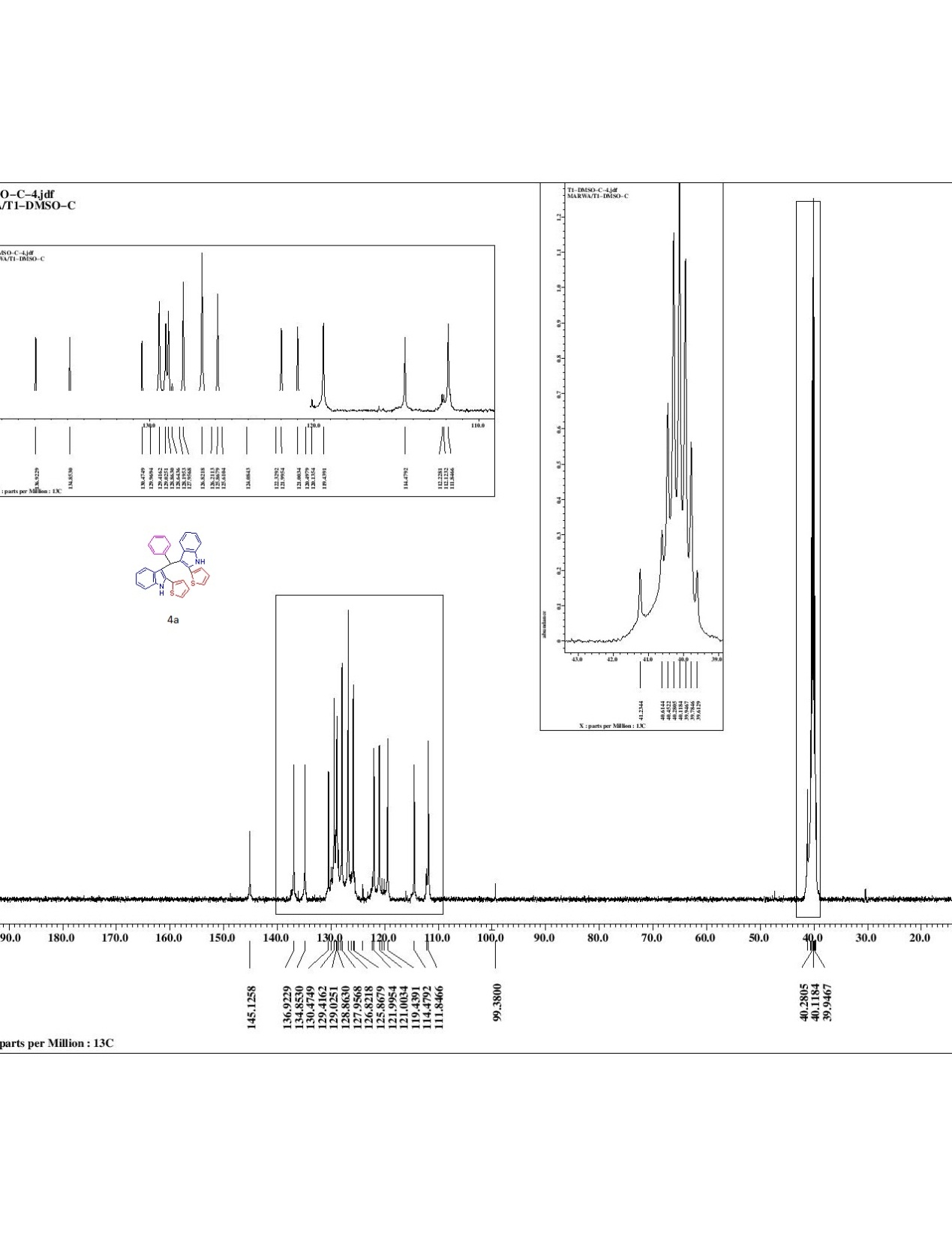


IR , ^1^H NMR, D2O and^13^C NMR for compound 4C


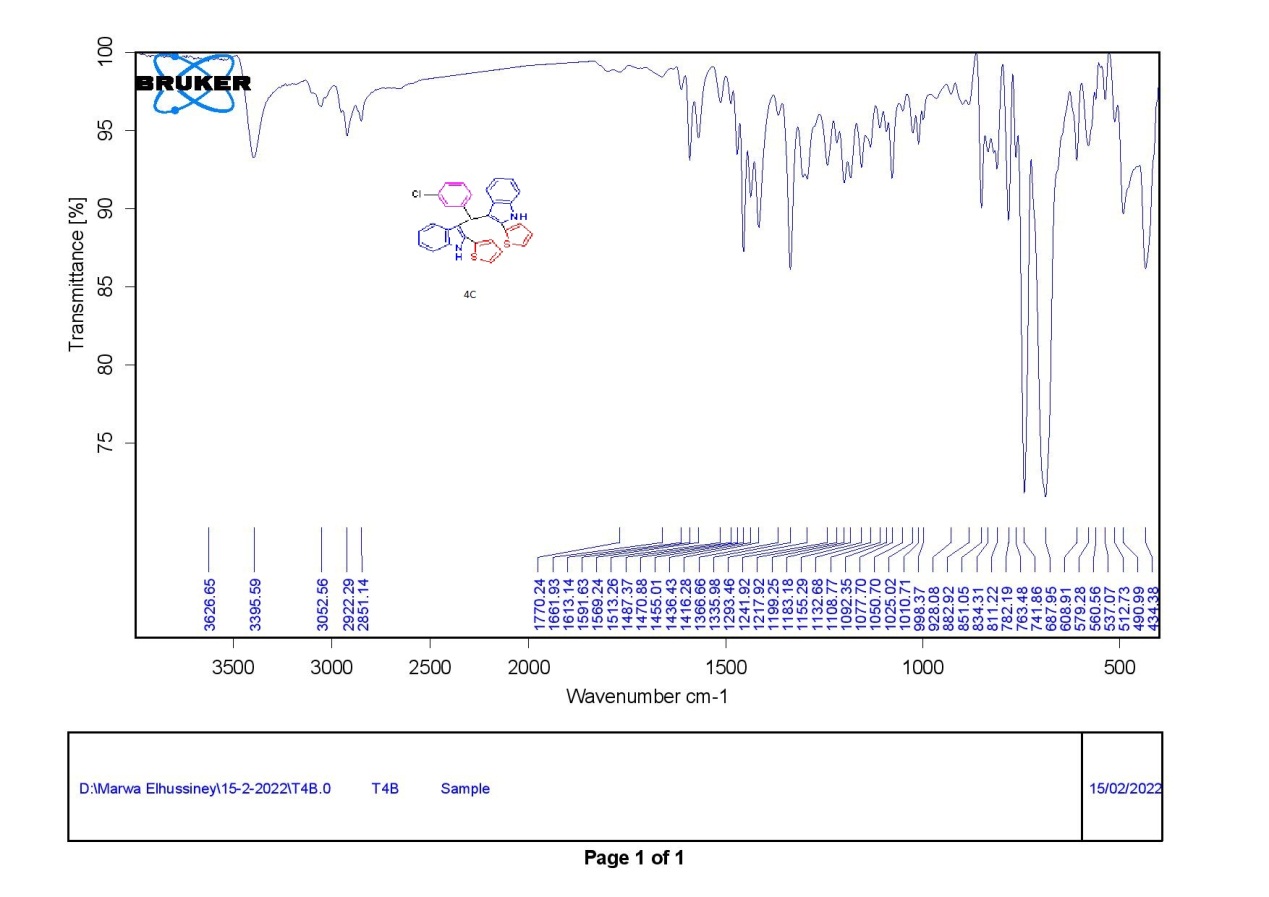


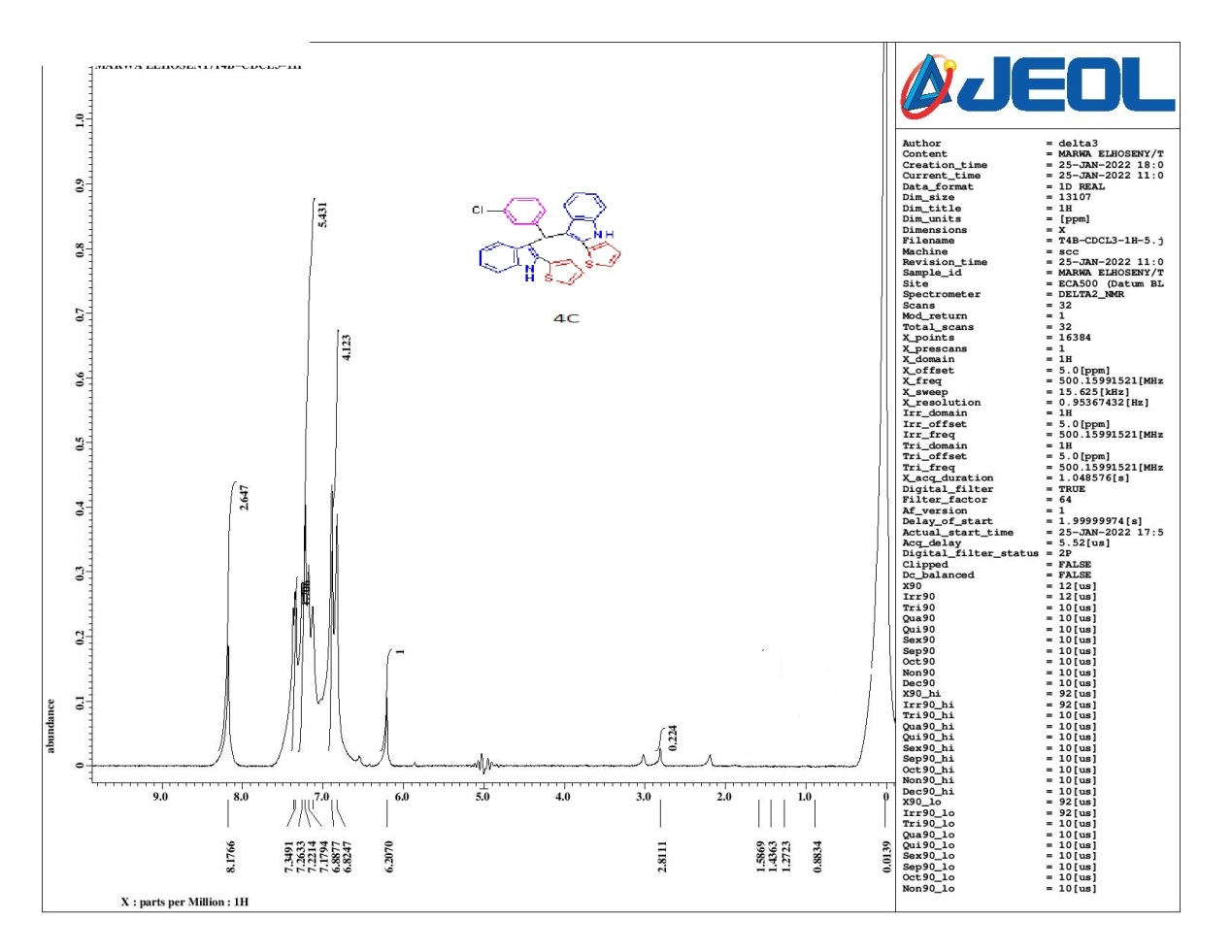


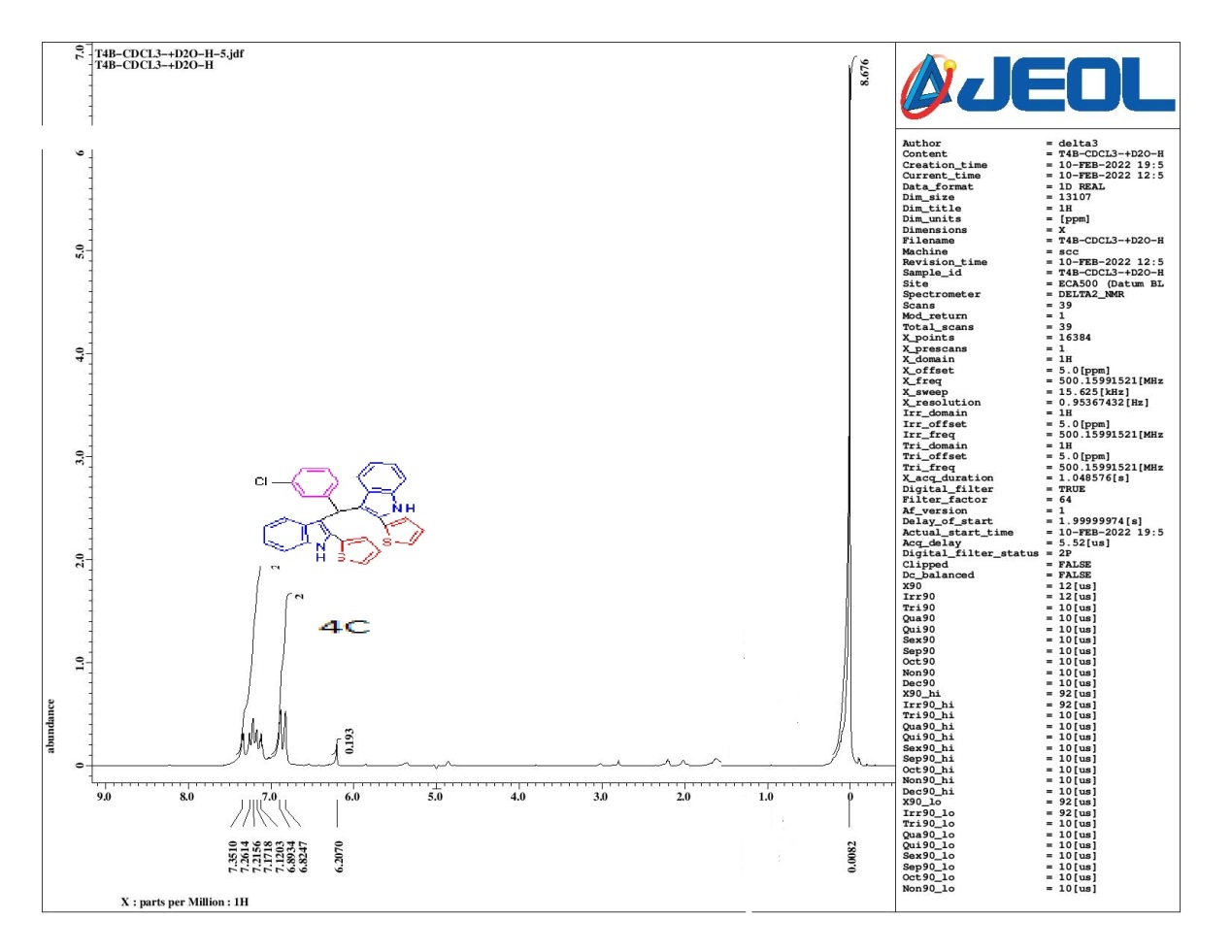


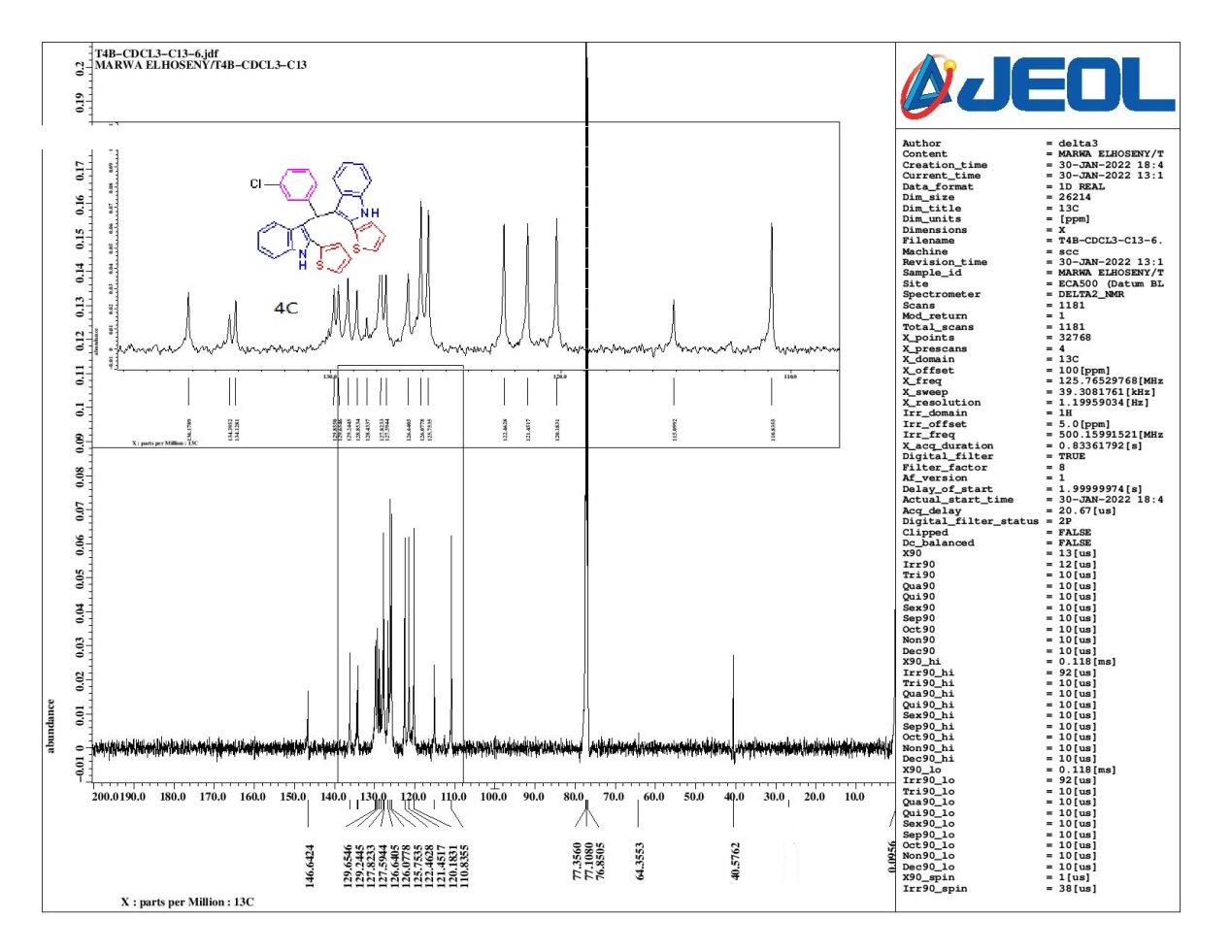


IR , ^1^H NMR and^13^C NMR for compound 4d


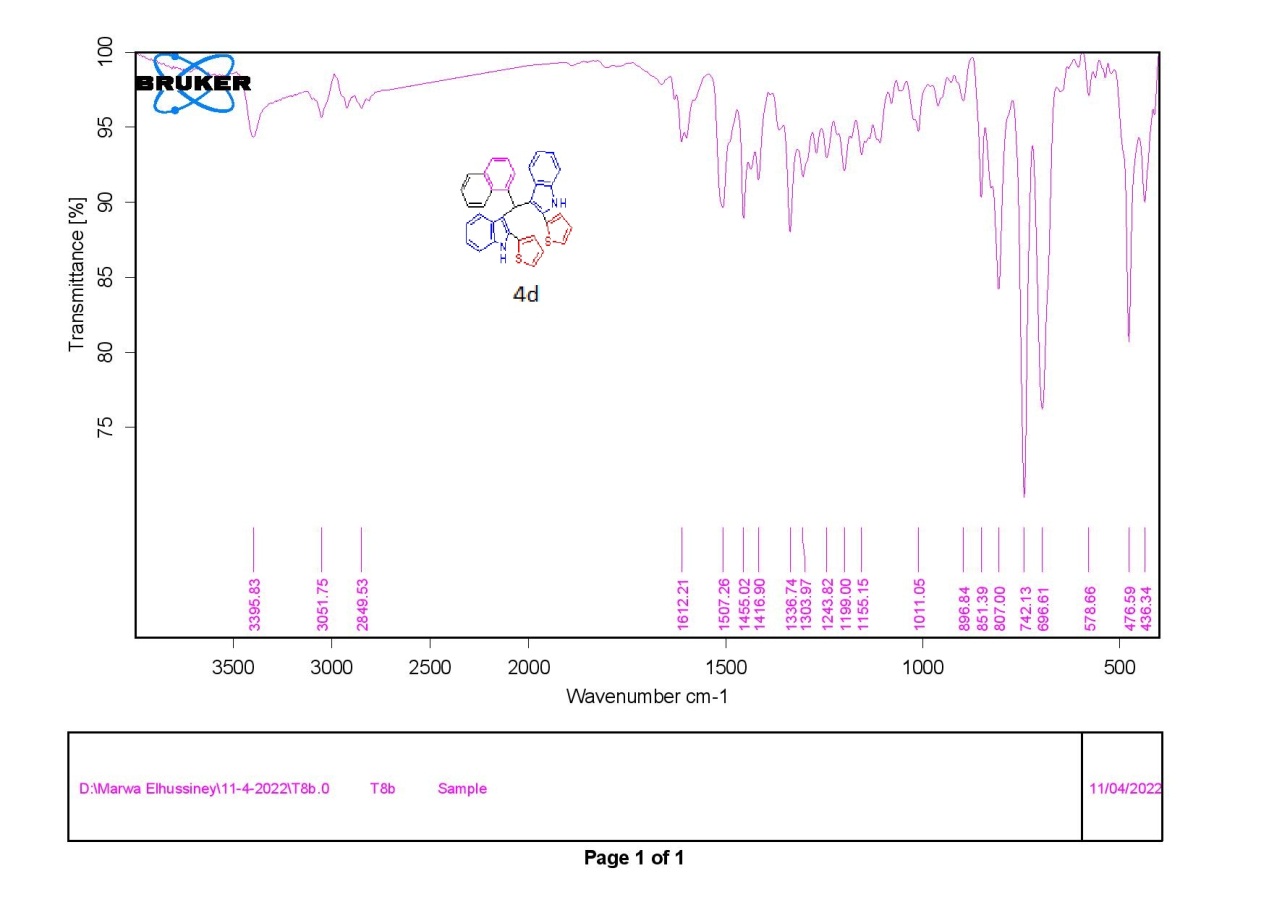

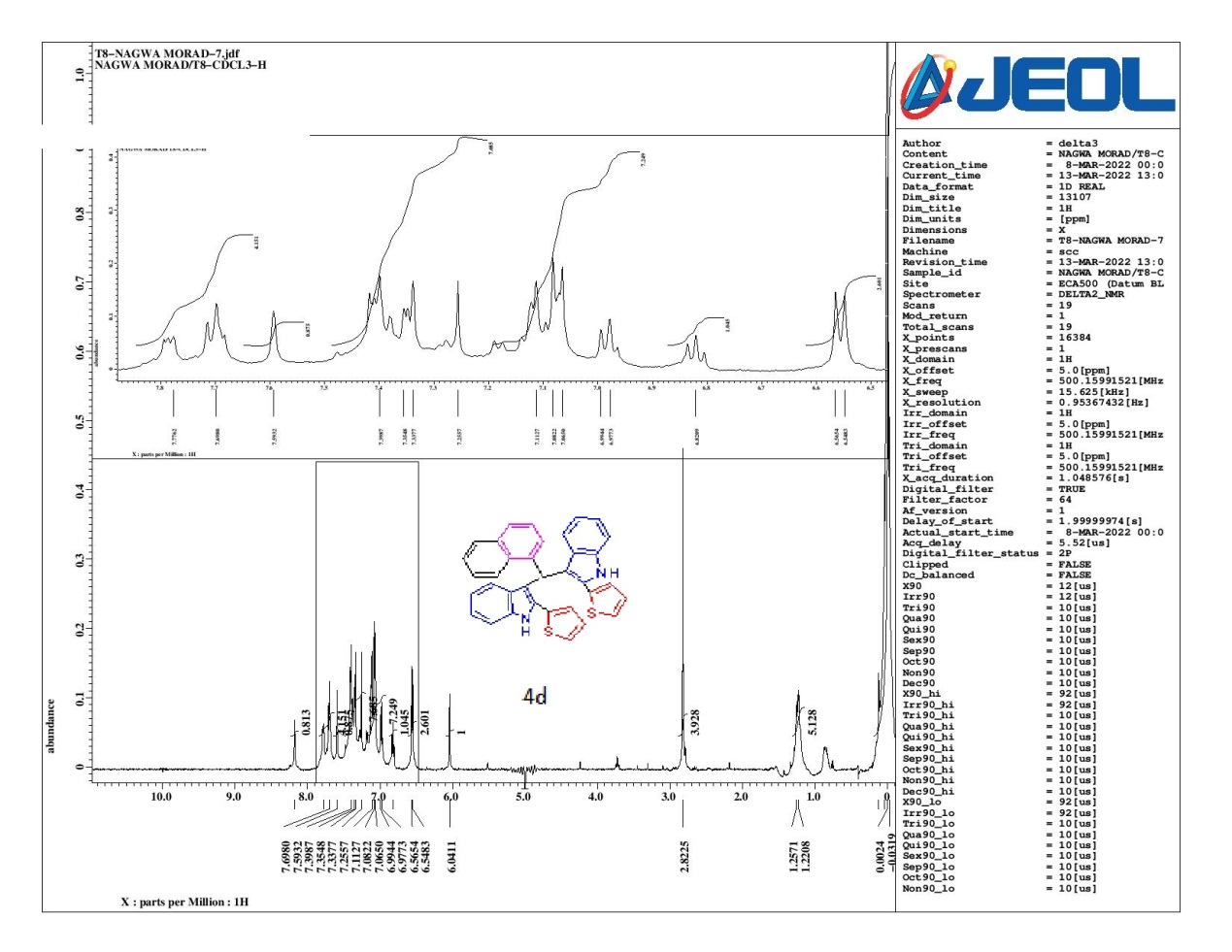


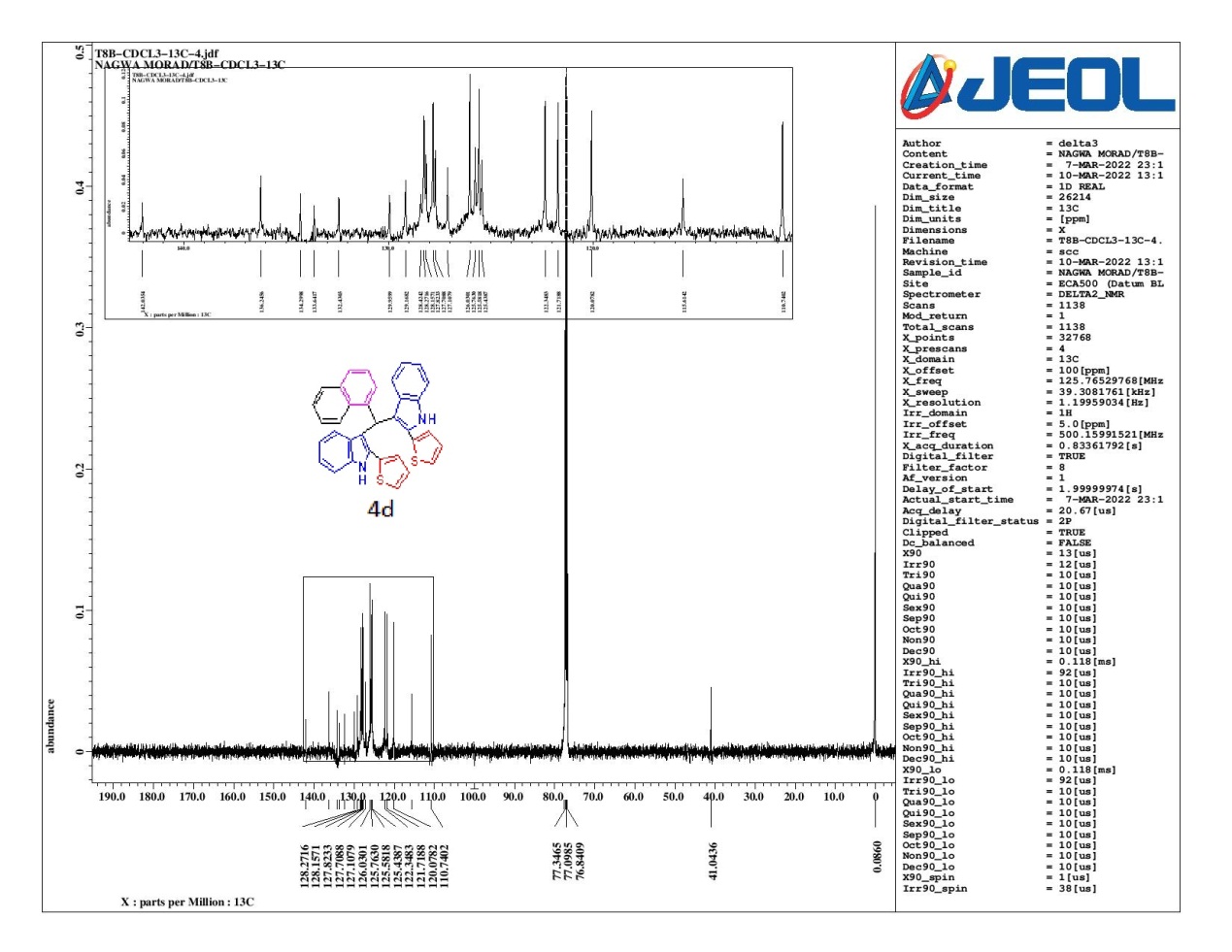


IR , ^1^H NMR and^13^C NMR for compound 4e


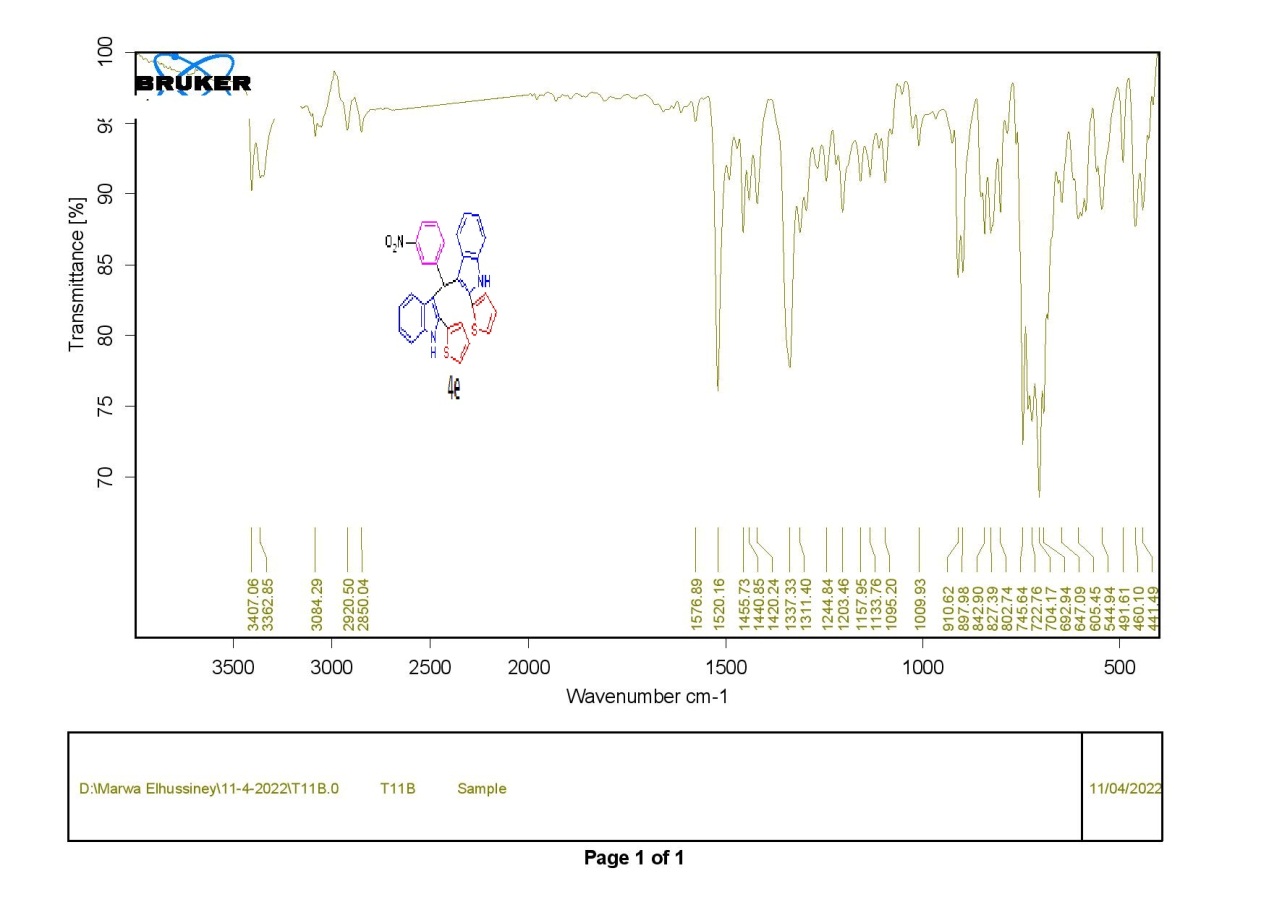


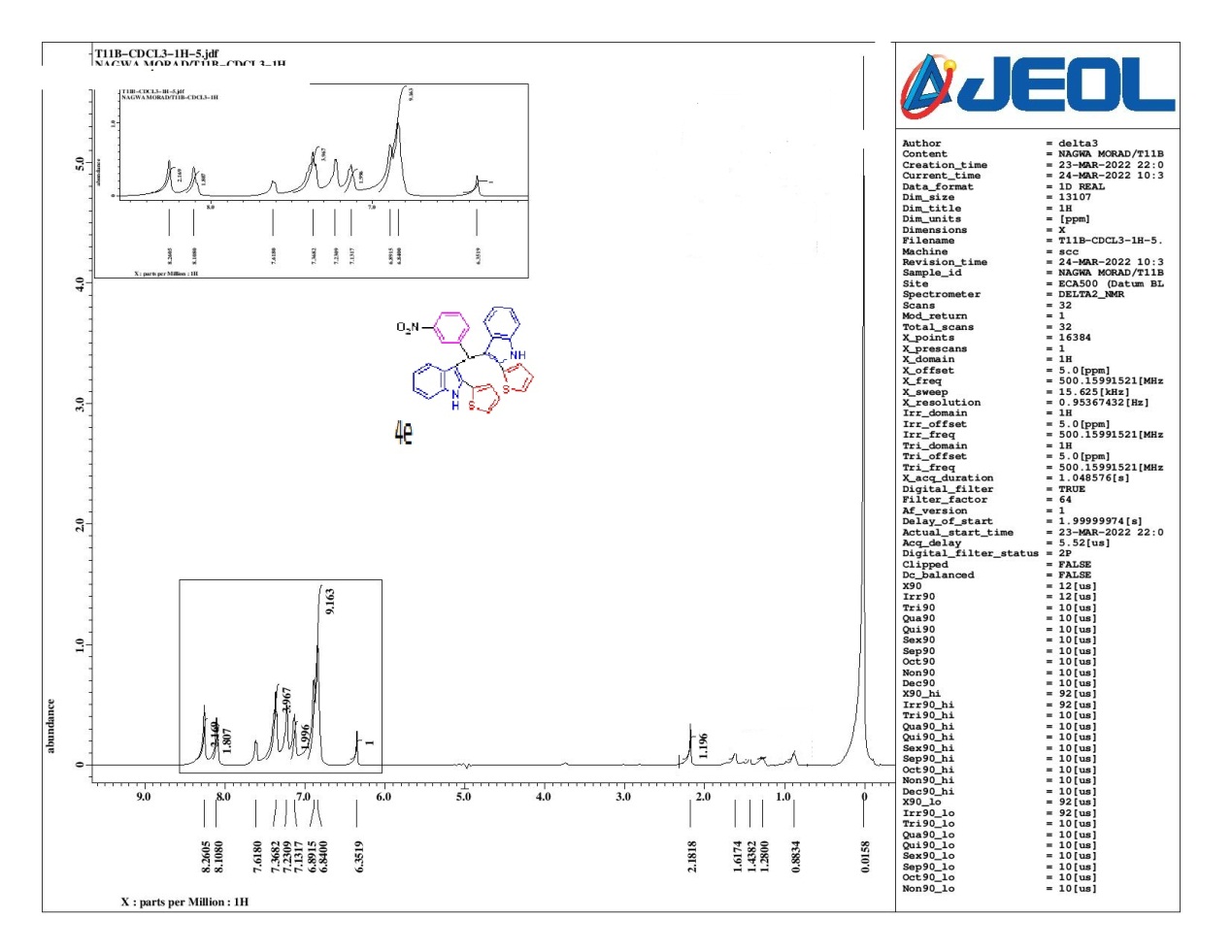


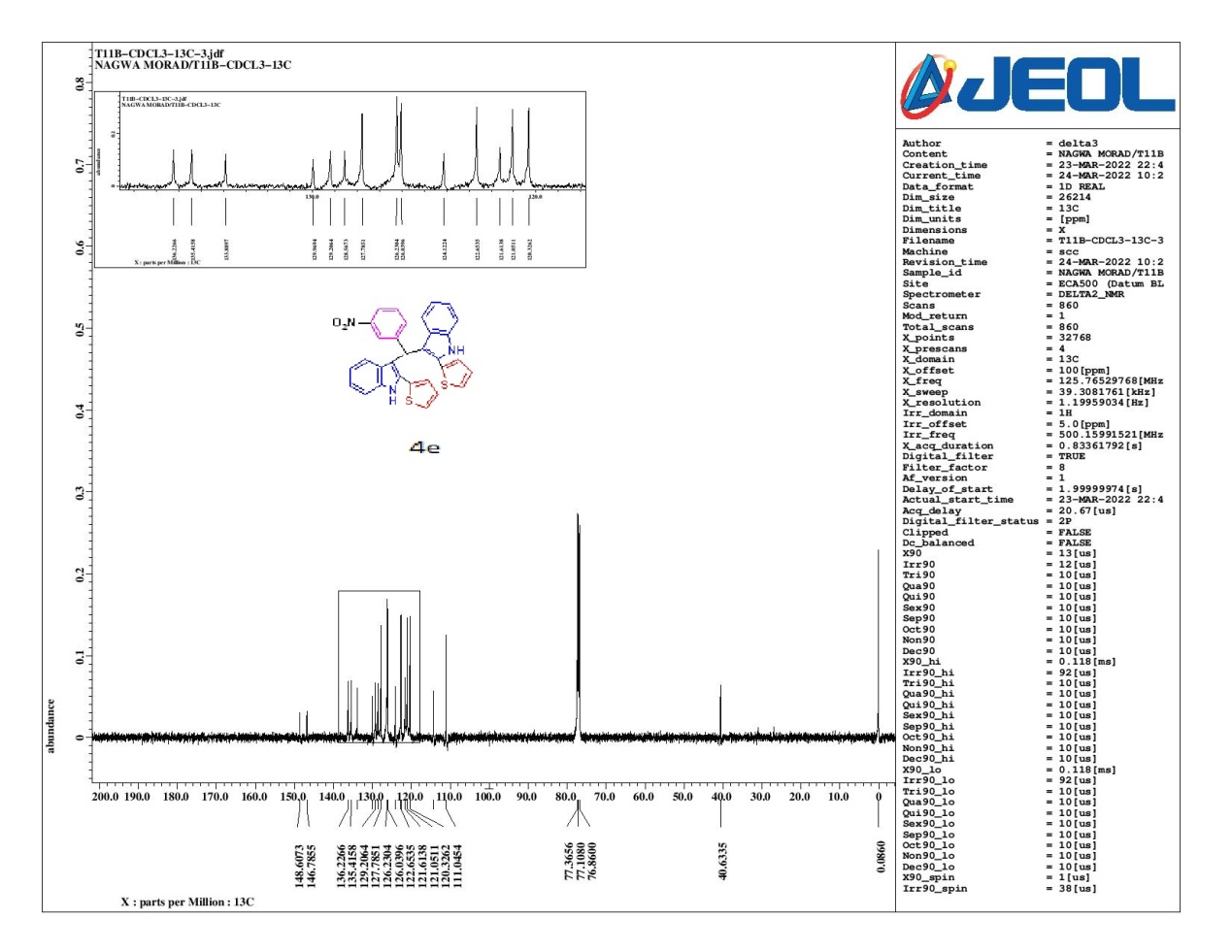


IR , ^1^H NMR and^13^C NMR for compound 4F


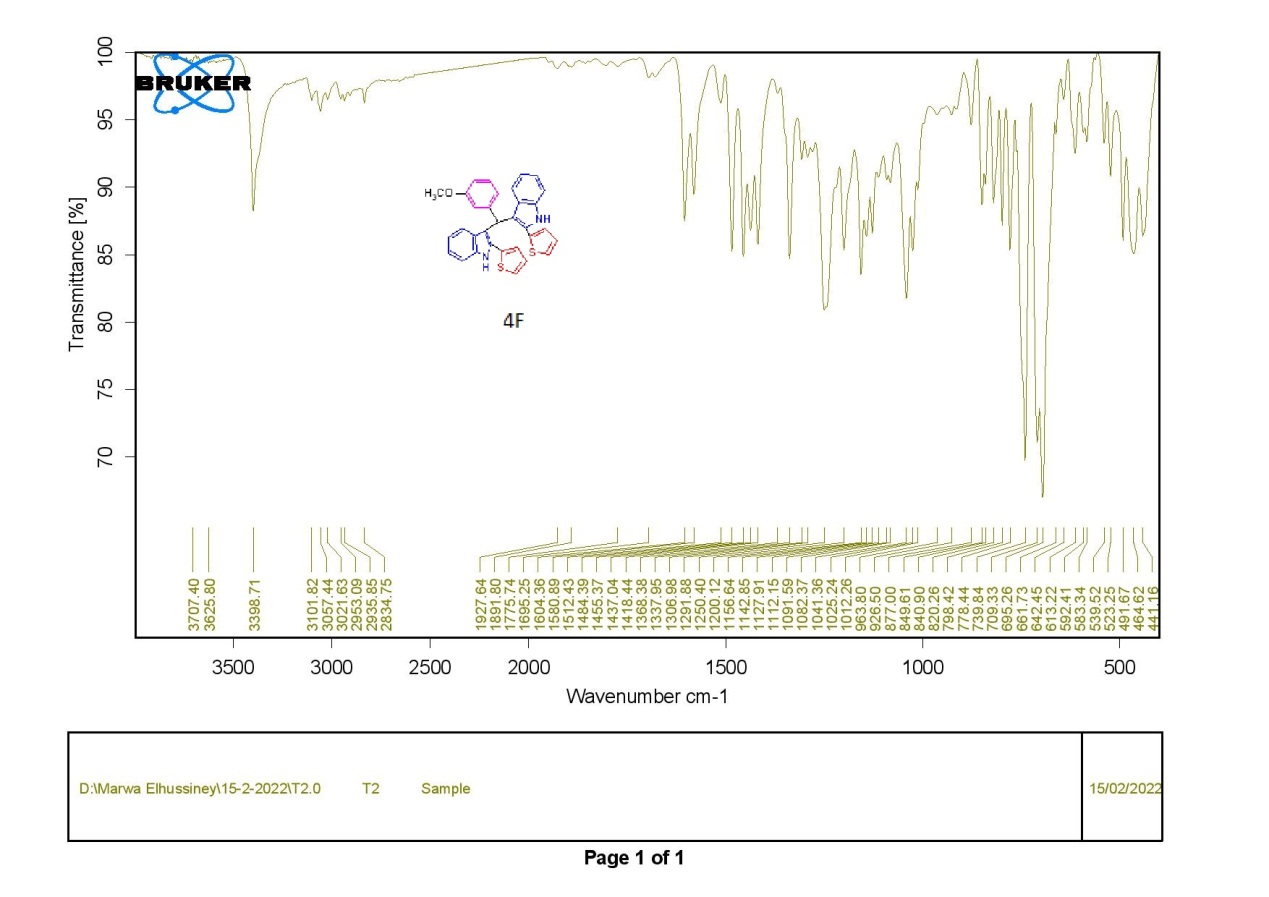


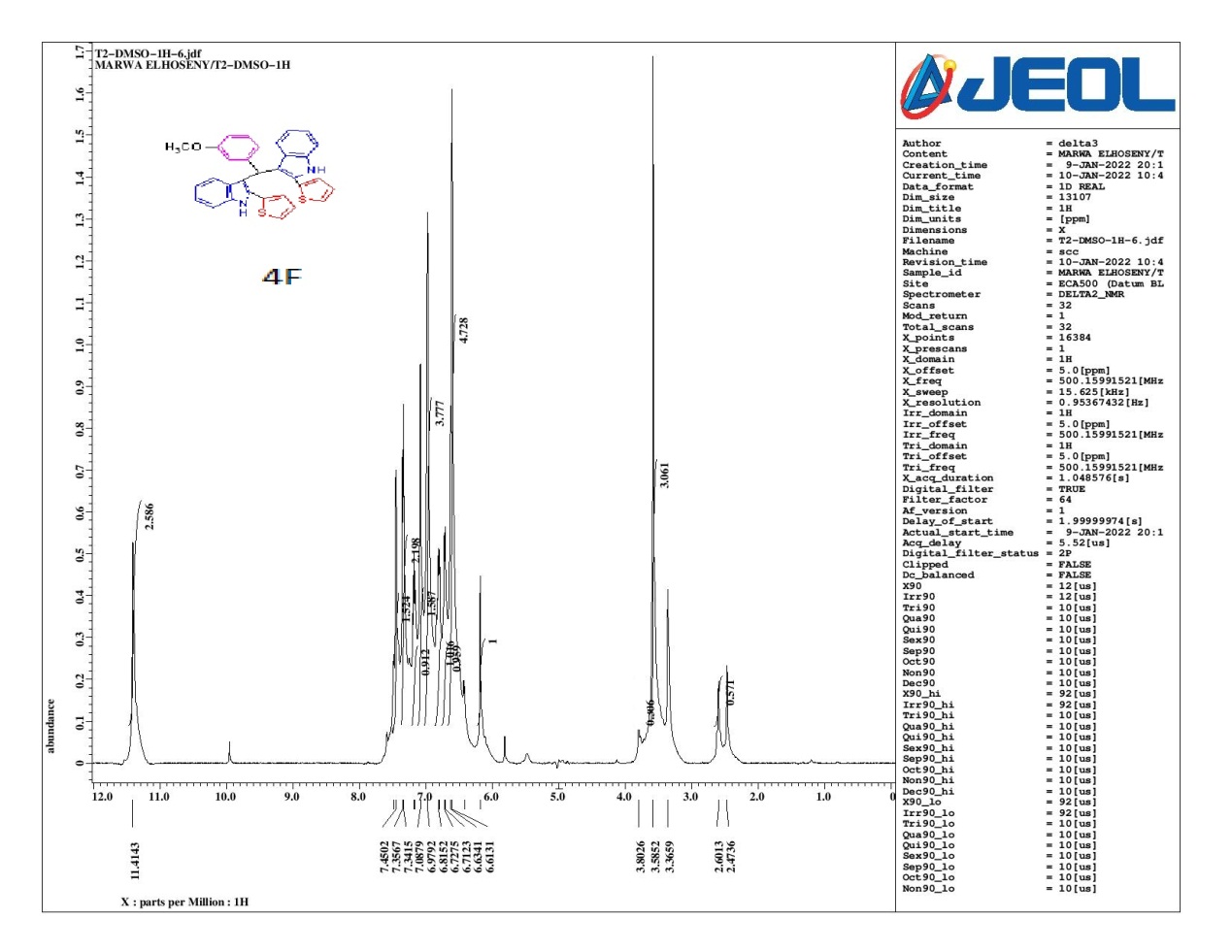


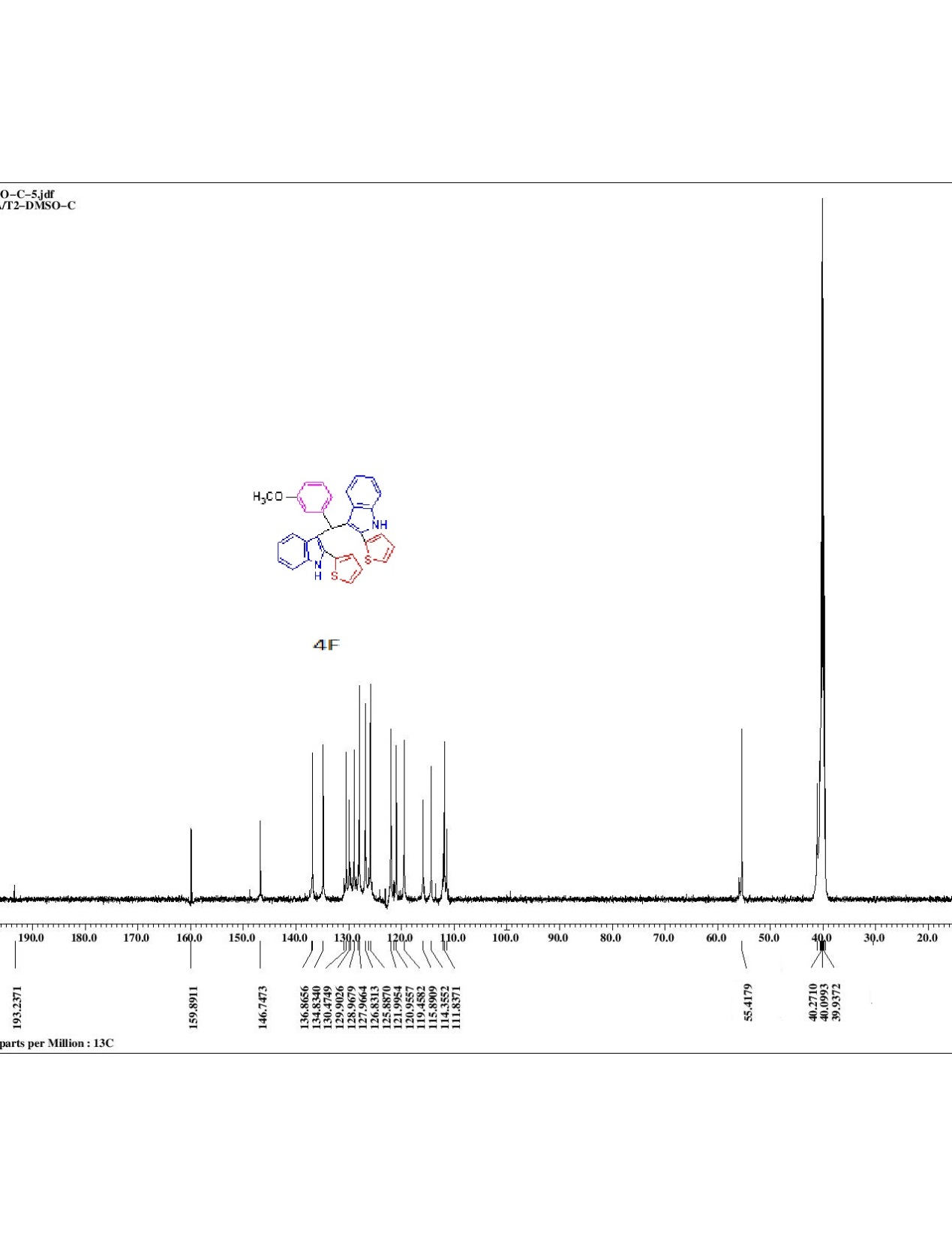


IR , ^1^H NMR and^13^C NMR for compound 4g


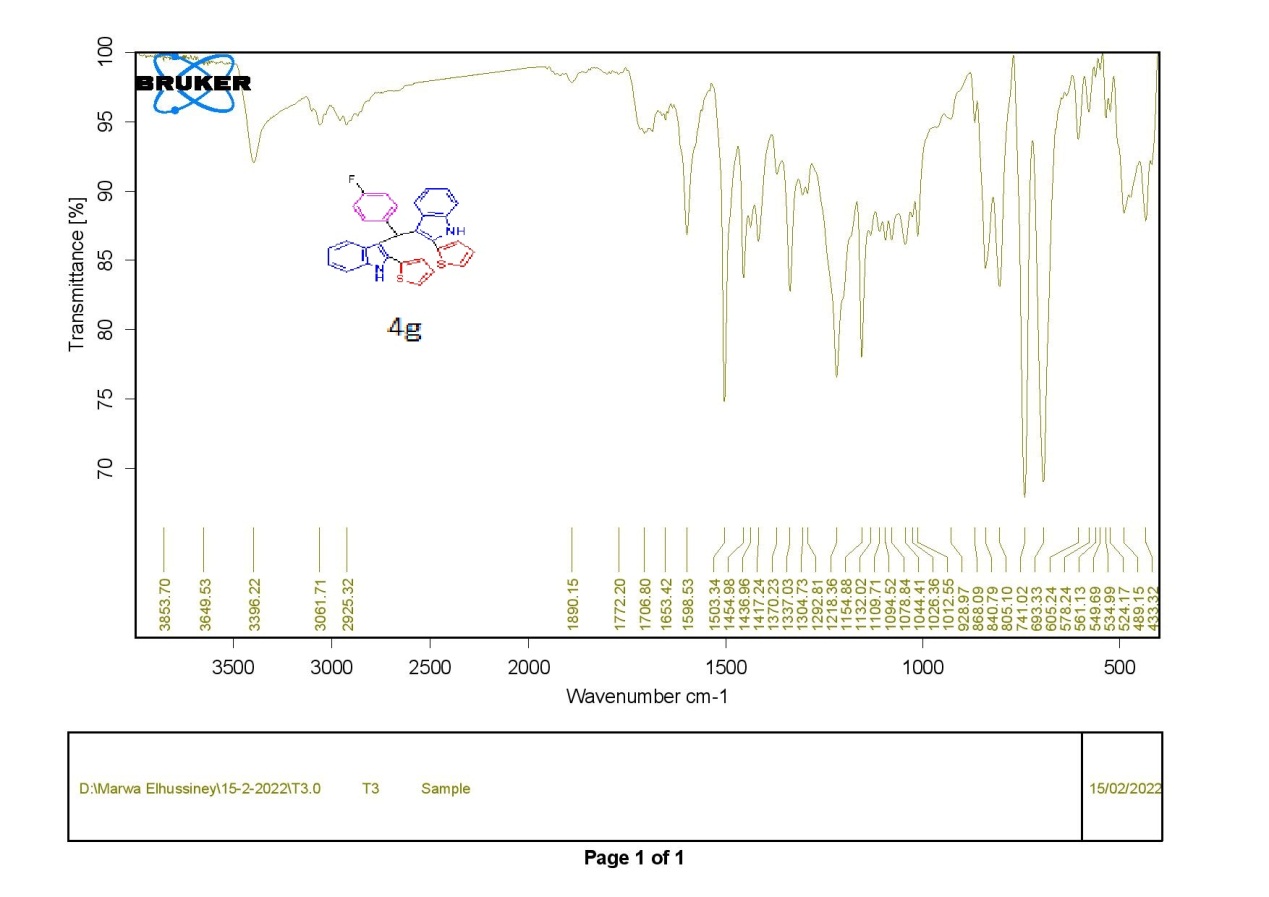


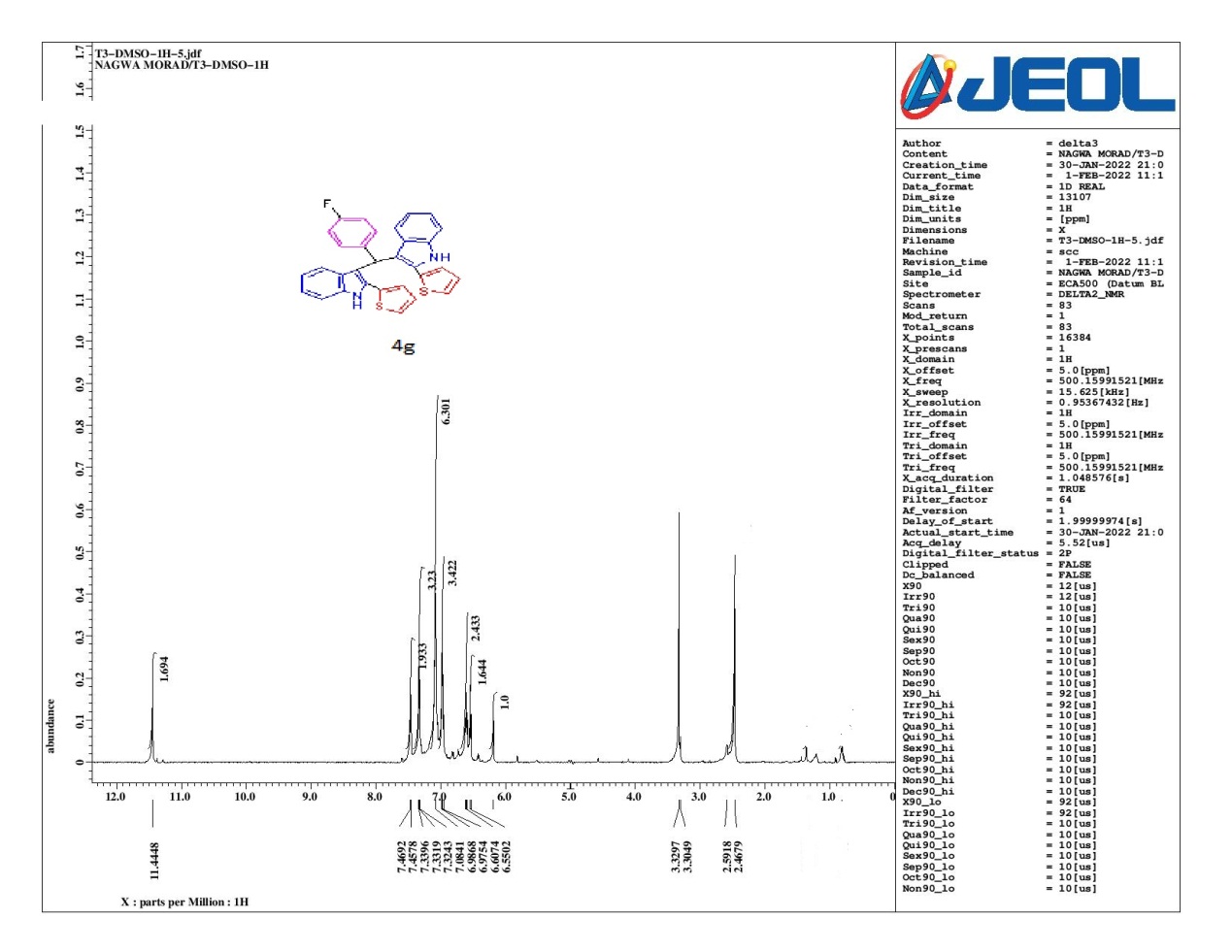


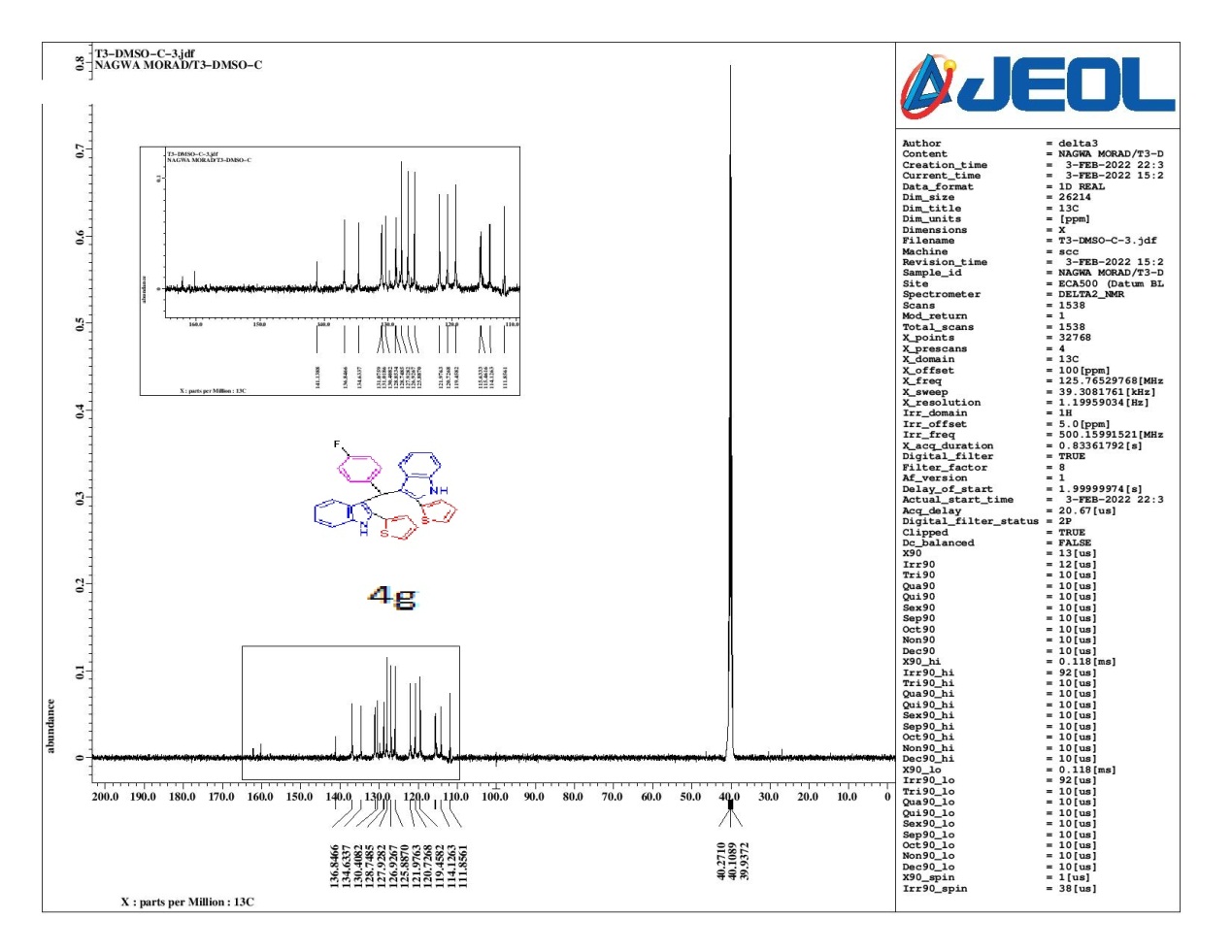


IR , ^1^H NMR and^13^C NMR for compound 4h


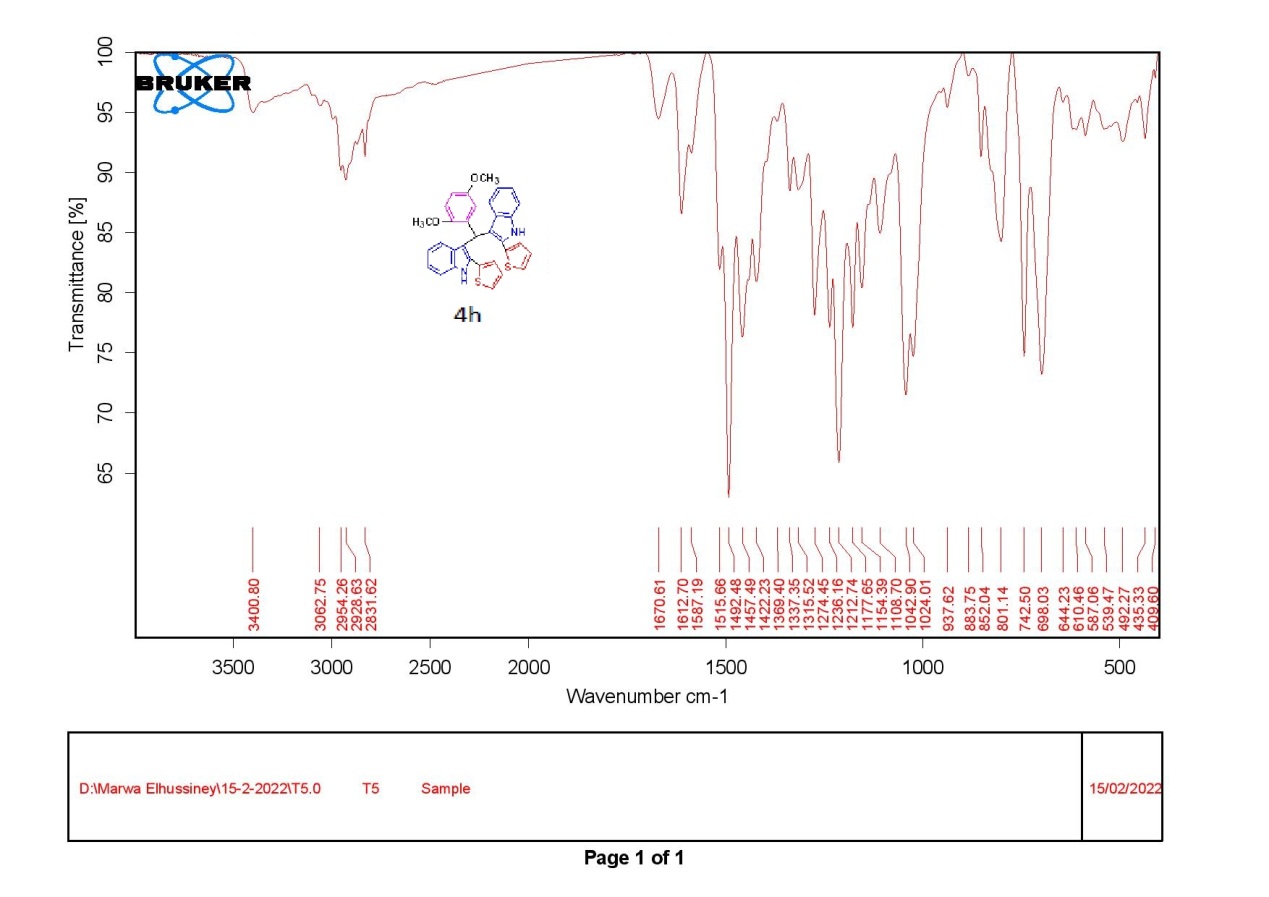


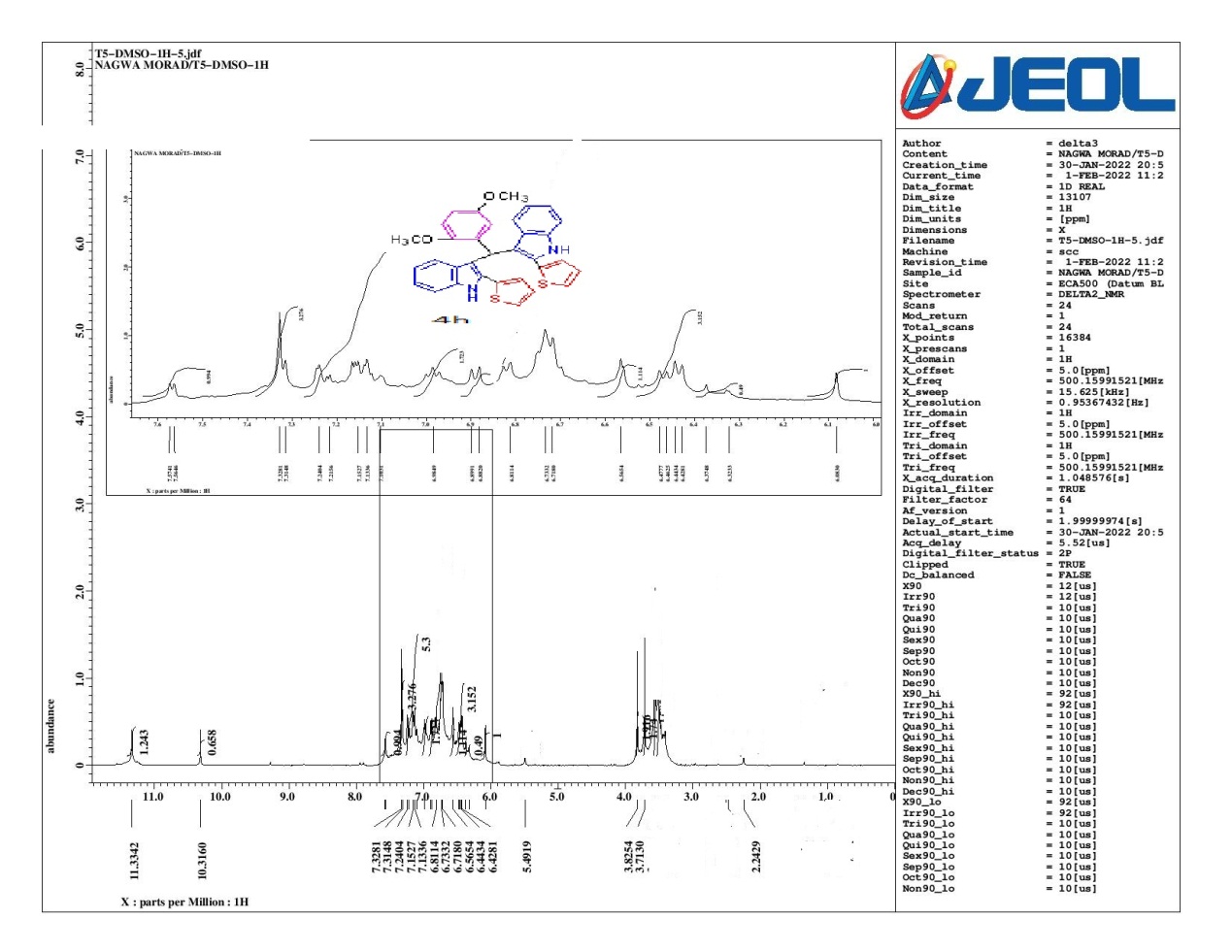


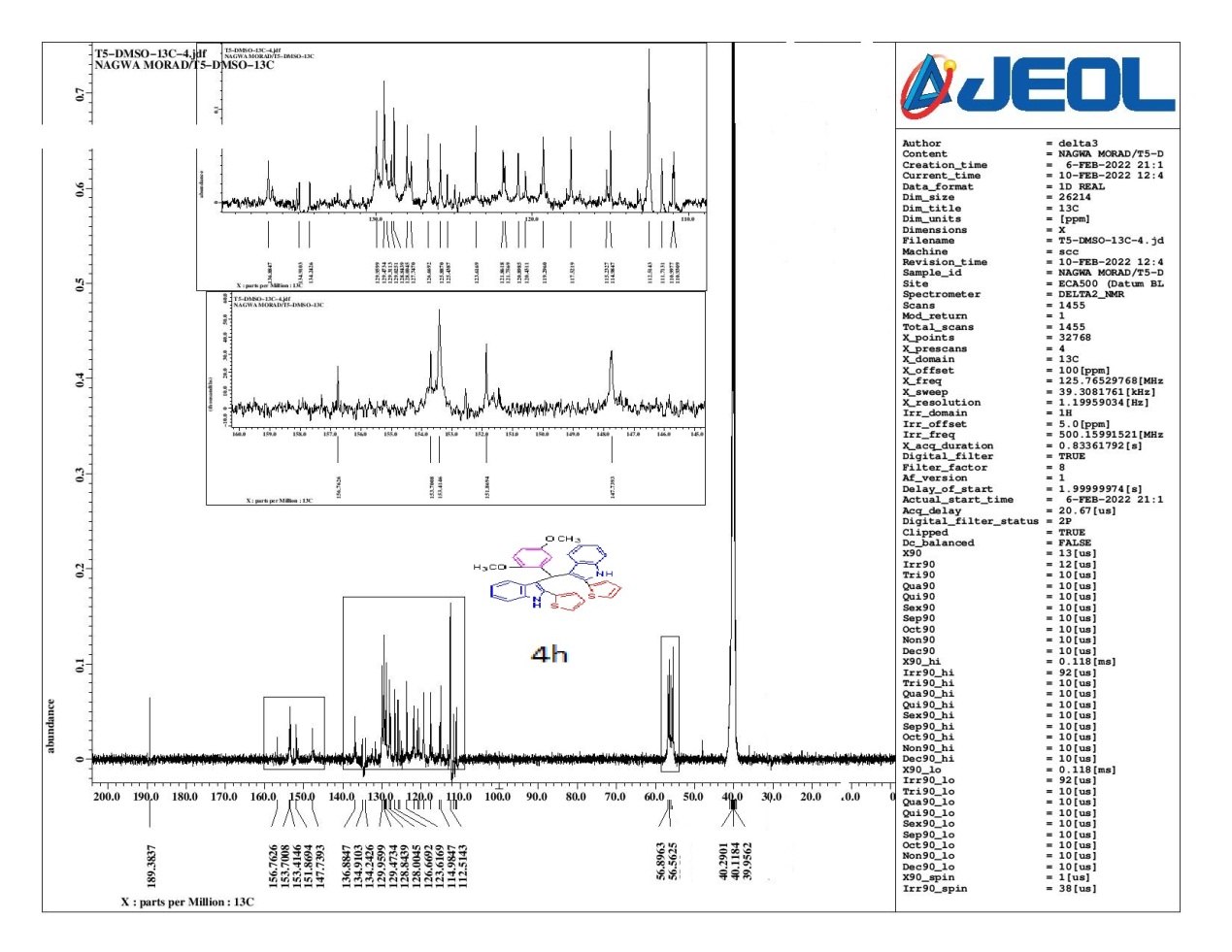


^1^H NMR for compound 4i


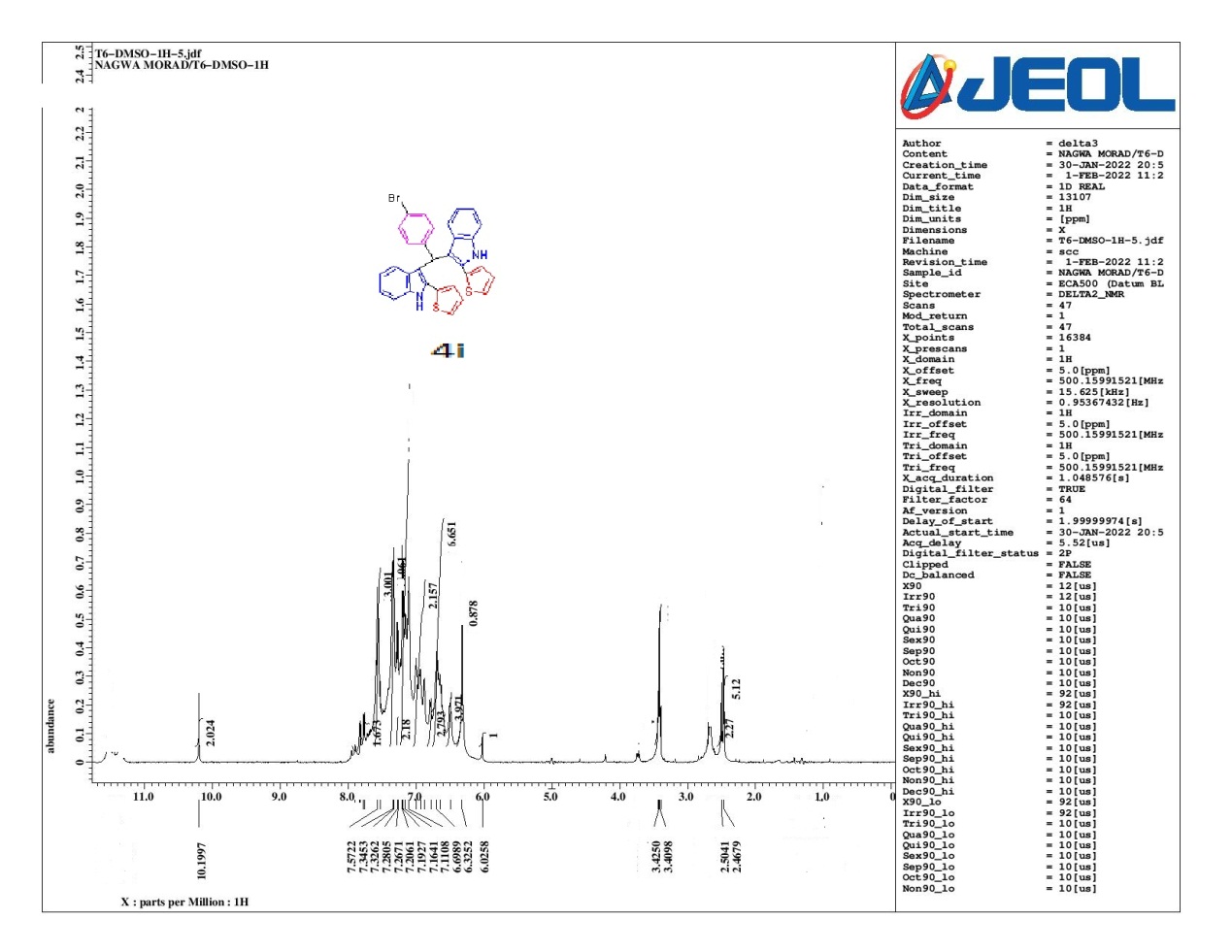


IR , ^1^H NMR and^13^C NMR for compound 4j


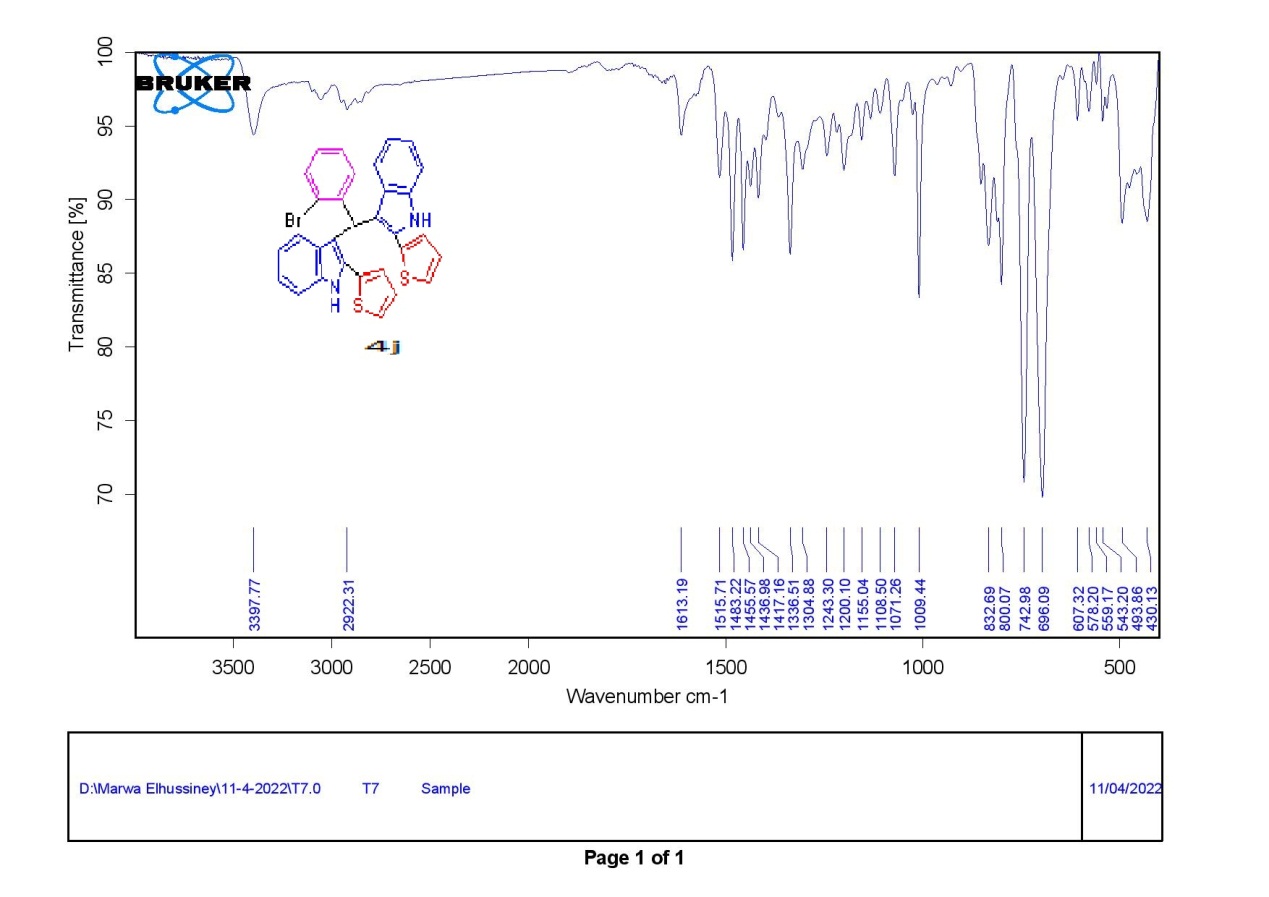


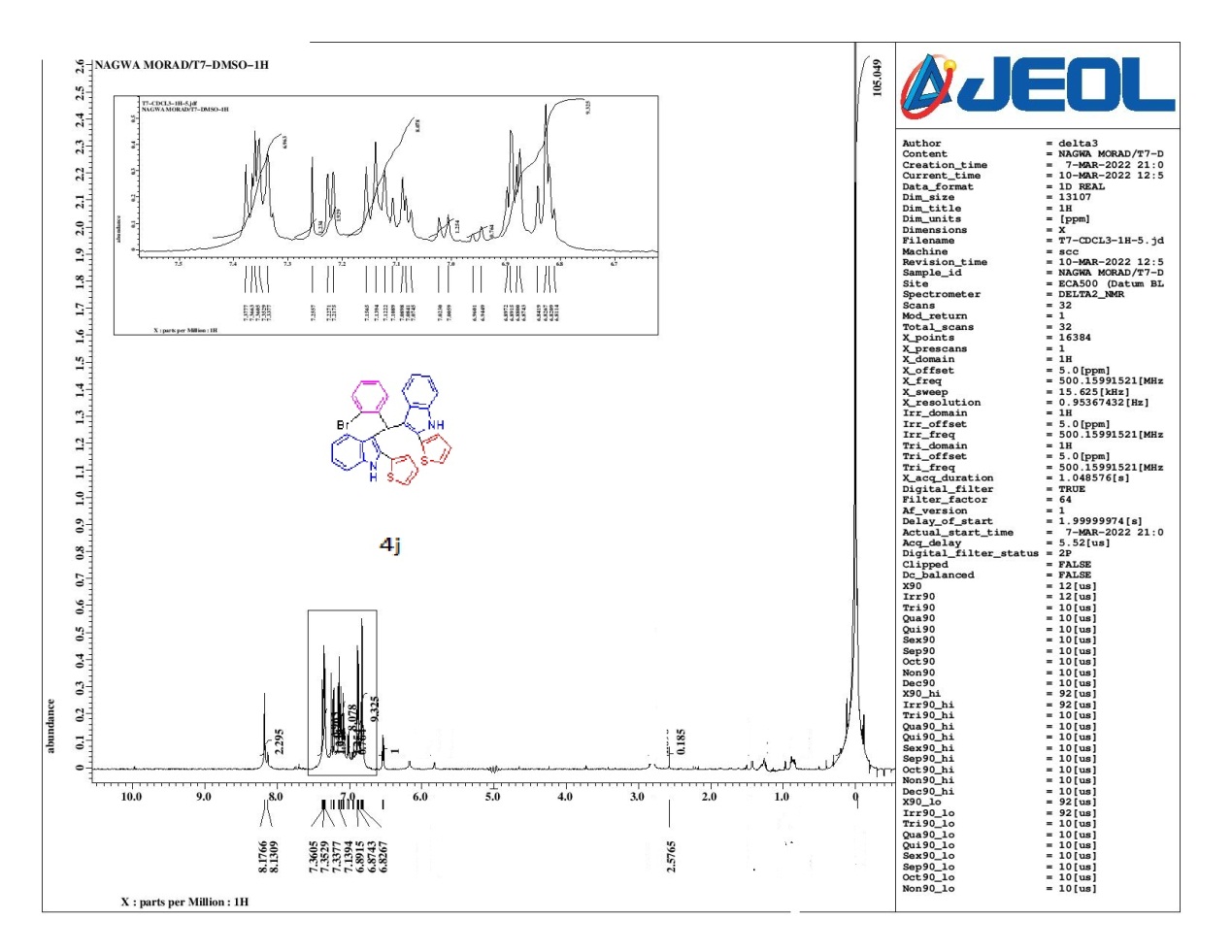


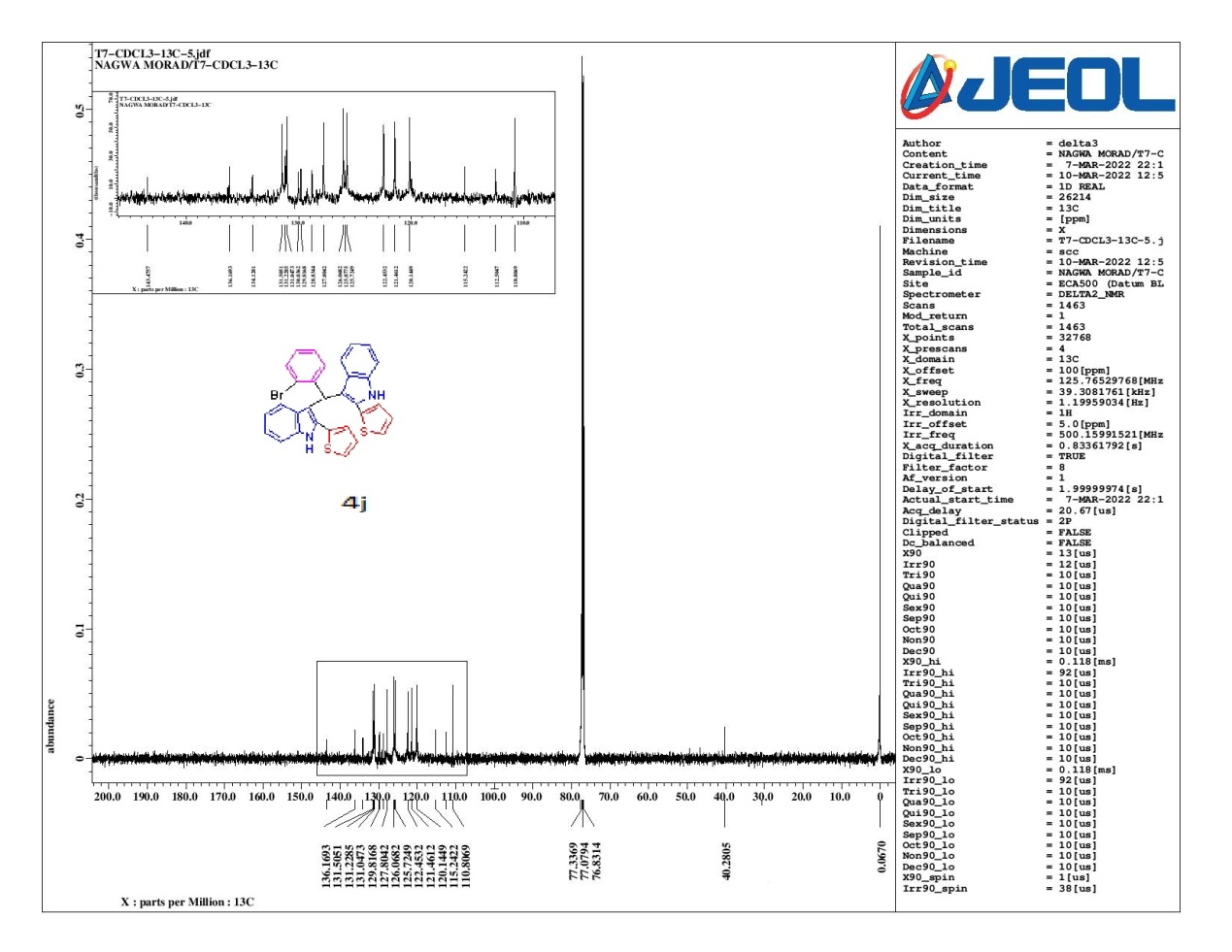


IR , ^1^H NMR and^13^C NMR for compound 4K


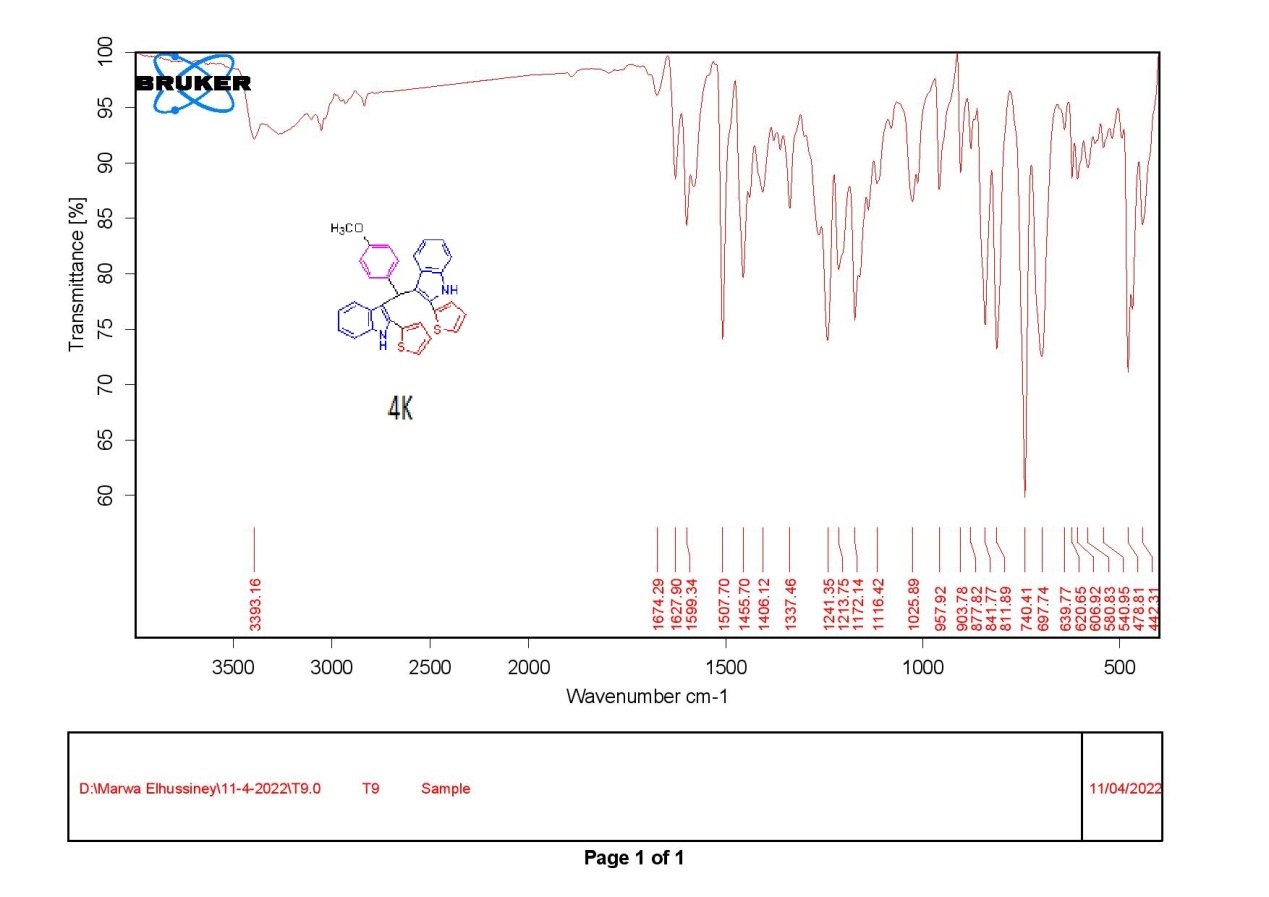


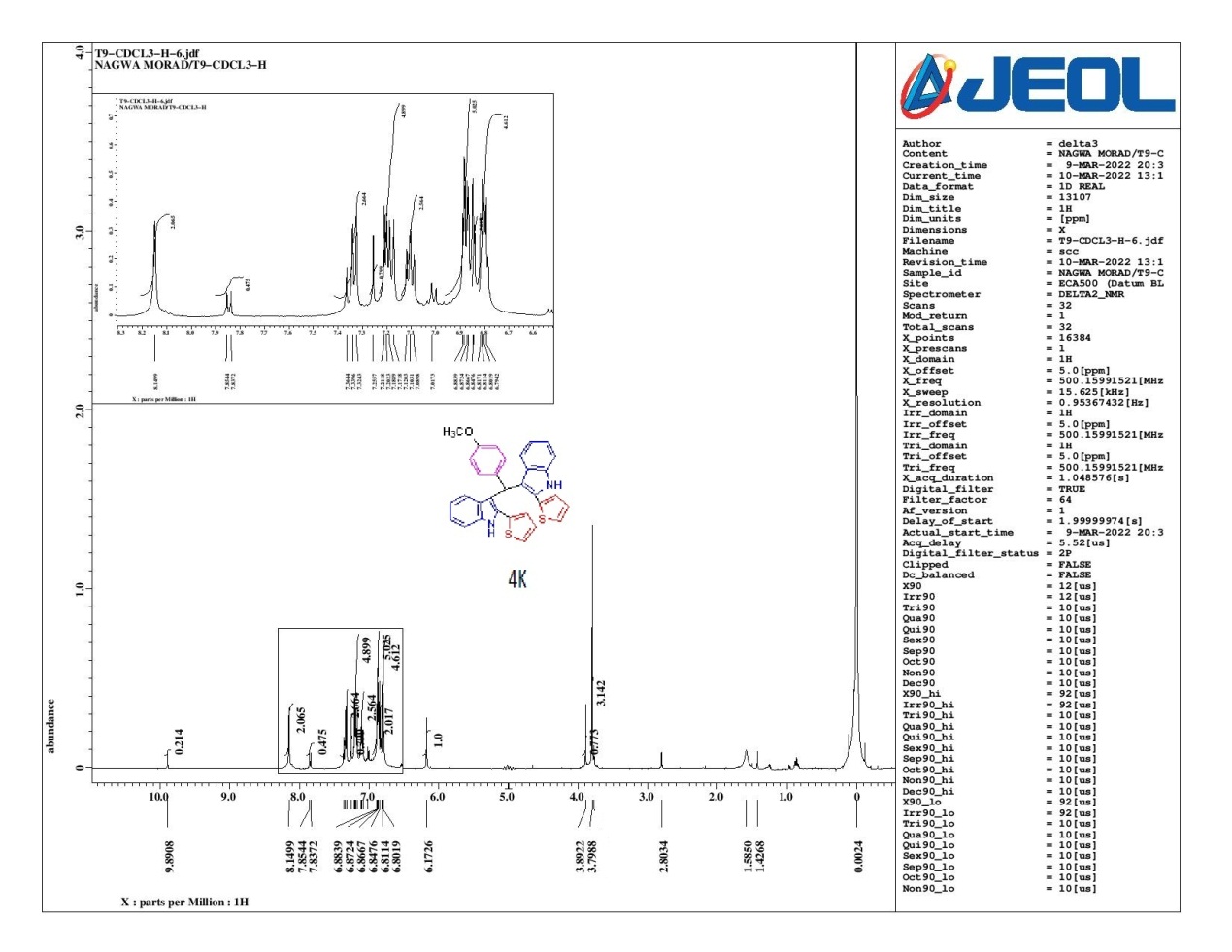


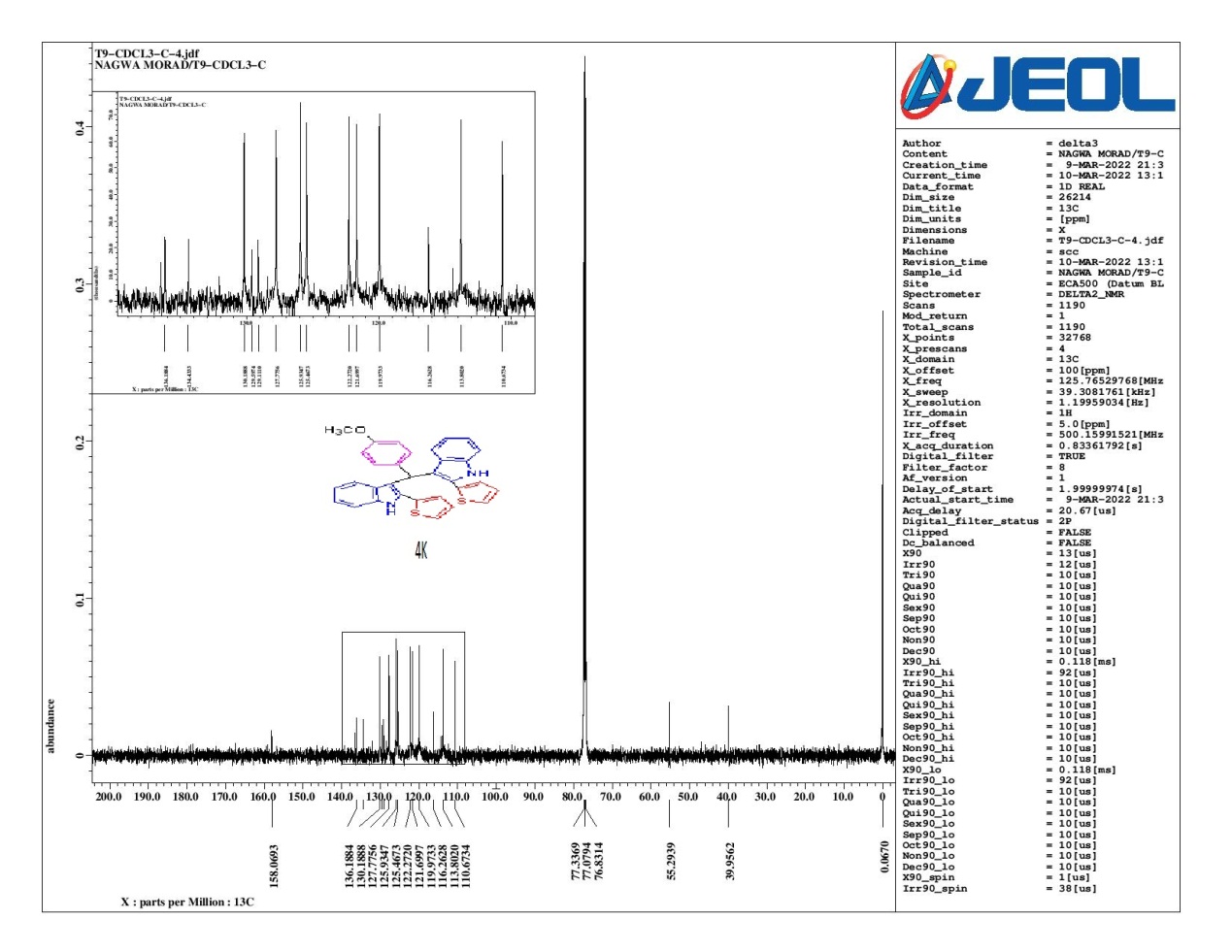


^1^H NMR for compound 5a


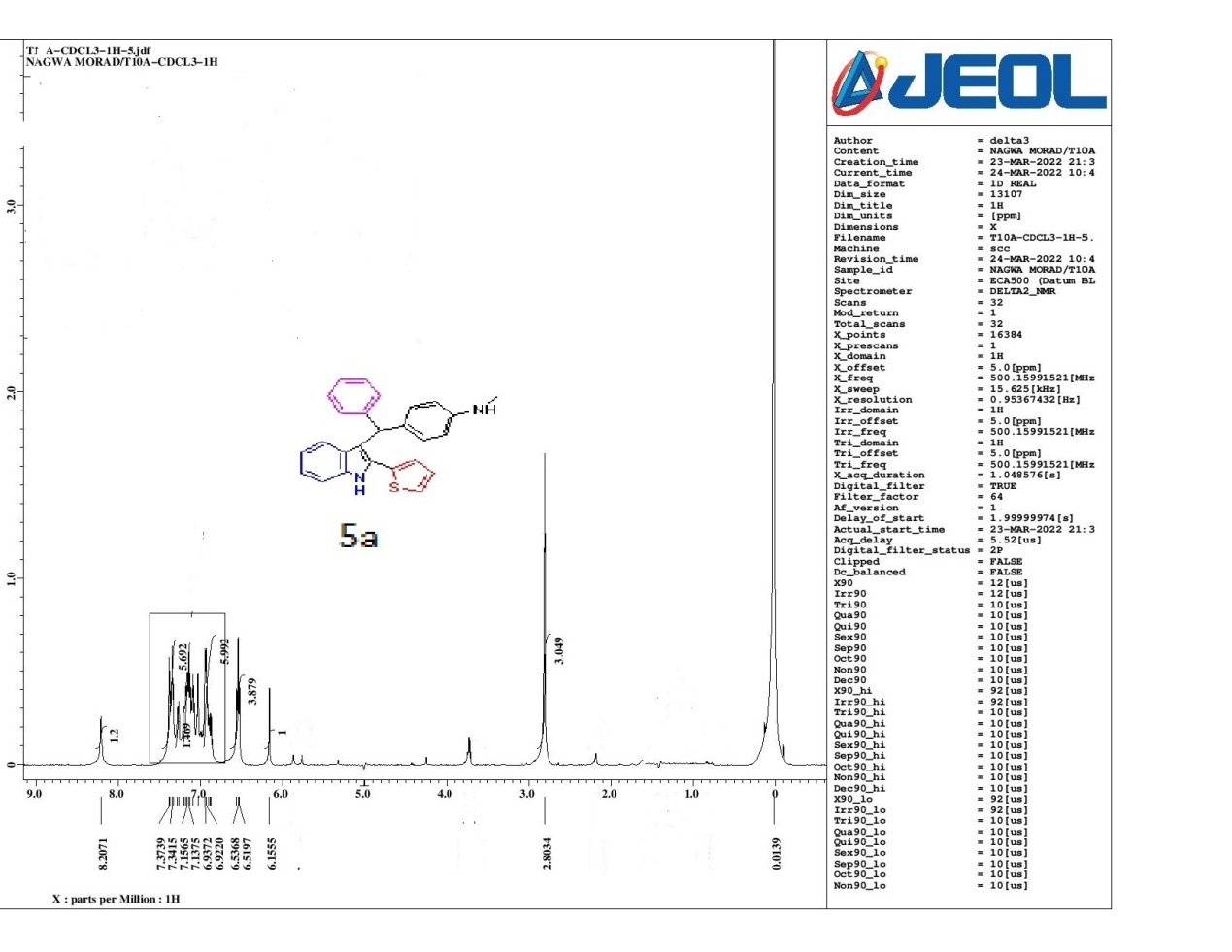


^13^C NMR for compound 5b


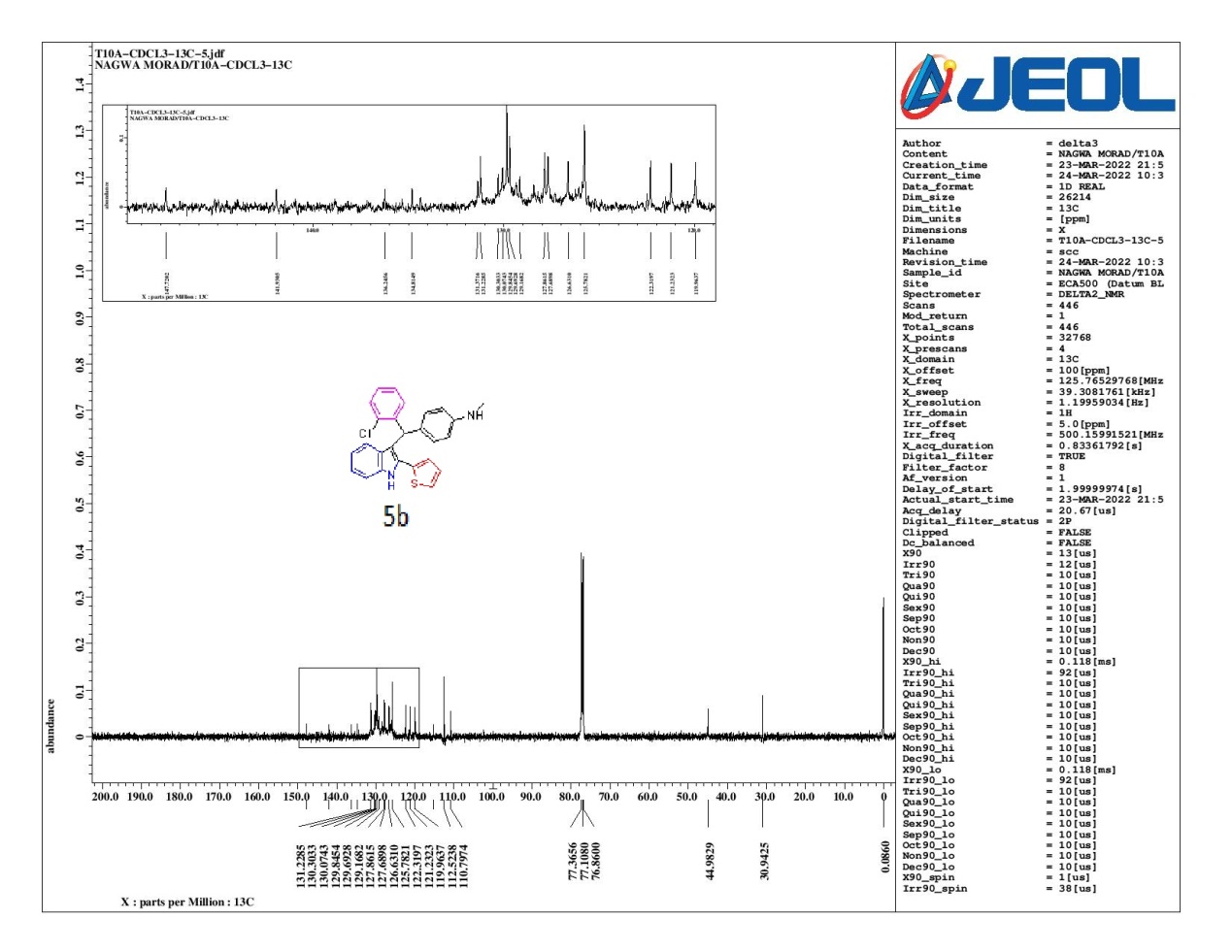


^1^H NMR for compound 5c


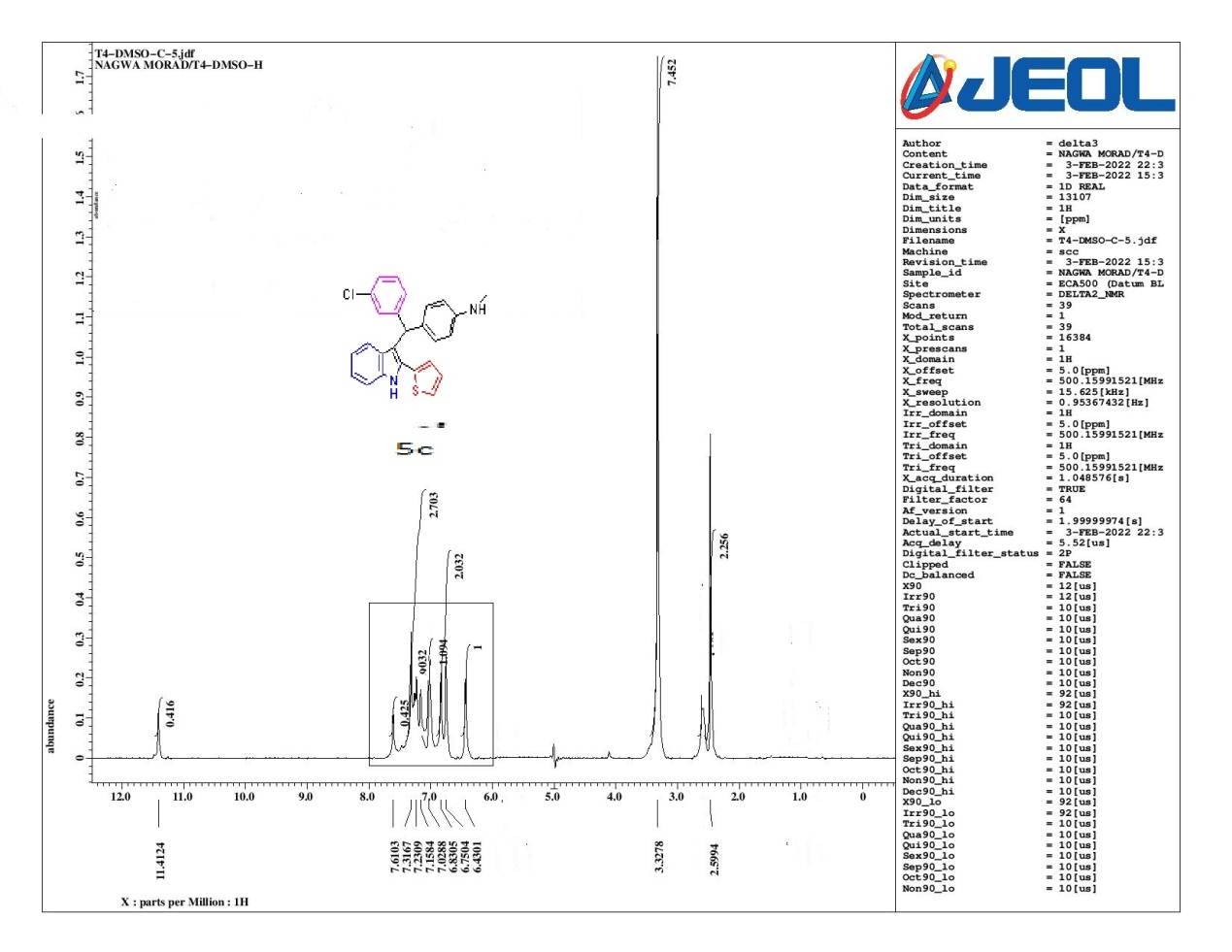


^1^H NMR and^13^C NMR for compound 5d


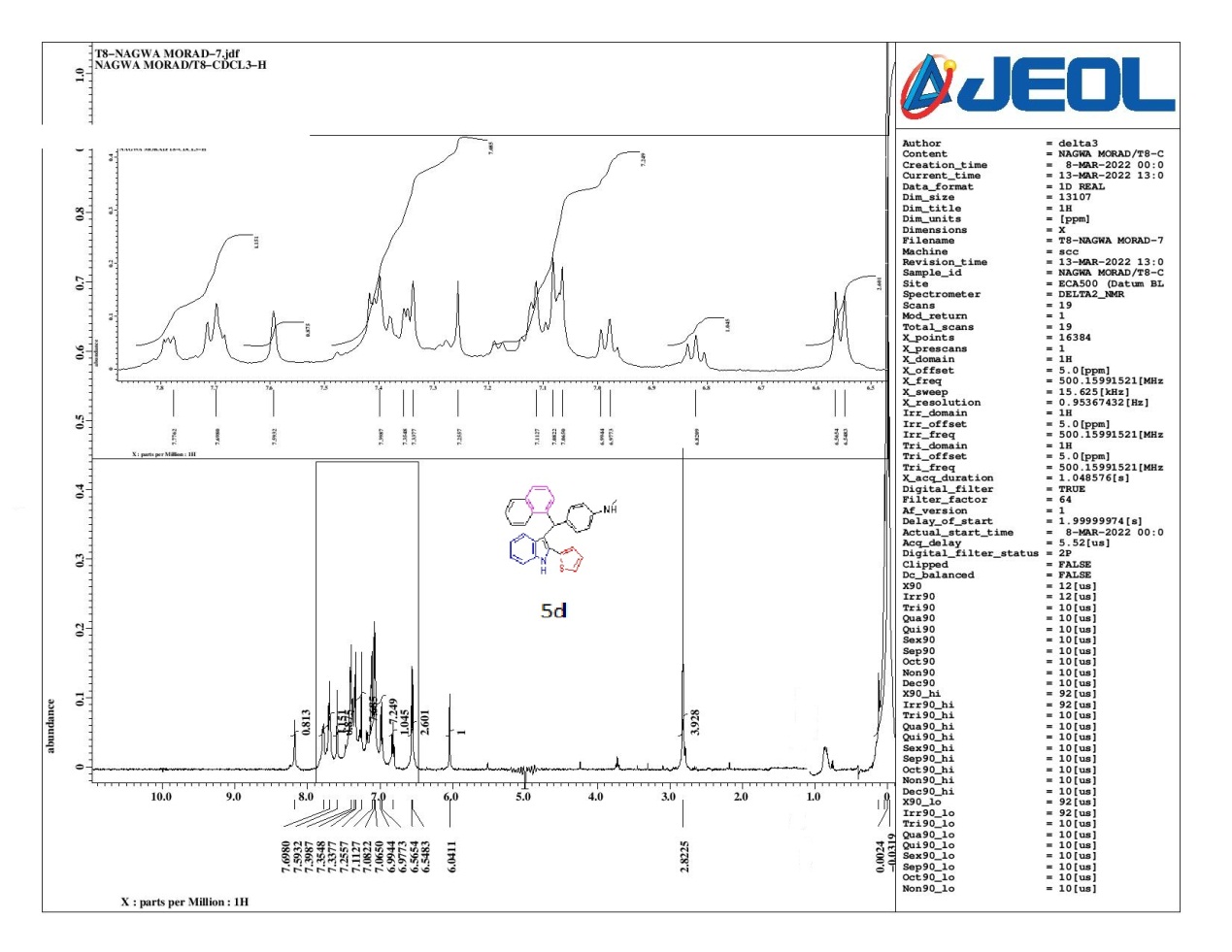


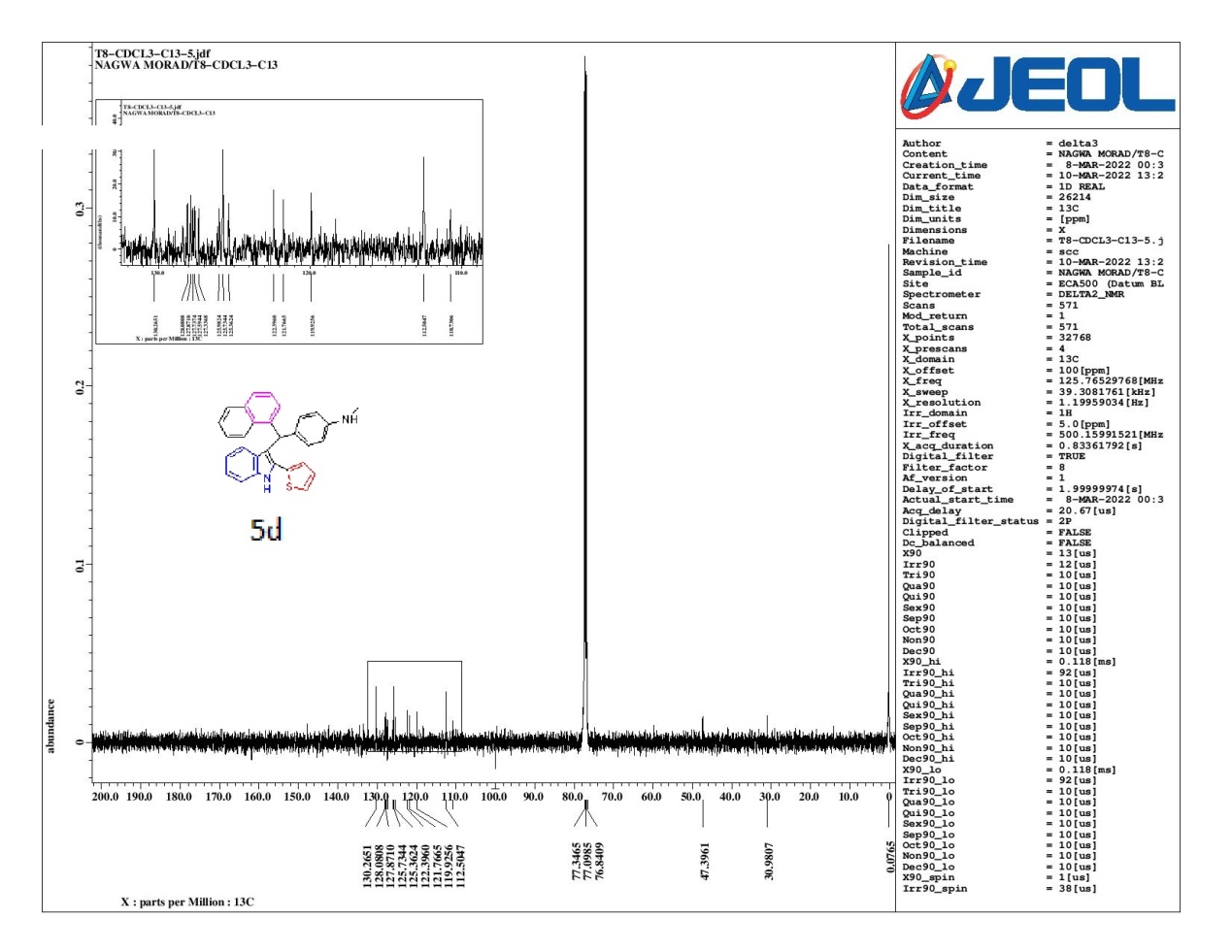


^1^H NMR and^13^C NMR for compound 5e


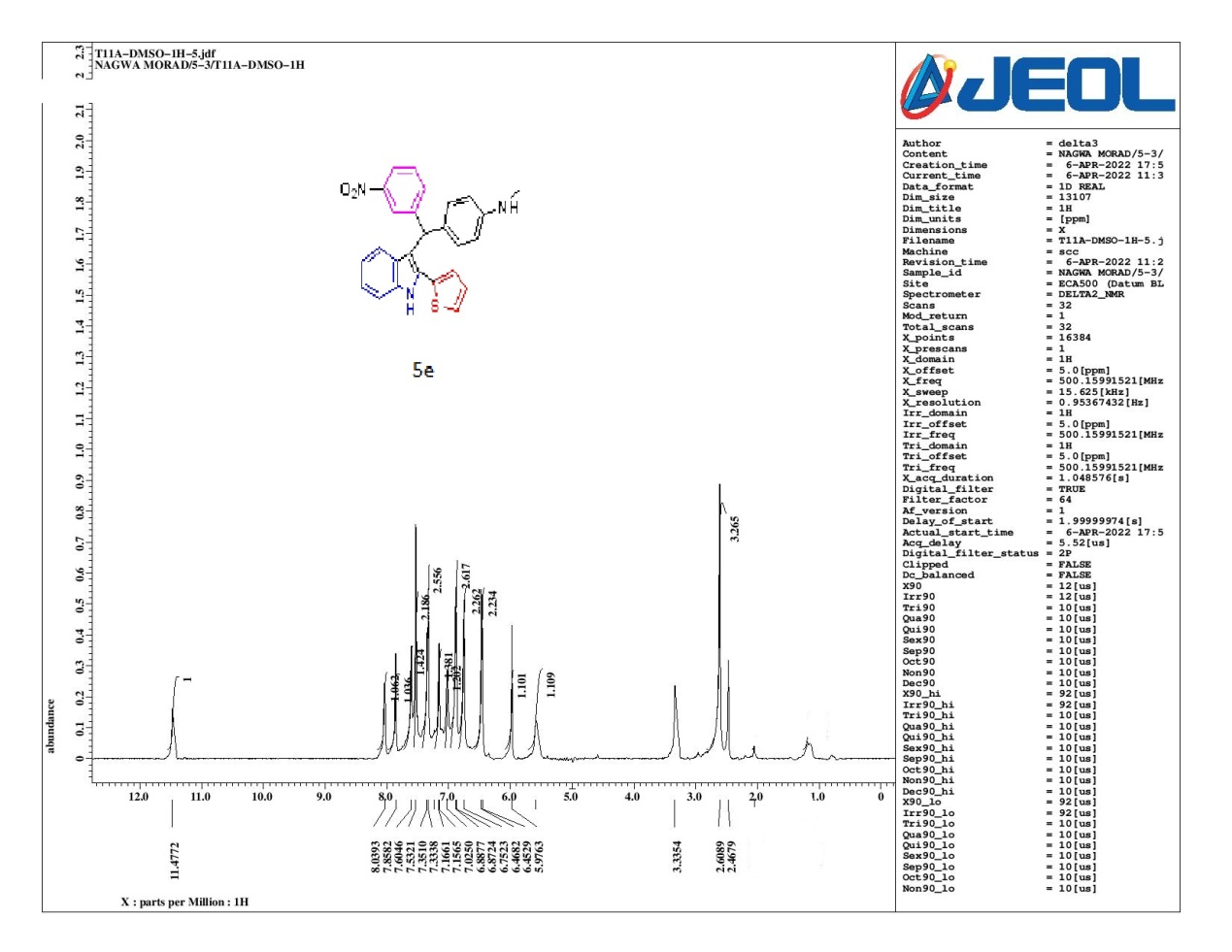


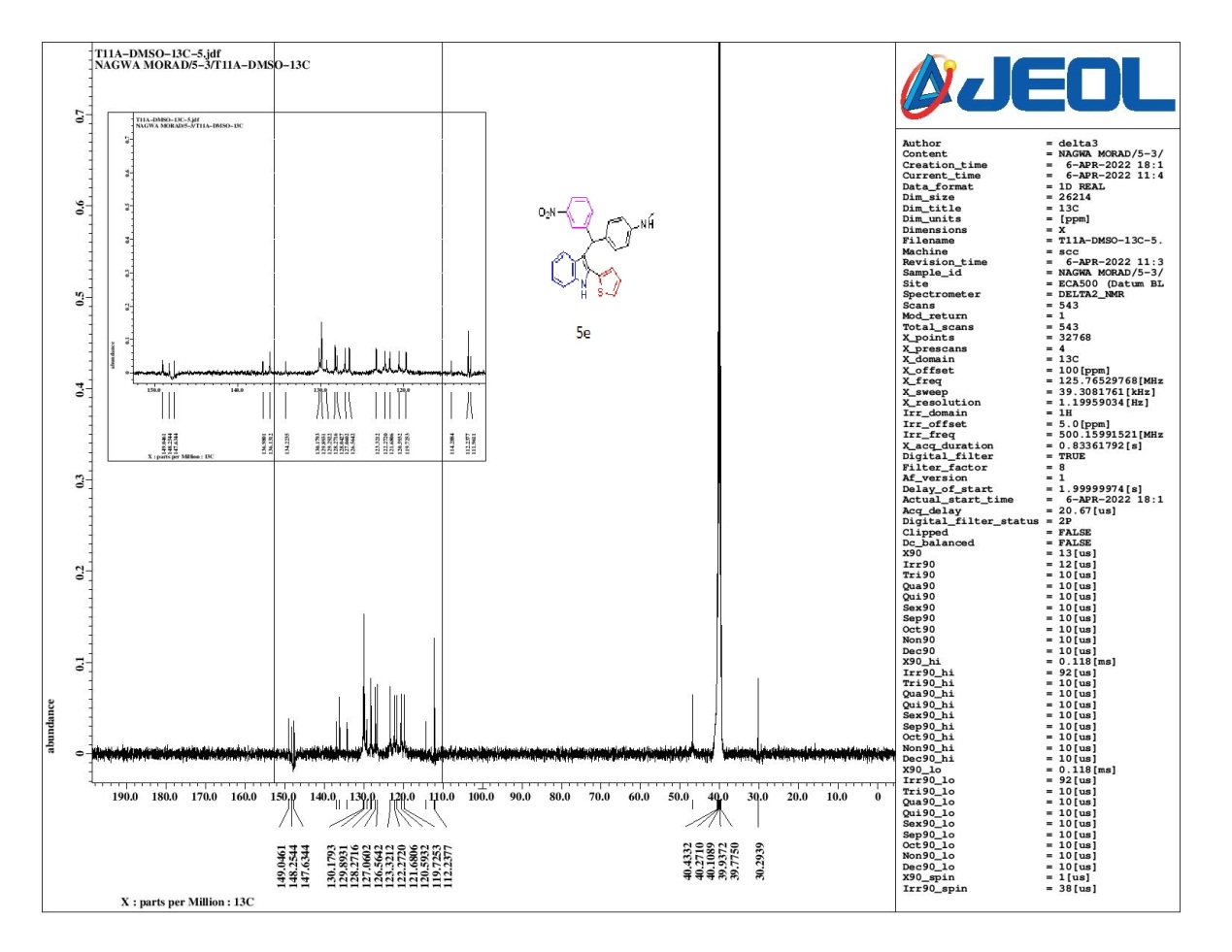


|  |  | HCT-116 | | HT-29 | | RPE-1 | |
| --- | --- | --- | --- | --- | --- | --- | --- |
|  | Cp | %cytotoxicity  (at100µg/ml) | IC_50_ (µg/ml) | %cytotoxicity  (at100µg/ml) | IC_50_ (µg/ml) | %cytotoxicity  (at100µg/ml) | IC_50_ (µg/ml) |
| 1 | 4a | 97.17±0.9 | 5.087±0.074 | 69.77±2.4 | 80.74 | 87.5±2.7 | ~ 49.47±2.7 |
| 2 | 4b | 95.5±1 | 16.37±0.047 | 22.1±1.9 | ----- | 94.3±2.3 | 45.56±0.06 |
| 3 | 4c | 99.63±0.89 | 6.183±0.053 | 86 ±1.5 | 37.68 | 101.7±0.6 | 14.91±0.02 |
| 4 | 4d | 28.13±1.9 | ---- | 1.8±0.3 | --- | 22.3±0.5 | ----- |
| 5 | 4e | 98.67±0.9 | 32.96±0.074 | 76.3±5 | 39 | 94.6±2.6 | ~ 48.8±2.6 |
| 6 | 4f | 45.7±1.8 | ----- | 6.9±1.8 | ---- | -91.26±9.85 | ---- |
| 7 | 4g | 99.17±0.95 | 3.566±0.072 | 85.75±11.6 | 24.39 | 96.5±1.3 | ~ 46.35±1.3 |
| 8 | 4h | 60.6±4.5 | 76.11±0.053 | 26.3±5 | ---- | 59.7±8 | 90.71±0.03 |
| 9 | 4i | 86.4±1.8 | 22±0.052 | 22.78±3.3 | ---- | 80.6±8.9 | ~ 50.28±8.9 |
| 10 | 4j | 97.87±1.2 | 25.54±0.051 | 11.9±0.5 | --- | 93.74±3.8 | 40.78±0.04 |
| 11 | 4k | 93.5±5.5 | 21.79±0.065 | 87.57±10.4 | 44.2 | 97.7±1.3 | ~ 90.11±1.3 |
| 12 | 5a | 89.17±2.2 | 56.2±0.032 | 8.76±1.17 | --- | 99.02±0.9 | 38.58±0.2 |
| 13 | 5b | 55 ±4.3 | 48.91±0.193 | 66.5±2.2 | 66.09 | 100.6±0.47 | ~ 52.23±0.47 |
| 14 | 5d | 85.77±3.2 | 69.95±0.043 | 13.1±2 | --- | 98.87±1.7 | 49.29±0.36 |
| 15 | 5e | 94.9±4.9 | 57.65±0.032 | 7.9±0.86 | ---- | 73.3±3.4 | ~ 50.2±3.4 |

***S3: Biological Studies***

**Table 1:** In Vitro cytotoxic Activity of analogues on different cancer monolayer cell lines.

***S4: Docking simulation study***


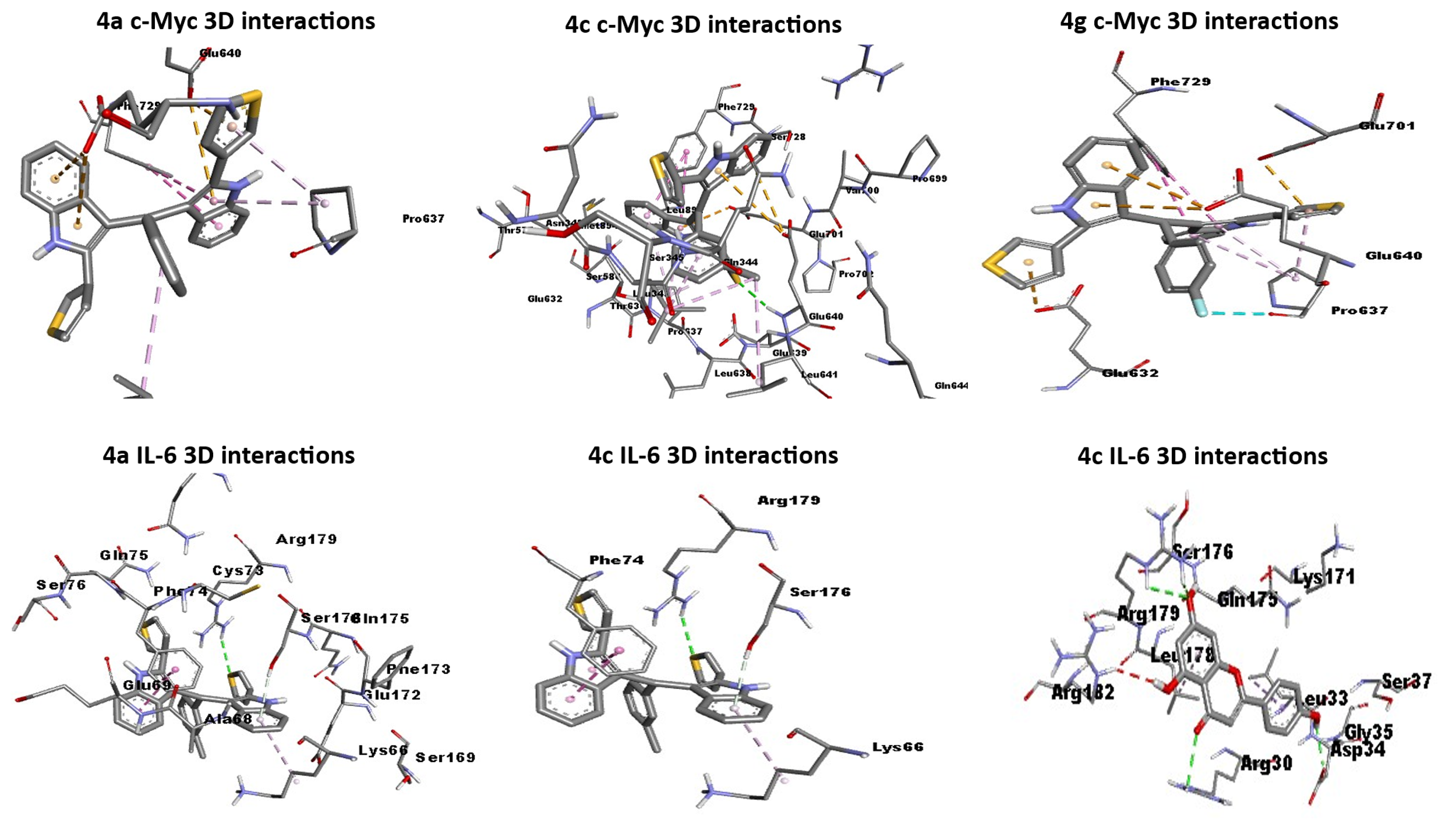


**Figure1. Amino acids involved in the 3D interactions of the best docked complexes inside the binding pocket of C-Myc and IL-6 proteins**

***S5: Cell cycle analysis and quantitative RT-PCR***


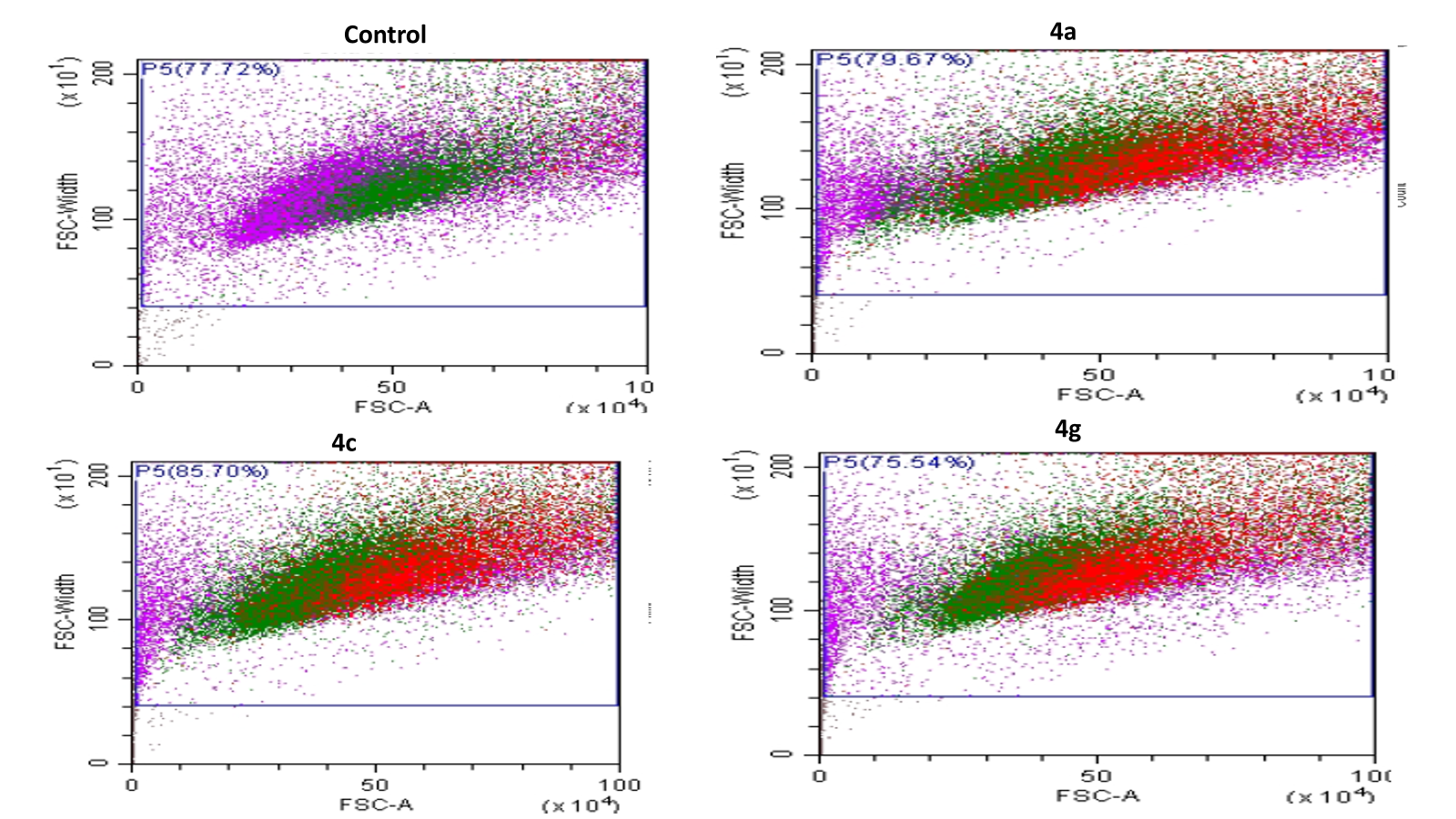


**Figure2.** Cell cycle phases distribution using PI staining and Relative DNA content for HCT-116 cells. (A) untreated HCT-116 cells; (B) HCT-116cells treated with compound **4a**; (C) HCT-116 cells treated with compound **4c** ; (D) HCT-116 cells treated with compound **4g.**
